# Integrated mechanism for the generation of the 5′ junctions of LINE inserts

**DOI:** 10.1093/nar/gku1067

**Published:** 2014-11-06

**Authors:** Katsumi Yamaguchi, Masaki Kajikawa, Norihiro Okada

**Affiliations:** 1 Graduate School of Bioscience and Biotechnology, Tokyo Institute of Technology, 4259-B-15 Nagatsuta-cho, Midori-ku, Yokohama, Kanagawa 226-8501, Japan; 2 Department of Life Sciences, National Cheng Kung University, Tainan 701, Taiwan; 3 Foundation for Advancement of International Science, Tsukuba 305-0821, Japan

## Abstract

To elucidate the molecular mechanism of the integration of long interspersed elements (LINEs), we characterized the 5′ ends of more than 200 LINE *de novo* retrotransposition events into chicken DT40 or human HeLa cells. Human L1 inserts produced 15-bp target-site duplications (TSDs) and zebrafish ZfL2-1 inserts produced 5-bp TSDs in DT40 cells, suggesting that TSD length depends on the LINE species. Further analysis of 5′ junctions revealed that the 5′-end-joining pathways of LINEs can be divided into two fundamental types—annealing or direct. We also found that the generation of 5′ inversions depends on host and LINE species. These results led us to propose a new model for 5′-end joining, the type of which is determined by the extent of exposure of 3′ overhangs generated after the second-strand cleavage and by the involvement of host factors.

## INTRODUCTION

Long interspersed elements (LINEs) are transposable elements that are widely distributed in eukaryotic genomes (1); as such, they substantially affect genome complexity and evolution (2). LINEs are classified into clades based on the phylogenetic analysis of their sequences, and so far ∼30 clades of LINEs are identified (3). These elements mobilize and amplify their own sequences by a mechanism called retrotransposition. LINEs are 4–7 kbp in length and typically encode two open reading frames (ORFs), ORF1 and ORF2, both of which are important for efficient LINE retrotransposition (4,5). ORF1 protein (ORF1p) has nucleic acid binding activity and nucleic acid annealing activity, although the role of those activities in retrotransposition is not well understood (5–10). ORF2 protein contains an endonuclease (EN) and a reverse transcriptase (RT) domain (4,5). During retrotransposition, LINEs are first transcribed into mRNA from which the LINE-encoded proteins are translated. Next, the LINE mRNA and proteins form a complex (11,12) and move to a target site on a host chromosome where the LINE-encoded EN nicks a strand on the host DNA duplex. The LINE-encoded RT then reverse-transcribes the LINE mRNA using the 3′ hydroxyl group generated by the nick as a primer. This reaction, called target-primed reverse transcription (TPRT) (13,14), connects the 3′ end of the LINE and the target genomic DNA. Many LINEs including those of the L2 clade are considered to require a specific sequence at the 3′ end of their RNA to initiate TPRT, whereas mammalian LINEs of the L1 clade do not; the former and latter LINEs are called stringent and relaxed types, respectively (15). The second strand of the target site also must be cleaved to complete integration of the newly synthesized LINE DNA into the host chromosome. The position of the second-strand cleavage is considered to define which kind of target-site alteration (TSA) is created at the integration site (16). According to the prevailing model (16), the second-strand cleavage downstream of the initial first-strand nick generates a target-site duplication (TSD), cleavage at the same site generates a blunt-end joining (BEJ) and cleavage upstream generates a target-site truncation (TST). Here we call this the ‘random-cleavage model’. However, Ichiyanagi and Okada previously showed that there are LINE clade-specific TSD peaks regardless of the host (17). In the case of L1 clade elements, for example, the TSD peak length is 13–15 bp in a variety of hosts, such as human, cow, opossum and zebrafish. In the case of L2 clade elements, TSDs have a peak length of 3–5 bp in opossum and zebrafish, which is very similar to the case of CR1 clade elements. In the case of RTE clade elements, a peak of 10–12 bp is apparent. These observations indicate that TSD is predominant in TSAs and suggest its length is dictated by the LINE species. That is, the site of the second-strand cleavage relative to the first-strand nick appears to be specified at a unique position depending on the LINE. These data are apparently inconsistent with the random-cleavage model. The exact mechanism of the integration of the LINE 5′ end into the host chromosome, however, remains to be elucidated.A DNA double-strand break (DSB) is likely to be generated at the target site during LINE retrotransposition. In fact, overexpression of human LINE L1 in mammalian cultured cells induces DSBs in the host chromosomal DNA (18). Several host-encoded DNA repair proteins are shown to be involved in LINE retrotransposition via several cultured cell assays (18–23). For example, the proteins of the non-homologous end-joining pathway (24), which predominates in DSB repair in vertebrate cells, are positively involved in efficient LINE retrotransposition (20). In contrast, the ERCC1/XPF EN, which functions in nucleotide excision repair, is suggested to have a limiting effect on retrotransposition (19). In the case of the ataxia telangiectasia mutated (ATM) protein, which is a protein kinase involved in cellular responses to DSBs, two reports show inconsistent results; one suggests its requirement for retrotransposition in mammalian cultured cells (23), and the other suggests its limiting effect on retrotransposition in neuronal cells (18). This may indicate that the role of ATM in retrotransposition is different among different cell types. Furthermore, Morrish *et al.* showed that a defect of a non-homologous end-joining protein enhances abnormal retrotransposition, i.e. EN-independent retrotransposition in cultured cells (21–23). These reports suggest that the chromosomal target site with the TPRT intermediate is likely recognized as a break and is repaired by host proteins, resulting in the joining of the 5′ end of LINE with the host genomic DNA to complete retrotransposition (16,18–20).

The 5′ region of LINE elements is often truncated (5′ truncation) or inverted (5′ inversion) via retrotransposition. These structures render LINEs incapable of further retrotransposition. Several studies have shown that L1 elements with 5′ truncation frequently exhibit microhomology (MH) at the 5′ junction, whereas those of full length are directly joined (direct joining, DJ; 25,26). These reports suggest that there are at least two distinct mechanisms for the 5′-end joining of the full-length and 5′-truncated L1 elements. In addition, other bioinformatic analyses have demonstrated that short TSDs (1–9 bp) are significantly more abundant in 5′-truncated L1 elements than in full-length and 5′-inverted L1 elements in the human genome (25), suggesting that the generation of TSAs also correlates with the structure of L1 elements.

The zebrafish genome contains more than 40 families of L2 clade LINEs. One of these, ZfL2-2, also called CR1-2_DR (27), is retrotransposition-competent in cultured cells (20,28). Approximately half of ZfL2-2 elements in zebrafish have several extra nucleotides inserted at the 5′ junction (5′ EXs) (29), suggesting that 5′-end joining involving extra nucleotides is a predominant pathway in the homologous system of zebrafish. On the other hand, 5′ EXs were rarely observed when ZfL2-2 was retrotransposed in HeLa-RC cells (29,30) used in our laboratory (28). Thus, the generation of 5′ EXs appears to depend on host factors, although how the host factors are involved has not been elucidated. We recently analyzed the 5′ junctions of ZfL2-2 inserts retrotransposed in human HeLa-RC and chicken DT40 cells (20,30). These analyses demonstrated that a large fraction of the 5′ EXs are derived from their flanking sequences, prompting us to hypothesize that, during retrotransposition, the 5′ EXs are generated to make a stable synapsis between the ends of the LINE and genomic DNAs prior to completion of the 5′-end joining (20).

Although analyses of heterologous systems may provide valuable information regarding the molecular mechanism of the 5′-end joining of LINE retrotransposition, most investigations have been limited to the analysis of human L1 inserts in human cells (16,31,32). Here, we compared the inserts generated by three distinct LINEs retrotransposed in two different host cell types to investigate the correlation among insert length, TSA type and features of the 5′ junction (5′ EX, MH and DJ). The information obtained in these analyses led us to propose a general model for the 5′-end joining of LINE inserts—applicable to any LINE species and host—that is based on the extent of exposure of the 3′ overhang generated by the second-strand cleavage of the target-site DNA.

## MATERIALS AND METHODS

### Plasmid construction

Detailed plasmid sequences are shown in Supplementary Table S1.

The fragments encoding human L1.3 5′ UTR, L1.3 ORFs, L1.3 3′ UTR and *mneoI_400_/ColE1* were inserted into the multicloning site of vector pCEP4. To make the pLEmH plasmid containing wild-type (WT) L1.3 and the *mneoI_400_/ColE1* cassette, we introduced three mutations into three *Hind*III sites of p*CEP4/L1.3mneoI_400_/ColE1*, which was donated by Moran (16), without synonymous changes.TK109-17 contains the zebrafish WT ZfL2-1 element with a *mneoI_400_/ColE1* cassette and 3 × FLAG DNA juxtaposed to the 5′ end of the ZfL2-1 ORF1 sequence and expresses WT ZfL2-1. The sequence fragments of 3 × FLAG DNA, ZfL2-1 5′ UTR, ZfL2-1 ORFs, ZfL2-1 3′ UTR and *mneoI_400_/ColE1* were inserted into the multicloning site of pCEP4.Nb2A3-2 contains the zebrafish WT Nimb-2_DR element with a *mneoI_400_/ColE1* cassette and expresses WT Nimb-2_DR. The Nimb-2_DR consensus sequence was obtained from the Zebrafish genome database (http://genome.ucsc.edu; the July 2010 zebrafish (*Danio rerio*) Zv9 assembly) and the University of California, Santa Cruz, Genome Browser website and was amplified from zebrafish genomic DNA by polymerase chain reaction. The sequence fragments of Nimb-2_DR 5′ UTR, Nimb-2_DR ORFs, Nimb-2_DR 3′ UTR and *mneoI_400_/ColE1* were inserted into the multicloning site of pCEP4.

### Sequence analysis of LINE integrants

Retrotransposition assays in chicken DT40 cells (20,33) and in human HeLa-RC cells (28) were performed using the pLEmH, TK109-17 and Nb2A3-2 constructs. The integrants of the LINEs retrotransposed in these cells were isolated as described previously (20). Briefly, DT40 cells were transfected with one of the LINE expression vectors (15 mg), pLEmH, TK109-17 or Nb2A3-2. Transfection of each expression vector was carried out by electroporation at 200 V and 960 mF, using the GENE Pulser (Bio-Rad). After the transfected cells were incubated at 33°C for 3 days, the electroporated cells (∼1 × 10^6^ cells per dish) were plated in soft agarose medium containing G418 (1.6 mg/ml) to detect retrotransposition. After incubation for 11 days at 37°C, DT40 cell clones derived from each G418-resistant colony produced by pLEmH, TK109-17 or Nb2A3-2 were cultured separately until the total number of cells reached ∼1 × 10^6^ cells per clone.

HeLa-RC cells (2 × 10^5^ cells/well) were seeded in 6-well dishes. After 1 day, the cells were transfected with 500 ng plasmid DNA using FuGENE6 Transfection Reagent (Roche) according to the manufacturer's instructions. The cells containing the plasmid (pLEmH, TK109-17 or Nb2A3-2) were selected with hygromycin (200 μg/ml) for 5 days. The hygromycin-resistant (HygR) cells were trypsinized and reseeded into new 100-mm dishes and grown in medium with 400 μg/ml G418. After a 10-day incubation, HeLa-RC cell clones derived from each G418-resistant colony produced by pLEmH, TK109-17 or Nb2A3-2 were cultured separately until the total number of cells reached ∼1 × 10^6^ cells per clone.

Genomic DNA was isolated from each clone using the GenElute mammalian genomic DNA miniprep kit (Sigma). Genomic DNA (20 μg per clone) was digested with 75 U of *Hind*III for 6 h at 37°C. The digested DNA (∼20 μg) was then self-ligated overnight with T4 DNA ligase (350 U) in 500 μl volume at 16°C. Ninety percent of the circular DNA was incorporated in *Escherichia coli* ElectroMAX DH10B Cells (Invitrogen) or NEB 10-beta Electrocompetent *E. coli* (New England Biolabs) by electroporation with the GENE Pulser Xcell (Bio-Rad) under conditions of 2500 V, 25 mF and 100 V, and the electroporated cells were plated on plates containing kanamycin (70 mg/ml). Circular DNA containing a *mneoI_400_/ColE1*-marked L1.3, ZfL2-1 or Nimb2_DR insertion (with its flanking chicken or human genomic DNA) was isolated from the kanamycin-resistant cells. Each isolated insert was sequenced using the appropriate primers. We manually conducted homology searches of the sequences flanking each insertion using the consensus sequence of each LINE and the reference genomic DNA to identify the preintegration site in the chicken (http://genome.ucsc.edu; the May 2006 chicken (*Gallus gallus*) WUGSC 2.1/galGal3 assembly) or human (http://genome.ucsc.edu; the Human (*Homo sapiens*) Feb. 2009 (GRCh37/hg19) assembly) genome database. We used the data of individual retrotransposable L1.3 inserts distinguishable from endogenous L1. Only the data for L1.3 integrants in HeLa cells and in HCT116 cells were extracted from Gilbert *et al.* (31) and Symer *et al.* (32), respectively. A list of all the sequences and summaries of LINE inserts identified in this work are available in Supplementary Tables S2 and S3, respectively.

## RESULTS

### Distribution pattern of L1 TSAs differs in different human cell lines

**Figure 1.**
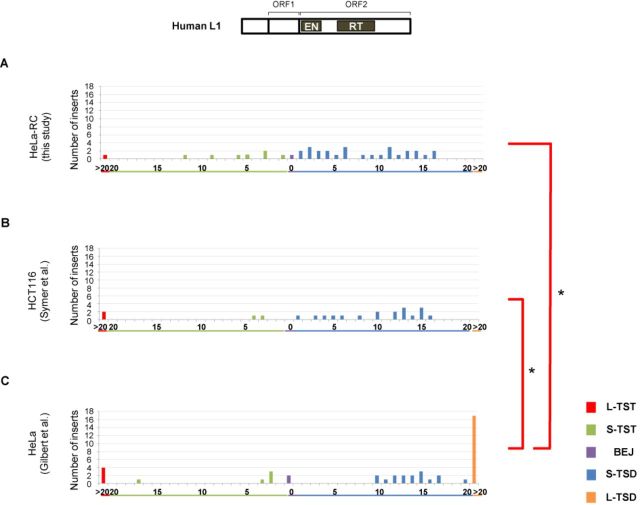
Patterns of L1 TSAs vary according to human cell type. Length distributions of L1 TSAs in a variety of human cells. TSAs were categorized into five types: long TST (L-TST, >20 bp), short TST (S-TST, ≤20 bp), BEJ (0 bp), short TSD (S-TSD, ≤20 bp) and long TSD (L-TSD, >20 bp). L1 5′ inversions are excluded. (A) Length distribution of L1 TSAs in HeLa-RC cells. (B) Length distribution of L1 TSAs in HeLa cells reported by Gilbert *et al.* (31), from which the data for L1.3 TSAs in HeLa cells were extracted. (C) Length distribution of L1 TSAs in HCT116 cells by Symer *et al.* (32), from which the data for L1.3 TSAs in HCT116 cells were extracted. Statistical analysis was performed to compare the distribution of the five types of TSAs between all pairwise combinations of cell types using the Fisher exact test with Bonferroni correction, and *P*-values less than 0.05 are indicated by asterisks. A schematic representation of the human L1 structure is shown at the top.

**Figure 2.**
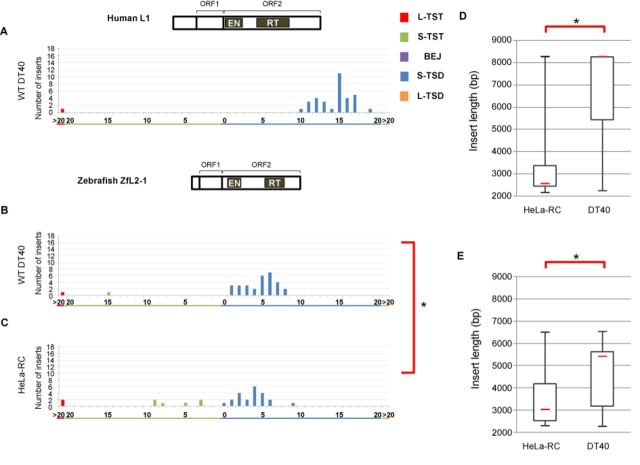
Structures of the 5′ junctions of human L1 and zebrafish ZfL2-1 are distinct. (A) Length distribution of L1 TSAs in DT40 cells. (B) Length distribution of ZfL2-1 TSAs in DT40 cells. (C) Length distribution of ZfL2-1 in HeLa-RC cells. Statistical analysis was performed using the Fisher exact test for each TSA category, and *P*-values less than 0.05 are indicated by asterisks. (D) Lengths of L1 inserts in HeLa-RC cells and DT40 cells. (E) Lengths of ZfL2-1 inserts in HeLa-RC cells and DT40 cells. Box-and-whisker plots show the median (red line), the first and third quartiles and the upper and lower limits of the insert lengths. *P*-values less than 0.05 (Mann–Whitney U test) are indicated by asterisks. Schematic representations of the human L1 and zebrafish ZfL2-1 structures are shown above the respective length distribution graphs.

**Figure 3.**
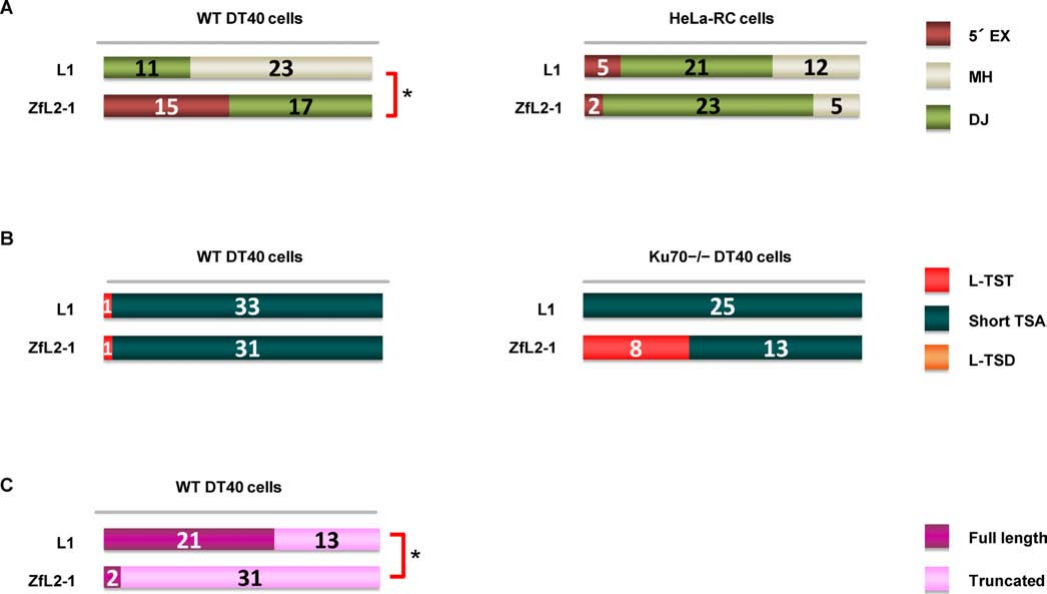
5′-end joining in retrotransposition proceeds via one of two pathways: annealing or DJ. (A) Features of the 5′ junctions of L1 and ZfL2-1 inserts categorized as 5′ EX, MH or DJ in DT40 cells (left) and HeLa-RC cells (right). (B) Long (>20 nt) and short (≤20 nt) TSAs, as categorized in the Figure 1 legend, in WT DT40 cells (left) and DT40 Ku70^−/−^ cells (right). (C) Full-length versus truncated L1 and ZL2-1 inserts in DT40 cells. *P*-values less than 0.05 (Fisher exact test) are indicated by asterisks.

**Figure 4.**
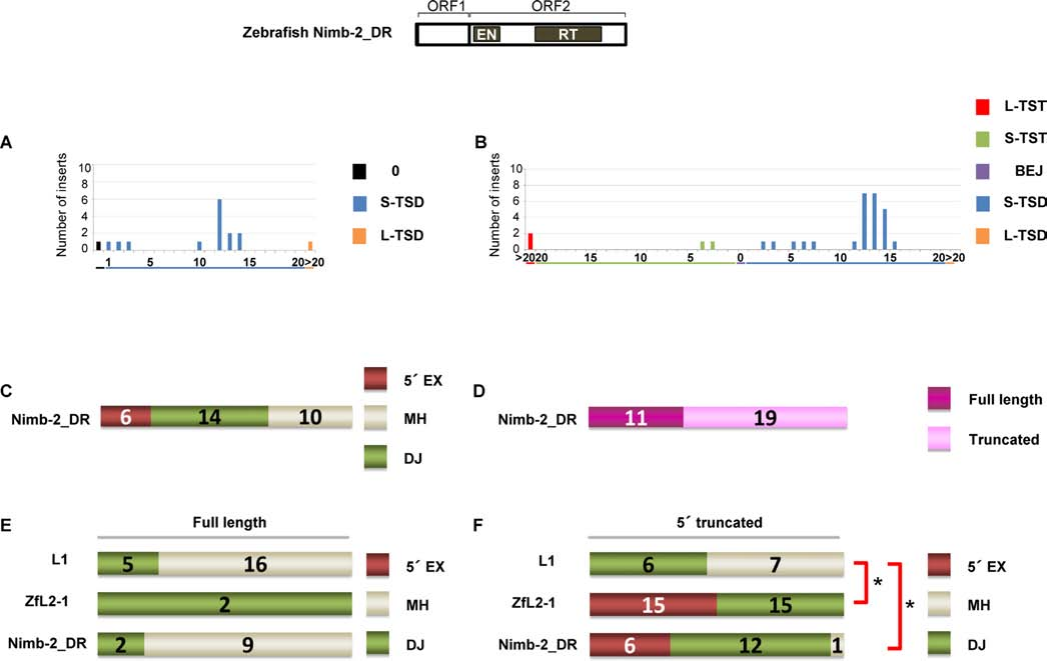
Zebrafish LINE Nimb-2_DR inserts in DT40 cells exhibit characteristics of the two types of 5′-end joining. (A) Length distribution of Nimb-2_DR TSAs in the zebrafish genome. (B) Length distribution of Nimb-2_DR TSAs in DT40 cells. (C) Features of the 5′ junctions of Nimb-2_DR inserts categorized as 5′ EX, MH or DJ in DT40 cells. (D) Full-length versus truncated Nimb-2_DR inserts in DT40 cells. (E) Features of the 5′ junctions of full-length inserts of L1, ZfL2-1 and Nimb-2_DR. (F) Features of the 5′ junctions of 5′-truncated inserts of L1, ZfL2-1 and Nimb-2_DR. *P*-values less than 0.05 (Fisher exact test) are indicated by asterisks. A schematic representation of the zebrafish Nimb-2_DR structure is shown at the top.

**Figure 5.**
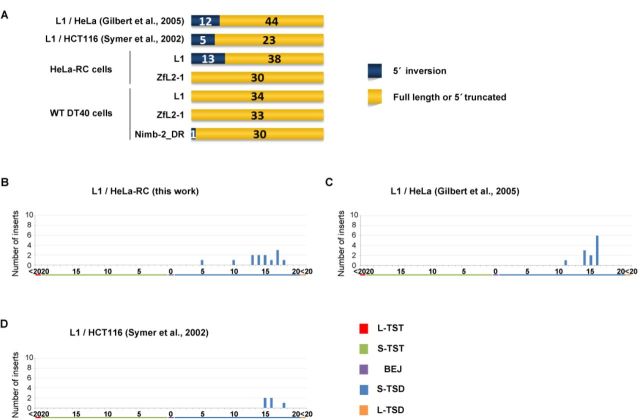
5′ inversions are frequently observed in L1 inserts in human cells with a peak of ∼15 bp TSDs. (A) The structures of LINE inserts categorized as 5′ inversion, full length or 5′ truncation in human cells (top) and DT40 cells (bottom). (B–D) Length distributions of TSAs of 5′ inversions in cultured cells. TSAs were categorized into five types as indicated in Figure 1. (B) Length distribution of TSAs of L1 inversions in HeLa-RC cells. (C) Length distribution of TSAs of L1 inversions in HeLa cells by Gilbert *et al.* (31). (D) Length distribution of TSAs of L1 inversions in HCT116 cells by Symer *et al.* (32).

TSAs generated at the LINE insertion sites are considered to reflect the mechanism of the 5′-end joining of inserts to the genomic DNA; a model of how LINE retrotransposition generates the various types of TSAs has been proposed (16). Because longer TSAs are considered to be in a different category than shorter TSAs seen in the majority of LINE inserts (16), we categorized TSAs into five types: long TST (L-TST, >20 bp), short TST (S-TST, ≤20 bp), BEJ (0 bp), short TSD (S-TSD, ≤20 bp) and long TSD (L-TSD, >20 bp). It should be noted that inserts with 5′ inversions were not included in the analysis shown in Figures 1–4 because most 5′ inversions were observed with inserts of human L1 but not with those of other LINEs used in this study. In the later section (most 5′ inversions are observed with L1 inserts in human cells; Figure 5), the inserts with 5′ inversions are analyzed and discussed.

To examine the frequency of TSA types of human L1, we analyzed 38 L1 inserts retrotransposed in HeLa-RC cells (Figure 1A) (28). The length distribution of the L1 TSAs in HeLa-RC cells was very similar to that in human HCT116 cells obtained by Symer *et al.* (32) (Figure 1B). This was, however, significantly different from that in HeLa cells studied by Gilbert *et al.* (31) (Figure 1C). The analysis of L1 TSAs found by Gilbert *et al.* (31) revealed many L-TSDs but no TSDs ranging from 1 to 9 bp. These data show that the distribution of L1 TSAs can differ among different human cell lines even if the same human LINE is used for the analysis, suggesting that the host factor(s) that are differentially expressed or regulated among those cell lines are involved in the mechanism of TSA generation. Thus, comparison of the structure of LINE inserts mobilized in different cell lines produces valuable information to elucidate the molecular mechanism of LINE integration.

### Generation of the TSA may involve two steps: cleavage of the second strand at a specific site and processing of the cleaved DNA

To address how a different cell line influences TSA generation, human L1 was retrotransposed in chicken DT40 cells, and 37 inserts were analyzed. Surprisingly, only 10- to 20-bp S-TSDs with a sharp peak at 15 bp were observed in these inserts, and other TSAs were not generated except for one L-TST (Figure 2A). This pattern suggests that, in DT40 cells, the second-strand cleavage of the target site occurs only within a ∼10 bp region of 10–20 bp downstream from the first-strand nick. We here suppose that such specific cleavage of the second strand occurs regardless of the cell type in which L1 retrotransposes, because it might result from the inherent character of a particular LINE. In this case, the generation of the shorter TSDs and TSTs in HeLa-RC cells can be explained by the processing of the 3′ overhang of ∼15 nt generated at the target site in HeLa-RC cells, but not in DT40 cells. In other words, host processing factor(s) are involved in the generation of TSAs, and which type of TSA is produced in a cell type depends on host factor activities. Hereafter we call this hypothesis the ‘specific-cleavage model’, in which the cleavage site of the second strand is strictly determined within a narrow region. In the case of human L1, it is about 15 bp downstream from the first-strand nick.

Next we examined what kind of TSA pattern was observed when a LINE different from human L1 was used. We previously isolated a retrotransposition-competent LINE, called ZfL2-1 or CR1-1_DR, from the zebrafish genome. Like human L1, it contains ORF1 and ORF2. Most inserts (93%) in DT40 cells have S-TSDs of 1–8 bp with a peak at ∼5 bp, and TSTs were rarely observed (Figure 2B). This result provides strong support for our specific-cleavage model and suggests that the cleaved region of the second strand is unique among different LINE species as we previously described (17): in the case of human L1, ∼15 bp downstream from the first-strand nick, and in the case of zebrafish ZfL2-1, ∼5 bp downstream. On the other hand, of 30 ZfL2-1 inserts retrotransposed in HeLa-RC cells, 21 (70%) have S-TSDs of up to 9 bp, and the remainder (30%) comprises one BEJ, six S-TSTs and two L-TSTs (Figure 2C). This pattern looks very similar to that of zebrafish LINE in DT40 in terms of appearance of a peak (∼4 bp), but the patterns are statistically different from each other (Figure 2B and C). This difference might be explained by the hypothesis that 3′ overhangs of ∼5 nt are frequently processed in HeLa cells. Taken together, these observations suggest that, during retrotransposition, human L1 and zebrafish ZfL2-1 generate 3′ overhangs of ∼15 and ∼5 nt, respectively, at the target site, and these overhangs are frequently processed in HeLa-RC cells, but not in DT40 cells.

There are LINE elements of various lengths because their 5′ regions are often truncated via retrotransposition. Given that the genomic DNA juxtaposed to the 5′ junction of the insert is processed to generate various types of TSAs in human cells, but not in DT40 cells, we speculate that the LINE insert length might also vary between HeLa-RC and DT40 cells. The lengths of the L1 inserts retrotransposed in the two cultured cell lines (Figures 1A and 2A) were compared in a box-and-whisker plot (Figure 2D). Significantly shorter L1 inserts were observed in HeLa-RC cells than in DT40 cells (Figure 2D). The lengths of ZfL2-1 inserts were also significantly shorter in HeLa-RC cells than in DT40 cells (Figure 2E). All these observations indicate that there is a correlation between the TSA pattern and the insert length in the two different LINEs. These results might indicate that host processing factor(s), which are probably more active in HeLa RC cells than in DT40 cells, are involved in the 5′-end joining of LINE retrotransposition.

### 5′-end joining in retrotransposition occurs by two pathways, one joined via annealing and the other joined directly

There are distinct features at the 5′ junction, which might be produced by different 5′-end joining pathways. We categorized the 5′ junctions into three types, i.e. 5′ EX, MH and DJ, and examined the frequency of each type (Figure 3A). First, we analyzed the 5′ ends of LINE inserts in DT40 cells. All the ZfL2-1 inserts observed in DT40 cells were joined with 5′ EXs or MHs (Figure 3A). In our previous study (30), we showed that a large proportion of the 5′ EXs of the zebrafish ZfL2-2 inserts originated from their flanking sequences, suggesting that the 5′ EXs as well as MHs are produced through annealing between the 5′ end of the LINE insert and the end of the genomic DNA generated by the second-strand cleavage. Accordingly, we categorized the pathway that makes the 5′ junction of the ZfL2-1 inserts in DT40 cells as annealing. In contrast, the majority of the human L1 inserts in DT40 cells showed DJ at the 5′ junction, which does not require annealing. From these results, we propose that at least two distinct pathways are present in DT40 cells to connect the LINE 5′ end to the genomic DNA: one is through base pairing of the ends of the LINE and genomic DNA, and the other does not use any base pairing at the site. Here, we suggest that the position of the second-strand cleavage dictates which pathway is selected. The second strand of the target site appears to be cleaved ∼15 bp downstream from the first-strand cleavage in L1 inserts, whereas it is cleaved ∼5 bp downstream from the first-strand cleavage in ZfL2-1 inserts. That is, in the case of ZfL2-1, the length of the double-stranded DNA (dsDNA) between the first- and second-strand cleavages is ∼5 bp, whereas, in the case of L1, it is ∼15 bp. The 5-bp dsDNA at the ZfL2-1 target site might be less stable than the 15-bp dsDNA at the L1 target site, making it easier for the former to be dissociated. The 3′ overhang exposed by the dissociation could then be used for subsequent base pairing to generate 5′ EXs or MHs.

Our data show the presence of two distinct pathways for the 5′-end joining in DT40 cells, the use of which is dependent on each LINE. On the other hand, in HeLa-RC cells most human L1 inserts were joined with MHs (55%) or with 5′ EXs (13%), and the rest were formed by DJ (Figure 3A). Similarly, most inserts of ZfL2-1 in HeLa-RC cells were also joined with MHs (77%) or with 5′ EXs (7%), and the rest were formed by DJ (Figure 3A). These data suggest that, in human cells, the 5′-end joining pathway is not dependent on the LINE species. The pathway(s) may be predominantly regulated by host factor(s) rather than the characteristic features of LINEs, for example, the difference in the site of the second-strand cleavage.

The Ku70 protein is a key factor that repairs DNA DSBs, and it binds to exposed DNA ends (24). We previously reported that L-TSTs of ZfL2-2 inserts were frequently observed in Ku70-deficient DT40 cells (20), suggesting that Ku70 prevents the generation of L-TSTs. To examine whether a similar phenomenon occurs when different LINEs were used, we analyzed inserts of both L1 and ZfL2-1 retrotransposed in Ku70^−/−^ DT40 cells. In the case of WT DT40 cells, almost all the inserts of both LINEs had short TSAs (≤20 bp) (Figure 3B). In the case of Ku70^−/−^ DT40 cells, however, L-TSTs of ZfL2-1 inserts were frequently observed, whereas L-TSTs of human L1 inserts were not detected (Figure 3B). L-TSTs of ZfL2-1 inserts observed in Ku70^−/−^ DT40 cells may be products that were processed with no protection by Ku70 of the 3′ overhangs exposed after the second-strand cleavage. These results suggest that the extent of exposure of the 3′ overhangs is different between L1 and ZfL2-1 in the absence of Ku70, and are consistent with our hypothesis that the extent of exposure of the 3′ overhangs at the target site dictates which 5′-end joining pathway is used.

Next, we compared the number of full-length and truncated LINE inserts in DT40 cells. The analysis showed that more than half of the L1 inserts were full length (62%), whereas full-length ZfL2-1 inserts were rarely observed (6%) (Figure 3C), suggesting that the extent of dissociation of dsDNA into 3′ overhangs may affect the LINE structure in DT40 cells. The 3′ overhangs exposed by dissociation of dsDNA may become good substrates for the host repair systems immediately to join the 5′ end of a LINE. On the other hand, full-length human L1 inserts may be produced without dissociation of dsDNA, allowing RT to reverse-transcribe to the end of the LINE transcript.

### Zebrafish LINE Nimb-2_DR inserts in DT40 cells exhibit characteristics of the two types of 5′-end joining

To explore whether a LINE other than human L1 and zebrafish ZfL2-1 uses one of the two distinct pathways in its 5′-end joining, we investigated features of the 5′ junctions of inserts of a zebrafish LINE, Nimb-2_DR, which belongs to the Nimb clade classified into I group (3). The full-length consensus sequence of Nimb-2_DR has the ORF1/ORF2 structure (34). We first manually obtained 16 Nimb-2_DR sequences from the zebrafish genome database (http://genome.ucsc.edu; the Jul. 2010 zebrafish (*Danio rerio*) Zv9 assembly) using the full-length consensus sequence of Nimb-2_DR (34) as a query. We classified the TSAs of these Nimb-2_DR elements into three categories; 0 (TST or BEJ), S-TSD and L-TSD. Approximately 70% (11 of 16) of Nimb-2_DR elements in the zebrafish genome had 10–15 bp TSDs with a peak at ∼12 bp (Figure 4A). Next, we retrotransposed Nimb-2_DR in DT40 cells, and 30 inserts were obtained. Consistent with the data of Nimb-2_DR elements in the zebrafish genome (Figure 4A), 70% of Nimb-2_DR inserts in DT40 cells had 10–15 bp TSDs with a prominent peak at 12–13 bp (Figure 4B). Of 30 inserts, 6 (20%) had 5′ EXs and 14 (47%) had MHs, respectively, both of which are mediated by annealing at the 5′ junction, and 10 inserts (33%) were directly joined (Figure 4C). The occurrence of all three types at the 5′ junction may indicate that Nimb-2_DR retrotransposes using both distinct pathways in the 5′-end joining in DT40 cells. Next, we analyzed the proportion of full-length Nimb-2_DR inserts in DT40 cells (Figure 4D). Thirty-seven percent (11 of 30) of Nimb-2_DR inserts were full-length, whereas 62% (21 of 34) and 6% (2 of 33) of L1 and ZfL2-1 inserts, respectively, were full-length, showing that the occurrence of full-length inserts of Nimb-2_DR was intermediate between that of L1 and ZfL2-1 (Figures 3C and 4D).

Because several reports proposed that the 5′-end joining of the full-length and 5′-truncated L1 inserts in human is conducted through distinct pathways (25,26), we analyzed the TSA distribution of the full-length and 5′-truncated inserts of the three LINEs in DT40 cells (Figure 4E and F). The 5′ ends of full-length L1 inserts were mainly formed by DJ (76%), as was also observed for 5′-truncated human L1 inserts (54%), although the ratio of MHs was higher in 5′-truncated L1 inserts (46%) than in full-length inserts (24%). In contrast, both full-length and 5′-truncated inserts of ZfL2-1 were joined exclusively with either 5′ EXs or MHs. Interestingly, the 5′ ends of full-length Nimb-2_DR inserts were mainly formed by DJ (82%), whereas most 5′-truncated Nimb-2_DR inserts were joined with either 5′ EXs or MHs (95%). The pattern of full-length Nimb-2_DR inserts is similar to that of human L1 inserts (Figure 4E), and the pattern of 5′-truncated Nimb-2_DR inserts is similar to that of ZfL2-1 inserts (Figure 4F). These observations indicate that Nimb-2_DR retrotransposes through the two distinct pathways in DT40 cells; one, which involves DJ, predominantly produces full-length inserts, and the other, which involves annealing, predominantly produces 5′-truncated inserts. Together with our hypothesis that the 5′-end joining is governed by the extent of dsDNA dissociation into the 3′ overhangs, the results regarding Nimb-2_DR inserts suggest that the extent of dissociation during Nimb-2_DR retrotransposition in DT40 cells is intermediate between that of L1 and ZfL2-1. Consistently, the TSD peak of Nimb-2_DR inserts in DT40 cells was ∼12 bp, which is between the TSD peaks of L1 (∼15 bp) and ZfL2-1 (∼5 bp).

### Most 5′ inversions are observed with L1 inserts in human cells

As was explained in the first paragraph of the Results, the data regarding inserts with 5′ inversion were excluded from the above analyses because 5′ inversion was detected only with L1 inserts in human cells except for one Nimb-2_DR insert in DT40 cells (Figure 5A). The occurrence of 5′ inversion in this specific situation indicates that its generation is dependent on features characteristic of L1 as well as those of host factors that might be differently expressed or regulated between human and chicken cells. Interestingly, most L1 5′ inversions occurred with TSDs distributed from 10 to 20 bp with a peak at ∼15 bp (Figure 5B–D), suggesting that the 5′-end joining pathway is distinct between L1 inserts with 5′ inversion and those without it (see Discussion).

## DISCUSSION

In this study, we retrotransposed three kinds of LINEs—human L1, zebrafish ZfL2-1 and zebrafish Nimb-2_DR—in human HeLa-RC and chicken DT40 cells, and more than 200 inserts were obtained and analyzed. The analysis of these sequences provided novel insights into the molecular mechanism of the 5′-end joining in LINE retrotransposition.

### Two distinct pathways for the 5′-end joining in LINE retrotransposition

**Figure 6.**
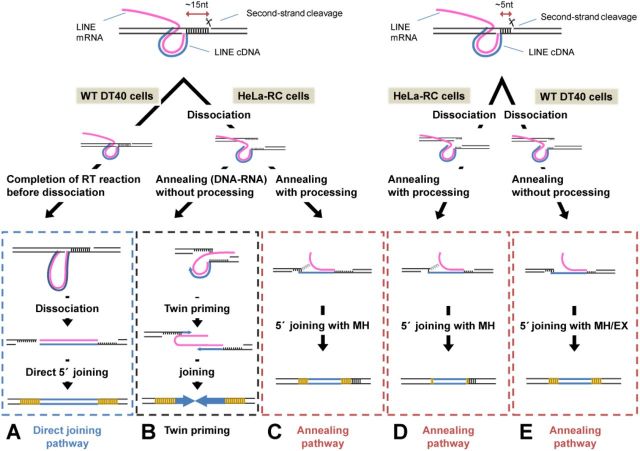
Mechanisms of 5′ joining of LINE inserts to the genomic target DNA. LINEs are joined by one of two pathways, each of which is governed by the extent of dissociation of the dsDNA flanked by the two cleavage sites as well as by the impact of host factors. A detailed explanation is provided in the text.

Our data clearly revealed that there are two distinct pathways, annealing and direct, to join the 5′ ends of LINEs with the genomic DNA. The two pathways appear to be used distinctively by each LINE in different cells (e.g. Figure 3A). In Figure 6, we show an integrated model of how the two distinct pathways are used in different situations. In this specific-cleavage model, the position of the second-strand cleavage relative to the first-strand nick is distinctively determined by each LINE though the molecular mechanism of the second-strand cleavage has not been well understood. In the case of human L1, the second-strand cleavage occurs ∼15 bp downstream from the first-strand nick, resulting in the generation of ∼15-bp dsDNA flanked by the two nicks at the target site (Figure 6, left side). On the other hand, in the case of zebrafish ZfL2-1, the second-strand cleavage occurs ∼5 bp downstream from the first-strand nick, resulting in ∼5-bp dsDNA flanked by the two nicks (Figure 6, right side). In DT40 cells, the ∼15-bp dsDNA of L1 is stable, and its base pairing is not dissociated, leading to the direct-joining pathway that mainly produces full-length elements (Figures 3A and C and 6A). The stable base pairing may provide the time for the L1 RT to reverse transcribe the L1 RNA fully, and the resulting blunt end of the LINE DNA-RNA hybrid makes it possible to join directly with the genomic DNA. In contrast, the ∼5-bp dsDNA of ZfL2-1 is unstable in DT40 and rapidly dissociates, leading predominantly to the annealing pathway to produce 5′-truncated elements (Figures 3A and C and 6E). The 3′ overhang of the genomic DNA generated by dissociation may be annealed with the synthesizing LINE DNA, which is most likely required for the annealing pathway. In short, in DT40 cells, the length of the dsDNA at the target site determines the extent of its dissociation, dictating which 5′-end joining pathway is selected (Figure 6A and E). On the other hand, in HeLa-RC cells, both of the ∼15- and ∼5-bp dsDNAs generated by L1 and ZfL2-1, respectively, appear to be dissociated to the same extent given that the features at the 5′ junctions are similar (Figure 3A). The predominance of MH at the 5′ junction suggests that the dissociation of the dsDNA mainly occurs before the completion of reverse transcription, leading to the annealing pathway in HeLa-RC cells (Figure 6C and D).

### Host factor(s) acting on the integration of LINE elements into the host chromosome

Our model postulates that the extent of dissociation of the genomic dsDNA at the target site is dependent on its length in DT40 cells, but not in HeLa-RC cells. This suggests the involvement of host factor(s) in the dissociation at least in HeLa-RC cells. In HeLa-RC cells, the retrotransposition intermediate with TPRT may be rapidly recognized by host repair system(s), resulting in the active dissection of the dsDNA followed by the annealing pathway. We observed that shorter inserts were more abundant in HeLa-RC cells than in DT40 cells, a finding that correlated with the broad range of TSAs in HeLa-RC cells as well as the narrow range of TSDs in DT40 cells (Figures 1A and 2A–E). This suggests that the DNA ends exposed by the active dissection in HeLa-RC cells are frequently processed by host processing factor(s), whereas the DNA ends exposed in DT40 cells are not processed. The activity of processing factor(s) acting on the TPRT intermediate may be highly stronger in HeLa-RC cells than in DT40 cells. Conversely, the activity of polymerase(s) acting on the TPRT intermediate may be stronger in DT40 cells than in HeLa-RC cells, because 5′ EXs are more frequently generated in DT40 cells than in HeLa-RC cells.

### Mechanism of 5′ inversion

In this study, 5′ inversion was only generated with human L1 retrotransposed in human cells (Figure 5A). 5′ inversion is believed to be generated by twin-priming in which reverse transcription of the LINE RNA is initiated at the two genomic ends generated by the first- and second-strand nicks (35). To initiate the reverse transcription from the second-strand nick (second reverse transcription), the dsDNA at the target site must be dissociated before completion of the reverse transcription proceeds from the first-strand nick (first reverse transcription). Interestingly, L1 inserts with 5′ inversion in human cells have longer TSDs of 10–20 bp than those without it (Figure 5B–D), suggesting that the 3′ overhang generated by the dissociation at the target site must be recognized by the L1 RT before it is recognized by host processing factor(s). Alternatively, shorter 3′ overhangs of L1 generated by processing may be unavailable for the initiation of the second reverse transcription. There is no 5′ inversion with L1 in DT40 cells, suggesting that the 15-bp dsDNA of L1 is not dissociated before the completion of the first reverse transcription. ZfL2-1 also does not produce any 5′ inversion in either HeLa-RC or DT40 cells, though its 5-bp dsDNA at the target site appears to be dissociated before the completion of the first reverse transcription. The 3′ overhang length of ∼5 nt generated by the dissociation may be too short to stably base pair with the LINE RNA, which is prerequisite for the initiation of the second reverse transcription. This reminds us that the L1 inserts with 5′ inversion in human cells have longer TSDs of 10–20 bp.

### Generality of the two pathways

Our data suggest that the annealing pathway is predominant in HeLa-RC cells and that the direct-joining pathway is minor if it exists at all (Figure 3A). However, Zingler *et al.* (26) previously reported that 5′-truncated L1 requires MH for the 5′-end joining and that full-length L1 does not require base pairing (i.e. DJ), according to the analysis of the L1 elements existing in the human genome. Kojima (25) showed that the TSD length distribution of full-length L1 in the human genome exhibits a sharp peak in comparison with that of 5′-truncated L1. In addition, among all the 66 full-length L1 elements analyzed by Beck *et al.* (36), we observed that they were flanked by TSDs of 10–20 bp with two exceptions. These reports suggest that the two distinct pathways for the 5′-end joining, annealing and direct, also act in human cells, although the annealing pathway appears to be predominant. Together with those reports, our study indicates that the 5′-end joining of LINEs is conducted through at least the two distinct pathways whose participation in retrotransposition differs among cell lines depending on host factor(s) and/or the position of the second-strand cleavage at the target site.

## SUPPLEMENTARY DATA

Supplementary data are available at NAR Online.

SUPPLEMENTARY DATA
