# Integrated mechanism for the generation of the 5′ junctions of LINE inserts

**DOI:** 10.1093/nar/gkv041

**Published:** 2015-01-29

**Authors:** K. Yamaguchi, M. Kajikawa, N. Okada

Nucleic Acids Res. 2014 Dec 1;42(21):13269–79. doi: 10.1093/nar/gku1067.

In the legends of Figures 3A, 4C, E and F, the authors have inverted MH (white instead of green) and DJ (green instead of white). New figures are shown below and should replace the figures originally published. The corrections do not affect the results and conclusion of the article. The authors wish to apologise to the readers and publisher for the inconvenience caused.

**Figure 3.**
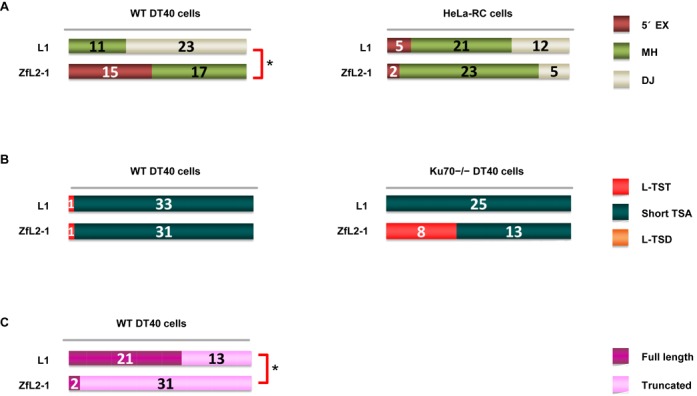
5′-end joining in retrotransposition proceeds via one of two pathways: annealing or DJ. (A) Features of the 5′ junctions of L1 and ZfL2-1 inserts categorized as 5′ EX, MH, or DJ in DT40 cells (left) and HeLa-RC cells (right). (B) Long (>20 nt) and short (≤20 nt) TSAs, as categorized in the Figure 1 legend, in WT DT40 cells (left) and DT40 Ku70−/− cells (right). (C) Full-length vs. truncated L1 and ZL2-1 inserts in DT40 cells. *P*-values less than 0.05 (Fisher exact test) are indicated by asterisks.

**Figure 4.**
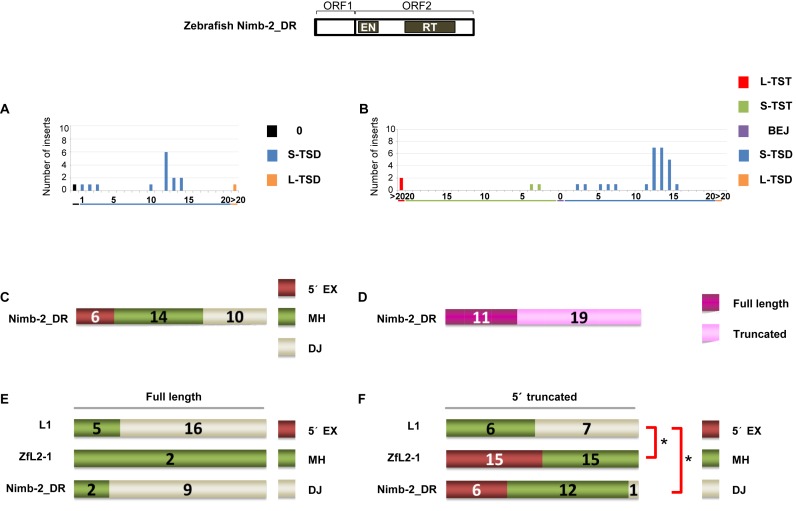
Zebrafish LINE Nimb-2_DR inserts in DT40 cells exhibit characteristics of the two types of 5′-end joining. (A) Length distribution of Nimb-2_DR TSAs in the zebrafish genome. (B) Length distribution of Nimb-2_DR TSAs in DT40 cells. (C) Features of the 5′ junctions of Nimb-2_DR inserts categorized as 5′ EX, MH, or DJ in DT40 cells. (D) Full-length vs. truncated Nimb-2_DR inserts in DT40 cells. (E) Features of the 5′ junctions of full-length inserts of L1, ZfL2-1, and Nimb-2_DR. (F) Features of the 5′ junctions of 5′-truncated inserts of L1, ZfL2-1, and Nimb-2_DR. *P*-values less than 0.05 (Fisher exact test) are indicated by asterisks. A schematic representation of the zebrafish Nimb-2_DR structure is shown at the top.

