# Supplementary material for: Integrated mechanism for the generation of the 5′ junctions of LINE inserts
Source: Nucleic Acids Res. 2014 Nov 6;42(21):13269–79. doi: 10.1093/nar/gku1067 (PMC4245944; doi:10.1093/nar/gku1067)
Supplement: SUPPLEMENTARY DATA [file supp_42_21_13269__index.html]

Integrated mechanism for the generation of the 5′ junctions of LINE inserts — Integrated mechanism for the generation of the 5′ junctions of LINE inserts — SUPPLEMENTARY DATA 

# Integrated mechanism for the generation of the 5′ junctions of LINE inserts

## SUPPLEMENTARY DATA

**Files in this Data Supplement:**

- SUPPLEMENTARY DATA
- SUPPLEMENTARY DATA
- SUPPLEMENTARY DATA
- SUPPLEMENTARY DATA
