# Supplementary material for: Integrated mechanism for the generation of the 5′ junctions of LINE inserts
Source: Nucleic Acids Res. 2014 Nov 6;42(21):13269–79. doi: 10.1093/nar/gku1067 (PMC4245944; doi:10.1093/nar/gku1067)
Supplement: SUPPLEMENTARY DATA [file supp_gku1067_nar-02442-v-2014-File003.pdf]

Supplemental Table S1

The Sequences of pLEmH, TK109.17, Nb2A3-2.

Supplemental Table S2

Sequences of the 3´ and 5´ LINE Inserts in Cultured Cells

The upper sequence indicates the 5´ end of the insert. The middle sequence indicates the pre-insertion site of human (designated ‘hs’) and chicken (designated ‘gg’) chromosomal DNA, the chromosome number, and direction (designated as +/–). The bottom sequence indicates the 3´ end of the insert. Chromosomal DNA, black; LINE, blue; 5´-inverted LINE, purple; extra nucleotides, red pink; TSD, bright green; TST, orange; 3´ terminal repeat, underline; abnormal 3´ terminal repeat, yellow; homologies, asterisks; microhomologies, gray boxes; italics, restriction site of HindIII.

Supplemental Table S3

Summary of LINE Inserts in Cultured Cells

LR indicates the length of the 3´ terminal repeat. An in the table under LR indicates poly(A) tail of unknown length. IL indicates the length of the insertion. 5´ MH indicates the length of the 5´ microhomology. 5´ EX indicates the length of the 5´ extra nucleotides. 3´ MH indicates the length of the 3´ microhomology. 3´ EX indicates the length of the 3´ extra nucleotides. TSA indicates the target-site alteration; a positive number is the length of target-site duplication; zero indicates blunt-end joining; a negative number is the length of target-site truncation. Chr position indicates the chromosome in which each insertion occurred. +/– indicates the direction of each insertion. Position indicates the nucleotide position of each insertion in the chromosome. ND, could not be determined.
