# Supplementary material for: Integrated mechanism for the generation of the 5′ junctions of LINE inserts
Source: Nucleic Acids Res. 2014 Nov 6;42(21):13269–79. doi: 10.1093/nar/gku1067 (PMC4245944; doi:10.1093/nar/gku1067)
Supplement: SUPPLEMENTARY DATA [file supp_gku1067_nar-02442-v-2014-File005.pdf]

Table S2

L1.3 / DT40 WT cells

pWLE23.1a

```
5´ end        GGGCTGGCCCTTTGTAGTAAGACGAATTTCAGATCTCTAGAAGCTGGGTACCAGCTGCT
              ********************************
gg_chr24+     GGGCTGGCCCTTTGTAGTAAGACGAATTTCAGCCAAGAGTATTTGCAGAATAACACCCT
                                *****************************************
3´ end         AAAAAAAAAAAAAAAAAAAAGACGAATTTCAGCCAAGAGTATTTGCAGAATAACACCCT
```

pWLE25.1a

```
5´ end        ACATGCTTTTAAGAGCATAGAGGTCAGATCTCTAGAAGCTGGGTACCAGCTGCTAGCAAGCTAGCTTGC
              *********************
gg_chr2+      ACATGCTTTTAAGAGCATAGAAACTGTAACTTGTTAGTTTTGCTGGCCAAAGAAGTTTCTCCTTAAAAG
                        ***********************************************************
3´ end        AAAAAAAAAAAAGAGCATAGAAACTGTAACTTGTTAGTTTTGCTGGCCAAAGAAGTTTCTCCTTAAAAG
```

pWLE25.2a

```
5´ end        TTTGTTTGACCAACAGAACACAATTTGTTAGATCTCTAGAAGCTGGGTACCAGCTGCTAG
              *****************************
gg_chr20-     TTGTTTGACCAACAGAACACAATTTGTTTAAGCAAGAAACAACCTGGGTTTTGCTACATG
                           ***********************************************
3´ end        AAAAAAAAAAAAAAGAACACAATTTGTTTAAGCAAGAAACAACCTGGGTTTTGCTACATG
```

pWLE25.3a

```
5´ end        GAATATCCACAATTCTACAGCTCCCAATGAAATGCATTTGGAGATCTCTAGAAGCTGGGTACCAGCTGCTAGCAAGCTAGC
              ****************************************
gg_chr1+      GAATATCCACAATTCTACAGCTCCCAATGAAATGCATTTGTTTCAGGGTGTGTTTGGATCTGAGAGACAGGATGTCTCAAG
                                       ********************************************************
3´ end        AAAAAAAAAAAAAAAAAAAAAAAAAAATGAAATGCATTTGTTTCAGGGTGTGTTTGGATCTGAGAGACAGGATGTCTCAAG
```

pWLE25.4a

```
5´ end        CCAAGAAAAGACATGGCGCAGATCTCTAGAAGCTGGGTACCAGCTGCTAGCAAGCTAGCTTGCTAGC
              *****************
gg_chr1-      CCAAGAAAAGACATGGCAGCTTTTCTTTTTTATGGTCAAAAGCTGACTGAAATCAATATTTAGTCTA
                *************************************** *************************
3´ end        AAAAGAAAAGACATGGCAGCTTTTCTTTTTTATGGTCAAAACCTGACTGAAATCAATATTTAGTCTA
```

pWLE27.7a

```
5´ end        AGTGCTAAAAAGAAAAAAGAAAAAAAAAACGAGATCTCTAGAAGCTGGGTACCAGCTGCTAGCAA
              ******************
gg_chr4-      AGTGCTAAAAAGAAAAAAAAAAAAAACAAACAACCCCACAAAACAGTAACAACAAAATCTAACTC
                                         **************************************
3´ end        AAAAAAAAAAAAAAAAAAAAGAAAAAAAAACAACCCCACAAAACAGTAACAACAAAATCTAACTC
```

pWLE28.3a

```
5´ end        ACTTTTTGTGAAGCTTTCAGCACCCGATCTGCAGCCAGGAGCCCTGATGTCGAGATCTCTAGAAGCTGGGTACCAGCTGCTAGCAAGCTA
                       **************** *************************
gg_chr 4 +    GAAAAGGGAGAAGCTTTCAGCACCCAATCTGCAGCCAGGAGCCCTGATGTCAGAATAGATTTTTGCCCAAGGCTCATGGAGGGTTTGCTA
                                                            4983796
                                         5016533
gg_chr4 +     GGGGATGCATCAAATTTGCAAGATAGTAAAAACAATGGAGAGAAGCGATGCTTATGAGTGGCTCTGTTCAGGCTTATTTTTCCACAAGGG
                                         ***************************************************************
3´ end        AAAAAAAAAAAAAAAAAAAAAAAAAAAAAAAACAATGGAGAGAAGCGATGCTTATGAGTGGCTCTGTTCAGGCTTATTTTTCCACAAGGG
```

pWLE28.5a

```
5´ end        TTACACTGCATCTGACATCTTAGACTCAGCACTAATCAGAACTTCATGTGTCTTAGTGGGTGCAGCGCACCAGCATGGCACATGTATAC
              ****************************************************
gg_chr20 -    TTACACTGCATCTGACATCTTAGACTCAGCACTAATCAGAACTTCATGTGTCAATACAGAACAAATACTGTGGTTTTTTTCCCCCTTTT
                                                   ****************************************************
3´ end        AAAAAAAAAAAAAAAAAAAAAAAAAAAAAAAAAAAAAAGAACTTCATGTGTCAATACAGAACAAATACTGTGGTTTTTTTCCCCCTTTT
```

pWLE28.8a

```
5´ end        AGAAGAGAGAGCGACGAAGAAAAATCCCTATAAAATTCCTCCTGGAAGGAACTGGTACCATTCCTTCTGAAACTATTCCAATCAATA
              *************************************** ******
gg_chr1 +     AGAAGAGAGAGCGACGAAGAAAAATCCCTATAAAATTCCCCCTGGAGAACTTATAATTTGGGAAAAAAGAAAAAGAAGTTTGTATTC
                                             ******** ***********************************************
3´ end        AAAAAAAAAAAAAAAAAAAAAAAAAAAAAAAAAAATTCCTCCTGGAGAACTTATAATTTGGGAAAAAAGAAAAAGAAGTTTGTATTC
```

pWLE30.5a

```
5´ end        CTTGTGAAAAGGTTTAATACTTCTCTTTTAAAAAACAAAATGCGAGATCTCTAGAAGCTGGGTACCAGCTGCTAGCAAGCTAGCTTGC
              *******************************************
gg_chr2 -     CTTGTGAAAAGGTTTAATACTTCTCTTTTAAAAAACAAAATGCTTTACTCTGAGTTTTGACAAGTCTGTACAGTAGTGGAATCTGCCC
                                          ************************************************************
3´ end        AAAAAAAAAAAAAAAAAAAAAAAAAAAATAAAAAACAAAATGCTTTACTCTGAGTTTTGACAAGTCTGTACAGTAGTGGAATCTGCCC
```

pWLE34.2a

```
5´ end        CTGGACAGGCTGGAGAAGTGGGCCCATGAAAGCCTAATGAGGAAAATTCAACAACCCTTCATGCTAAAAACTCTCAATAAATTAGGT
              ******************************************
gg_chr1 +     CTGGACAGGCTGGAGAAGTGGGCCCATGAAAGCCTAATGAGGTTCAATAAGGCCAAGTGCAGGGTACTGCACTTGGGTCGGGGCAAT
                                         ************************************************************
3´ end        AAAAAAAAAAAAAAAAAAAAAAAAAAAGAAAGCCTAATGAGGTTCAATAAGGCCAAGTGCAGGGTACTGCACTTGGGTCGGGGCAAT
```

pWLE34.3a

```
5´ end        TGTTGGAAGTTCTGGCCAGGGCAATCAGGCAGGAGAAGGAAATAAAGGGTATTCAATTAGGAAAAGAGGAAGTCAAATTGTCC
              ****************************************************
gg_chr3 -     GGTGGTCCCTTGAAAGCACTGTCAGGCATGTAGTTAGAAAGAGTCATACTATATCTCAGACTTTCAGATAAATCTTCAGTCTT
                                                 ************************************************
3´ end        AAAAAAAAAAAAAAAAAAAAAAAAAAAAAAAAAAAAGAAAGAGTCATACTATATCTCAGACTTTCAGATAAATCTTCAGTCTT
```

pWLE34.4a

```
5´ end       AAGCTGTTATTCAGTATCTTCAGTTGCTTTGAAAAGCAATGTTAGTGGATGAAATTGGAAACCATCATTCTCAGTAA
             *********************************************
gg_chr1+     AAGCTGTTATTCAGTATCTTCAGTTGCTTTGAAAAGCAATGTTAGATGCGCCAATATTCCACAAGCACTACTTTCTC
                                           ***********************************************
3´ end       AAAAAAAAAAAAAAAAAAAAAAAAAAAAAAGAAAAGCAATGTTAGATGCGCCAATATTCCACAAGCACTACTTTCTC
```

pWLE34.5a

```
5´ end       TCAATATCAATATAACAGTAGGTTGAAAGAACAAGAGATCTCTAGAAGCTGGGTACCAGCTGCTAGCAAGC
             **********************************
gg_chr5+     TCAATATCAATATAACAGTAGGTTGAAAGAACAAAAACTGGACTAGTGAAGATTTGCCAGTTTGGTGTATT
                                     ***********************************************
3´ end       AAAAAAAAAAAAAAAAAAAAAAAAGAAAGAACAAAAACTGGACTAGTGAAGATTTGCCAGTTTGGTGTATT
```

pWLE34.7a

```
5´ end       AAAACTATCTTAATTTCCTTAAAAACACAACACAGATCTCTAGAAGCTGGGTACCAGCTGCTAGCAAG
             ***********************************
gg_chr1 -    AAAACTATCTTAATTTCCTTAAAAACACAACACAGCTCTTGAAACTAGAAGAACACTTTGGTAAGAAA
                                 ************************************************
3´ end       AAAAAAAAAAAAAAAAAAAAAAAAACACAACACAGCTCTTGAAACTAGAAGAACACTTTGGTAAGAAA
```

pWLE35.1a

```
5´ end       GCATTTCACAATGTTTATTAAATCTATACACATATTATCCAGGAGAACTTCCCCAATATAGCAA
             ********** ********************
gg_chr1 +    GCATTTCACAGTGTTTATTAAATCTATACACAAGAAAGTTTGAAGTAGATGTTATTTCATCGTG
                                *********************************************
3´ end       AAAAAAAAAAAAAAAAAAAAAATCTATACACAAGAAAGTTTGAAGTAGATGTTATTTCATCGTG
```

pWLE35.2a

```
5´ end     AAGTTGCCTACTGAGCATCTGTGCTAAAAATGGTGAAGTATCATGTATACATATGTAACTAACCCGATCCGAACAA
           *****************************************
gg_chr1 -  AAGTTGCCTACTGAGCATCTGTGCTAAAAATGGTGAAGTATGAGAGACTGTGTATCCATGAAGTCACACTATATTG
                                   ****************************************************
3´ end     AAAAAAAAAAAAAAAAAAAAAAAATAAAAATGGTGAAGTATGAGAGACTGTGTATCCATGAAGTCACACTATATTG
```

pWLE35.3a

```
5´ end     AAGCCTCACTTCCTAAAATCAACAGCATTTTGGAGATCTCTAGAAGCTGGGTACCAGCTGCTAGCAAGCTAGCTTGCTAGCGGCCGCGGGGG
           ********************************
gg_chr4 -  AAGCCTCACTTCCTAAAATCAACAGCATTTTGATCCA-TTTTAGAGCACAGAAC---TTAAGACTACATTAGAACACAATAAAAACACTTTA
                               *****************-**********-*****---***********************************
3´ end     AAAAAAAAAAAAAAAAAAAAAACAGCATTTTGATCCATTTTTAGAGCATAGAACTTATTAAGACTACATTAGAACACAATAAAAACACTTTA
```

pWLE35.5a

```
5´ end     AAAAACATAGGGAAGATTTATTGAATTATGAAAATAAATCGAGATCTCTAGAAGCTGGGTACCAGCTGCTAGCAAG
           ****************************************
gg_chrZ +  AAAAACATAGGGAAGATTTATTGAATTATGAAAATAAATCCAGGACCACAGGCAGGACTTTATTGCCAGCCTGGAA
                                        ***********************************************
3´ end     AAAAAAAAAAAAAAAAAAAAAAAAAAAAAGAAAATAAATCCAGGACCACAGGCAGGACTTTATTGCCAGCCTGGAA
```

pWLE35.7a

```
5´ end     AGAGCAGAGCATCCTGCTCAGGGAGTAGAAAGCCAGTGCCTGAAGGACTTCATGTCCAAAACACCAAAAGCAATG
           ******************************************
gg_chr4 -  AGAGCAGAGCATCCTGCTCAGGGAGTAGAAAGCCAGTGCCTGGCTCCGTGATGAACAGCTCTGAAGGAAGGAGCA
                                     *************************************************
3´ end     AAAAAAAAAAAAAAAAAAAAAAAAAAAGAAAGCCAGTGCCTGGCTCCGTGATGAACAGCTCTGAAGGAAGGAGCA
```

```
pWLE35.8a
5´ end       AACTTTGGTTTGTTCATCTCCCTTAAAAAACATATATTTGACACATAGGCTCAAAATAAAAGGATGGAGGAAGATCTACCAAG
             *****************************************
gg_chr2 -    AACTTTGGTTTGTTCATCTCCCTTAAAAAACATATATTTGAGTTATTAATATTTTAGATTTATTCACTGCTTTCATTTCCAAC
                                     ***********************************************************
3´ end       AAAAAAAAAAAAAAAAAAAAAAAAAAAAAACATATATTTGAGTTATTAATATTTTAGATTTATTCACTGCTTTCATTTCCAAC

pWLE35.9a
5´ end       GCTAGCCATGTATTTGGTCTTGCTTGAAAGAGAAGAAATGCAGAGATCTCTAGAAGCTGGGTACCAGCTGCTAGCAAGCTAGCTTGC
             ******************************************* *******
gg_chr1 -    GCTAGCCATGTATTTGGTCTTGCTTGAAAGAGAAGAAATGCAGTGATCTCTTTTCACTCTGGTGTCAGTCTCAAATATTTCTAGCTA
                                             *******************************************************
3´ end       AAAAAAAAAAAAAAAAAAAAAAAAAAAAAAAAAAGAAATGCAGTGATCTCTTTTCACTCTGGTGTCAGTCTCAAATATTTCTAGCTA

pWLE36.1a
5´ end       GTCTAATTAAAAGGCTGAACAGTTTCAGAAAGAATGGTTAAGATCTCTAGAAGCTGGGTACCAGCTGCTAGCAAGCT
             ****************************************
g_chr1 -     GTCTAATTAAAAGGCTGAACAGTTTCAGAAAGAATGGTTAGCTTCTGAGAAAGTGATATTCTAAATCAGAAAAGCAG
                                         *************************************************
3´ end       AAAAAAAAAAAAAAAAAAAAAAAAAAAAAAAGAATGGTTAGCTTCTGAGAAAGTGATATTCTAAATCAGAAAAGCAG

pWLE36.2a
5´ end       GCATGTGCTCAGGTATACAGCTGACAACTTCTTAAGAGAAAGCCAGATCTCTAGAAGCTGGGTACCAGCTGCTAGCAAG
             **********************************************
gg_chr12 -   GCATGTGCTCAGGTATACAGCTGACAACTTCTTAAGAGAAAGCCAGCACTGGAAAAGGGGAAACTCAACTATGGATTTT
                                              **********************************************
3´ end       AAAAAAAAAAAAAAAAAAAAAAAAAAAAAAAAAAAGAGAAAGCCAGCACTGGAAAAGGGGAAACTCAACTATGGATTTT
```

pWLE36. 5a

```
5´ end          TATTTTCCTTTCACAAAGTGTGCAGGTCAAGAAATATATAGTAGATCTCTAGAAGCTG
                ************** ******** *******************
gg_chr5 –       TATTTTCCTTTCACGAAGTGTGCGGGTCAAGAAATATATAGTATGCCCAACACCTTAT
                                            ******************************
3´ end          AAAAAAAAAAAAAAAAAAAAAAAAAAAAAAGAAATATATAGTATGCCCAACACCTTAT
```

pWLE36. 6a

```
5´ end          TTGCTACCCGTCAAGAGTCAATATGCCCAAAACGCAGCCAGAGAGAAAGGTCGGGTTACCCTCAAAGGAAAGCCCA
                **********************************
gg_chr2 +       TTGCTACCCGTCAAGAGTCAATATGCCCAAAACGGACTACAGCACCCTTATCCTTTCCATCTCCCCCCAGAAAAGA
                                   *********************************************************
3´ end          AAAAAAAAAAAAAAAAAAAAATATGCCCAAAACGGACTACAGCACCCTTATCCTTTCCATCTCCCCCCAGAAAAGA
```

pWLE36. 7a

```
5´ end          CTGTAGTTCTGTGACAATAGTTGTGAAAGTACTCTGTGGACGTATGTTTATTGCGGCACTATTCACAATAGCAA
                ****************************************
gg_chr11 +      CTGTAGTTCTGTGACAATAGTTGTGAAAGTACTCTGTGGAAGTGGTCTTAAACACCAGCAAACACTTTGAGTCA
                                        **************************************************
3´ end          AAAAAAAAAAAAAAAAAAAAAAAAGAAAGTACTCTGTGGAAGTGGTCTTAAACACCAGCAAACACTTTGAGTCA
```

pWLE36. 8a

```
5´ end          GCTCTATCAGAGTTGAAGCAATTAAAAAACCAAGTATAACCTGGCAAACCGAATCCAGCAGCACATCAAAAAACTTATCCA
                ****************************************
gg_chr7 +       GCTCTATCAGAGTTGAAGCAATTAAAAAACCAAGTATAACATAACCAGTTGAATATATGTAGCATACATTGCTCTATTCTT
                                       **********************************************************
3´ end          AAAAAAAAAAAAAAAAAAAAAAAAAAAAACCAAGTATAACATAACCAGTTGAATATATGTAGCATACATTGCTCTATTCTT
```

pWLE36. 9a

```
5´ end       GACCCAGATTTCAGTTCCTGTTAATAAGAAGTTCAAACTCTAAAACGCAGAGCGCCTCTCCTCCTC
             *************************************
gg_chr2 +    GACCCAGATTTCAGTTCCTGTTAATAAGAAGTTCAAAGCAGTAGATTATAAAGTCTTTACCTACCA
                                      *****************************************
3´ end       AAAAAAAAAAAAAAAAAAAAAAAAAAAGAAGTTCAAAGCAGTAGATTATAAAGTCTTTACCTACCA
```

pWLE36. 10a

```
5´ end       GTAGGTTTGCCTTGTATAAGGCATGATCAAGAACTAAGGCTCAAGATCTCTAGAAGCTGGGTACCAGCTGCTAGCAAGCTAGCT
             ********************************************
gg_chr1 -    GTAGGTTTGCCTTGTATAAGGCATGATCAAGAACTAAGGCTCAAGCTTGGTTGTGGTTAGGGGTTACTTTCTTAGATAAAAATC
                                         ********************
3´ end       AAAAAAAAAAAAAAAAAAAAAAAAAAAAAAGAACTAAGGCTCAAGCTTTAAGACCTTTGCTGAGCAAGAAGGTGAATGGTCACA
```

pWLE36. 12a

```
5´ end       GGGATCAGTAACCAAAACACAACTCCTCACCAAAAACAATATGATCGAGATCTCTAGAAGCTGGGTACCAGCTGCTAGCAAGCTAGCTTGCTA
             *********************************** **********
gg_chr1 -    GGGATCAGTAACCAAAACACAACTCCTCACCAAAACCAATATGATCTTGTTGTAGAAGATACTAAGTGTGCACCTCACTGTGTAAAACTGAGC
                                            **** *********************** ****  ************ **************
3´ end       AAAAAAAAAAAAAAAAAAAAAAAAAAAAAAAAAAAACAATATGATCTTGTTGTAGAAGACACTAGGGTGTGCACCTCATTGTGTAAAACTGAG
```

pWLE36. 13a

```
5´ end       CCAAAGTAGCCAGAAGTCTGCTAGTTTCCTTAGAACAGCAGAGGGGAGAGATCTCTAGAAGCTGGGTACCAGCTGCTA
             ***********************************************
gg_chr1 +    CCAAAGTAGCCAGAAGTCTGCTAGTTTCCTTAGAACAGCAGAGGGGAACAGTCAGATTTAACAGTATTAACGTCTCAT
                                            ***********************************************
3´ end       AAAAAAAAAAAAAAAAAAAAAAAAAAAAAAAAGAACAGCAGAGGGGAACAGTCAGATTTAACAGTATTAACGTCTCAT
```

pWLE36.14a

```
5´ end       AAAGGTGAAAATCTGGTATAAATCAACTTGAAAACCAGTCTGGAGATCTCTAGAAGCTGGGTACCAGCTGCTAGCAAGCTAGCT
             ******************************************
gg_chr1 +    AAAGGTGAAAATCTGGTATAAATCAACTTGAAAACCAGTCTGTTTCATTTTGAAAGGAAAAAAAAATGCAGGTCTATGTGTCAG
                                          *******************************************************
3´ end       AAAAAAAAAAAAAAAAAAAAAAAAAAAAAGAAAACCAGTCTGTTTCATTTTGAAAGGAAAAAAAAATGCAGGTCTATGTGTCAG
```

pWLE36.16a

```
5´ end       AAATGATTTTATGATTCTATGACAAATTAAGAAAATAATAATAATCCGAGATCTCTAGAAGCTGGGTACCAGCTGCTAGC
             ***********************************************
gg_chr2 +    AAATGATTTTATGATTCTATGACAAATTAAGAAAATAATAATAATCCATAAATTGCCACTCATCAAGCAAAGGATGCTTA
                                         ****************************************************
3´ end       AAAAAAAAAAAAAAAAAAAAAAAAAAAAAAGAAAATAATAATAATCCATAAATTGCCACTCATCAAGCAAAGGATGCTTA
```

pWLE36.20a

```
5´ end       CAATTCAGTTGAAGATTTCTAGTCAATTTAAGATCTTTTAGAGATCTCTAGAAGCTGGGTACCAGCTGCTAGCAAGC
             *****************************************
gg_chr2 -    CAATTCAGTTGAAGATTTCTAGTCAATTTAAGATCTTTTAGCATTATTTTTTCACTTTACAATTATAATCTGTTCAC
                                          ************************************************
3´ end       AAAAAAAAAAAAAAAAAAAAAAAAAAAAAAAGATCTTTTAGCATTATTTTTTCACTTTACAATTATAATCTGTTCAC
```

L1.3 / DT40 Ku70-/- cells

p7LE13. 1a

```
5´ end      ACTGCTTCGTCTAGTAGTTTCTTTATATGTTGTTAAAAATGTATAATGTTGACCCAGCCATCCCATTACTG
            *************************************************
gg_chr4-    ACTGCTTCGTCTAGTAGTTTCTTTATATGTTGTTAAAAATGTATAATGTATTTATTCAAGATAAAATACAT
                                              *************************************
3´ end      AAAAAAAAAAAAAAAAAAAAAAAAAAAAAAAAAAAAAAATGTATAATGTATTTATTCAAGATAAAATACAT
```

p7LE13. 2a

```
5´ end      GATAGAAAAAGTAATTATAGGATCTCTAGAAGCTGGGTACCAGCTGCTAGCAAGCTAGCTTGCTAGCGGCCGCGGGG
            *******************
gg_chr2+    GATAGAAAAAGTAATTATATCTCTCACTGTATTCATGCTGCAGTGCTTCACTGATGCTGGTCTCAGTCCCTGTATGT
               ***********************************************
3´ end      AAAAGAAAAAGTAATTATATCTCTCACTGTATTCATGCTGCAGTGCTTCA
```

p7LE13. 3a

```
5´ end      CTGTCAAAATAACGAATTTACGAGATCTCTAGAAGCTGGGTACCAGCTGCTAGCAAGC
            *********************
gg_chr18-   CTGTCAAAATAACGAATTTACATCATAAATGCACGGGCTCCCTTTTTAAAGGCCTCTT
                 *****************************************************
3´ end      AAAAAAAAATAACGAATTTACATCATAAATGCACGGGCTCCCTTTTTAAAGGCCTCTT
```

p7LE13. 5a

```
5´ end      CTCATTATTTTAGTTTTGCATACGCTTACAGTCAAAAAAATATAAAAAGCAGATCTCTAGAAGCTGGGTACCAGCTGCTAGCAAGCTAGCT
            ***************************************************
gg_chr2-    CTCATTATTTTAGTTTTGCATACGCTTACAGTCAAAAAAATATAAAAAGCATTTCATTTGTAACACTAACCATTTGTGTTGCGTGAAAACC
                                             **********************************************************
3´ end      AAAAAAAAAAAAAAAAAAAAAAAAAAAAAAAAAAAAAAAATATAAAAAGCATTTCATTTGTAACACTAACCATTTGTGTTGCGTGAAAACC
```

p7LE13. 6a

```
5´ end      TGTTAATCTCAAAAGAGAAAATCTAACAGAATAAAAACCCAAAACCTTGCAGCGCACCAGCATGGCACATG
            *********************************************
gg_chr9 -   TGTTAATCTCAAAAGAGAAAATCTAACAGAATAAAAACCCAAAACTTTGTAGAGAGCGTGTATATTGTAAA
                                            ***************************************
3´ end      AAAAAAAAAAAAAAAAAAAAAAAAAAAAAAAAAAAAACCCAAAACTTTGTAGAGAGCGTGTATATTGTAAA
```

p7LE14. 1a

```
5´ end      CTAGCCCAGGAGATCTGACAGAGACATATAGAAATTAGGTAGCTGTACAAGGCTACAGTAACCAAAACAGCATGGT
            *********************************************
gg_chr8 +   CTAGCCCAGGAGATCTGACAGAGACATATAGAAATTAGGTAGCTGGAAACTGTGATCAAGCTGCTAAGAAGTTGAA
                                         ********************************
3´ end      AAAAAAAAAAAAAAAAAAAAAAAAAAAAAAGAAATTAGGTAGCTGGAAACTGTGATCAAGC
```

p7LE14. 2a

```
5´ end      ACATAAAACAATAGAAGGACAGTCTTGTCAAAACAACTTGATTTCAGCAAAGTCTCAGGATACAAAATCAATGTACAAAA
            ********************************************
gg_chr10-   ACATAAAACAATAGAAGGACAGTCTTGTCAAAACAACTTGATTTCACATTTTGATGACAGGTGTGGCTGGGGATTTATTC
                                         ***************************************************
3´ end      AAAAAAAAAAAAAAAAAAAAAAAAAAAAAAAAACAACTTGATTTCACATTTTGATGACAGGTGTGGCTGGGGATTTATTC
```

p7LE14. 3a

```
5´ end      ACAAGATGCCAATTCAGGTATGGCCATAACAATGTGCATTTCATCCTCAATAAAATACTGGCAAACCGAATCCA
            *******************************************
gg_chr12-   ACAAGATGCCAATTCAGGTATGGCCATAACAATGTGCATTTCAACTGAGATAATAATCAATGTTACCTTTAAGT
                                       ***********************************************
3´ end      AAAAAAAAAAAAAAAAAAAAAAAAAAAAACAATGTGCATTTCAACTGAGATAATAATCAATGTTACCTTTAAGT
```

p7LE14. 4a

```
5´ end          ATCAATTAATAACATAAAGGGTTGATGAAAGTAACTGAGAGATCTCTAGAAGCTGGGTACCAGCTGCTAGCAAG
                ****************************************
gg_chr11-       ATCAATTAATAACATAAAGGGTTGATGAAAGTAACTGAGATGCTTATGTGATTTGTGTTAATTTTTTATAAAAT
                                          ************************************************
3´ end          AAAAAAAAAAAAAAAAAAAAAAAAAAGAAAGTAACTGAGATGCTTATGTGATTTGTGTTAATTTTTTATAAAAT
```

p7LE14. 5a

```
5´ end          CACATAAACACAACAATTTCATAGCTCTCCAAAAATCAAATGAACCTATGGAACCAAAAAA
                ************************* ************ *******
gg_chr 3 +      CACATAAACACAACAATTTCATAGCGCTCCAAAAATCAGATGAACCAAAGAGATGTACTGC
                                              ******** **********************
3´ end          AAAAAAAAAAAAAAAAAAAAAAAAAAAAAAAAAAATCAAATGAACCAAAGAGATGTACTGC
```

p7LE14. 6a

```
5´ end          TTAGGAGAGAATTTGTATTCTGGAATCAAAAGACTAGAATCCAGAACTAGAAATACCATTTGACC
                *******************************************
gg_chr 9 -      TTAGGAGAGAATTTGTATTCTGGAATCAAAAGACTAGAATCCATTTAAGAGCTTGGGATGAAGAC
                                           **************************************
3´ end          AAAAAAAAAAAAAAAAAAAAAAAAAAAAAAAGACTAGAATCCATTTAAGAGCTTGGGATGAAGAC
```

p7LE15. 1a

```
5´ end          CCACCAACAAATACAGAGAAACATATTCCATTTATGAAAAAGCTGTGGATGCTCAAGGAAATAAAAGAGGACACAAACAAATGGAAG
                *************************************************
gg_chr2-        CCACCAACAAATACAGAGAAACATATTCCATTTATGAAAAAGCTGTGGAAAATTTGAAAAATCTTCTCAAAAACAGTAGCATTGTAT
                                                   ****************************************************
3´ end          AAAAAAAAAAAAAAAAAAAAAAAAAAAAAAAAAAAGAAAAAGCTGTGGAAAATTTGAAAAATCTTCTCAAAAACAGTAGCATTGTAT
```

p7LE15. 2a

```
5´ end     TCCAGAGGTGCTCTCTGTTGATTATGGCCTTGAAAAATGACAGATAGTCTTTTATTGCCGATCCCCTCAGAAGAACTCGTCAAGAAGG
           **********************************************
gg_chr12+  TCCAGAGGTGCTCTCTGTTGATTATGGCCTTGAAAAATGACAGATAAATTTTAGTGTTTCAGGAATTGCTGCTGGCTGTTACTCAGTCT
                                          **********************************************************
3´ end     AAAAAAAAAAAAAAAAAAAAAAAAAAAAAAAGAAAAATGACAGATAAATTTTAGTGTTTCAGGAATTGCTGCTGGCTGTTACTCAGTCT
```

p7LE15. 3a

```
5´ end     TGTTTGATTGATCAAATTCTCAGCTACAAAAATCTATTTTTTACTTTATAGACAAGCAAATGTTGAGAGATTTTGTCACCACCAGGCC
           *****************************************
gg_chr2-   TGTTTGATTGATCAAATTCTCAGCTACAAAAATCTATTTTTAACATAGTTACCACCACTAGCTATGCATTTTTGCCAGTGATGAACAA
                                      *************************************************************
3´ end     AAAAAAAAAAAAAAAAAAAAAAAAAAAAAAAATCTATTTTTAACATAGTTACCACCACTAGCTATGCATTTTTGCCAGTGATGAACAA
```

p7LE15. 4a

```
5´ end     GGTTAAAGTCCATTTCCAGAAAAGTGTCTAGACTTGATTTAAAAACTTCCAGAAGGGGAATATCACACTCTGGGGACTGTGGTGGGGTCGGGGGAGGGGGG
           *****************************************************
gg_chr1+   GGTTAAAGTCCATTTCCAGAAAAGTGTCTAGACTTGATTTAAAAACTTCCAGAGAAAAGAATTTGCTGTTTCCTGTATAAGTCTATTCCAATTCATAATGG
                                                   *************************************************************
3´ end     AAAAAAAAAAAAAAAAAAAAAAAAAAAAAAAAAAAAAAAAAAAAACTTCCAGAGAAAAGAATTTGCTGTTTCCTGTATAAGTCTATTCCAATTCATAATGG
```

p7LE15. 5a

```
5´ end     GCAGAAAATCCCCTGTACATTCAGACAAGTAACAAGAAATCACAGATCTCTAGAAGCTGGGTACCAGC
           ********************************************
gg_chr4-   GCAGAAAATCCCCTGTACATTCAGACAAGTAACAAGAAATCACATGCACTGTAAATGACAGTAAATTC
                                            ***********************************
3´ end     AAAAAAAAAAAAAAAAAAAAAAAAAAAAAAAAAAAGAAATCACATGCACTGTAAATGACAGTAAATTC
```

p7LE15.6a

```
5´ end       GCATGTAATTTGGGTTAAGAAAAATAAGCGAGAAACAAACGACCCAACACCCGTGCGTTTTATTCTG
             ***********************************
gg_chr7-     GCATGTAATTTGGGTTAAGAAAAATAAGCGAGAAATAATATTTTGATGCATCTCCTGTCAGTGTGTA
                             ***************************************************
3´ end        AAAAAAAAAAAAAAAAAAGAAAAATAAGCGAGAAATAATATTTTGATGCATCTCCTGTCAGTGTGTA
```

p7LE16.1a

```
5´ end       GGGTTTACCATTAAGTGGAGAAGTAACAGCAAAAGATGATTTCAATAAAAGAGGACACAAACAAATGGAAGAACATTCCATG
             *********************************************
gg_chr 2 -   GGGTTTACCATTAAGTGGAGAAGTAACAGCAAAAGATGATTTCAAGACCAAATTTCTAATAGAACTCTCCTTTTCCCAGAGG
                                           ****************************************************
3´ end       AAAAAAAAAAAAAAAAAAAAAAAAAAAAAAAAAAGATGATTTCAAGACCAAATTTCTAATAGAACTCTCCTTTTCCCAGAGG
```

p7LE16.2a

```
5´ end       TTTAGACAAACTTGGTCAATAAAAAGTTTTTAAAAATATTCCAGAGATCTCTAGAAGCTGGGTACCAGCTGCTAGC
             *******************************************
gg_chr 5 +   TTTAGACAAACTTGGTCAATAAAAAGTTTTTAAAAATATTCCAAAGGATTAGGATTTACCTGCTGAAAACATGCTT
                                            *********************************************
3´ end       AAAAAAAAAAAAAAAAAAAAAAAAAAAAAAAAAAAATATTCCAAAGGATTAGGATTTACCTGCTGAAAACATGCTT
```

p7LE16.4a

```
5´ end       GACAAGAGCTCTGCATTATTATTTGGCAGTAAAAAGTATTTCCGTGGGGTCGGGGGAGGGGGGAGGGATAGCATTGGGAGATAT
             *******************************************
gg_chr6+     GACAAGAGCTCTGCATTATTATTTGGCAGTAAAAAGTATTTCCATAGCTGAAAAGGCTTTACTTGCTGGGTCTGTGTTCCTATT
                                           ******************************************************
3´ end       AAAAAAAAAAAAAAAAAAAAAAAAAAAAAAAAAAAGTATTTCCATAGCTGAAAAGGCTTTACTTGCTGGGTCTGTGTTCCTATT
```

p7LE18. 2a

```
5´ end       TTTCACCGATATCCCTAAGTAACAGCTAGTTTAAGAACAACAGAGATCTCTAGAAGCTGGGTACCAG
             ******************************************
gg_chr 2 -   TTTCACCGATATCCCTAAGTAACAGCTAGTTTAAGAACAACAACAACAAAAGCAAGAAGAAAATCCC
                                             ***********************************
3´ end       AAAAAAAAAAAAAAAAAAAAAAAAAAAAAAAAAAGAACAACAACAACAAAAGCAAGAAGAAAATCCC
```

p7LE18. 6a

```
5´ end       CCCTTAATTTGCAATGCTAAAATTAATTAGATAAGATCTCTAGAAGCTGGGTACCAGCTGCTAG
             ********************************
gg_chr2 -    CCCTTAATTTGCAATGCTAAAATTAATTAGATGAACAGTAAAATCCCCATGGAATTTTCATATG
                              ***********************************************
3´ end       AAAAAAAAAAAAAAAAATAAAATTAATTAGATGAACAGTAAAATCCCCATGGAATTTTCATATG
```

p7LE19. 1a

```
5´ end       TAATTTTTTAGCAAATGGTTTCATTGTGTAAGGACAGGTAGAGATCTCTAGAAGCTGGGTACCAGCTGCTAGCAAGCTAGC
             ****************************************
gg_chr1 +    TAATTTTTTAGCAAATGGTTTCATTGTGTAAGGACAGGTAATCTCAGCGACACATGTCAGCTGTCTCCTGCTCTCCGCTGA
                                          ***************************************************
3´ end       AAAAAAAAAAAAAAAAAAAAAAAAAAAAAAAGGACAGGTAATCTCAGCGACACATGTCAGCTGTCTCCTGCTCTCCGCTGA
```

p7LE19. 3a

```
5´ end       CTTCTGATTTGCAGTGTTTAAAATAAAGGGCAACAGACCGATCCCCTCAGAAGAACTCGTCAAG
             **************************************
gg_chr3 -    CTTCTGATTTGCAGTGTTTAAAATAAAGGGCAACAGACACTTGGATTCAGATAACCCAGCACAG
                               **********************************************
3´ end       AAAAAAAAAAAAAAAAAATAAAATAAAGGGCAACAGACACTTGGATTCAGATAACCCAGCACAG
```

p7LE19.6a

```
5´ end       AATAGTGGGTTTTCTAGTGACATGAAAAGCATCTCTCACGGGCAGACAGACTGCCTCCTCA
             *************************************
gg_chr3 +    AATAGTGGGTTTTCTAGTGACATGAAAAGCATCTCTCTACTCTAGCTGTAAAATCAGGTAC
                                    **************************************
3´ end       AAAAAAAAAAAAAAAAAAAAAAAGAAAAGCATCTCTCTACTCTAGCTGTAAAATCAGGTAC
```

L1.3 / HeLa-RC cells

pHLE1.2a

```
5´ end      TTGTAAATATGCCACCTTTATATTTATAAGAGTATTTACGAGATCTCTAGAAGCTGGGTACCAGCTGCTAGCAAGCTAGCTTGCTAG
            ***************************************
hs_chr4 −   TTGTAAATATGCCACCTTTATATTTATAAGAGTATTTACACAAGAATTTGCAGTTTTAAGTCGTTATCAATCATTGAGTTGATATAA
                                       ************************************************************
3´ end      AAAAAAAAAAAAAAAAAAAAAAAAAAAAAGAGTATTTACACAAGAATTTGCAGTTTTAAGTCGTTATCAATCATTGAGTTGATATAA
```

pHLE1.4a

```
5´ end      TCCATTTTGTCCAAAAAGCAAAACAAAACGAACTCAAGTTAAAAAAAAAAAAGTCTGGATTAAAGATTTAAACGTTAAACCTAAAACCATAA
            *******************************************************
hs_chr2+    TCCATTTTGTCCAAAAAGCAAAACAAAACGAACTCAAGTTAAAAAAAAAAAAGTCAAATTTATAAAAGGAGAACACCAAATATTATTTCATG
                                                    ****************************************************
3´ end      GCTGCAATAAACAAGTTAACAACAAAAAAAAAAAAAAAAAAAAAAAAAAAAAGTCAAATTTATAAAAGGAGAACACCAAATATTATTTCATG
```

pHLE1.5a

```
5´ end      TTTGCCAACAGGAGAGCAGTATCTACATTTAATTGAAAAAAAAACCAAACACCGCATATTCTCACTCATAGGTGGGAATTGAAC
            **************************************
hs_chr3 +   TTTGCCAACAGGAGAGCAGTATCTACATTTAATTGAAATAAACTACCATGTTATTTGTAGTATTCTCATAACAATTCTTGGAAG
                                                   *********************************************
3´ end      AAAAAAAAAAAAAAAAAAAAAAAAAAAAAAAAAAAAAAAAAACTACCATGTTATTTGTAGTATTCTCATAACAATTCTTGGAAG
```

pHLE1.6a

```
5´ end      GCCACTTCAGTTGTTTTGTTGAAAATACCTAATGCTAGATGACACATTAGTGGGTGCAGCGCACCAGCATGGCACATG
            ******************************
hs_chr17 +  GCTGTGCCACTTCAGTTGTTTTGTTGAAAACAAAACAACAAAACAAAACAAAAACAGATGCTCTGGACACTGTATTTC
                                                   ***************************************
3´ end      AAAAAAAAAAAAAAAAAAAAAAAAAAAAAAAAAAAAAAAAAAACAAAACAAAAACAGATGCTCTGGACACTGTATTTC
```

pHLE2. 3a

```
5´ end      GGCGCGGTGGCTCACGCCTGTGGTCAAAATGTGGCACATATACACCATGGAATACTATGCAGCCATAAAAAAT
            *********************
hs_chr2+    GGCGCGGTGGCTCACGCCTGTAATCCCAACACTTTGGGAGGCCAAGGTGGGTGGATCACAAGGTCAGGAGTTC

                                           116533960
hs_chr3 +   TTCTAATTATGATGTAATCCATGTCTCAATAAAAAAACACTCACATGCTTCCAGAGGTTAAAAATGGGAGGAA
                                          *******************************************
3´ end      AAAAAAAAAAAAAAAAAAAAAAAAAAAAAAAAAAAAACACTCACATGCTTCCAGAGGTTAAAAATGGGAGGAA

 (comment)
pLEmH       TCCAACAATGATAGACTGGATTAAGAAAATGTGGCACATATACACCATGGAATACTATGCAGCCATAAAAAAT
                                     ************************************************
5´ end      GGCGCGGTGGCTCACGCCTGTGGTCAAAATGTGGCACATATACACCATGGAATACTATGCAGCCATAAAAAAT
            *********************
hs_chr2+    GGCGCGGTGGCTCACGCCTGTAATCCCAACACTTTGGGAGGCCAAGGTGGGTGGATCACAAGGTCAGGAGTTC
                                ********
hs_chr3+    GGATGGAGGTTACAAGGAAATGGTCAAACTTGAATGAAACTAGCCAAATATCCTTTTGT
                                 116534108
```

pHLE2. 5a

```
5´ end      GTAAATTAATATAACCTTTCATCTTAAGGGAGGGAAAATGGAAGCTCGGGGGAGGGGGGAGGGATAGCATTGGGAGATATACCTAATGCTAGATGACA
            **********************************************
hs_chr2 –   GTAAATTAATATAACCTTTCATCTTAAGGGAGGGAAAATGGAAGCTAATTATTCAAAATTTAAAACGTGTATATCCTTTATTTTTAATTTGTACTATT
                                             *****************************************************************
3´ end      AAAAAAAAAAAAAAAAAAAAAAAAAAAAAAAAAGAAAATGGAAGCTAATTATTCAAAATTTAAAACGTGTATATCCTTTATTTTTAATTTGTACTATT
```

pHLE2. 9a

```
5´ end      ATAAAAATGTTAGTGTAAAACACATTCATTTTTCCAATGGCGAAACCAAACACCGCATATTCTCACTCATAGGTGGGAATTGAACAATGAGAT
            *******************************************
hs_chr4 –   ATAAAAATGTTAGTGTAAAACACATTCATTTTTCCAATGGCGATTCAAGATTCAGTAAAGATAAGGAAATAATTGCTTGTAATTCATTAGTAA
                                                          ***********************************************
3´ end      AAAAAAAAAAAAAAAAAAAAAAAAAAAAAAAAAAAAAAAAAAAAAAAAGATTCAGTAAAGATAAGGAAATAATTGCTTGTAATTCATTAGTAA
```

pHLE2. 10a

```
5´ end      GCATATATAGGTGGTAAAACTGTAAAGAAAAGCACATTGTTATAACAACAATGATAGACTGGATTAAGAAAATGTGGCACATATACACCATGGA
            *********************************************
hs_chr6 –   GCATATATAGGTGGTAAAACTGTAAAGAAAAGCACATTGTTATAATAAAAATCAGAATCATATATAGAGATGAGAACAGATCATTATCCGGGAG
                                                       ***************************************************
3´ end      AAAAAAAAAAAAAAAAAAAAAAAAAAAAAAAAAAAAAAAAAAAAATAAAAATCAGAATCATATATAGAGATGAGAACAGATCATTATCCGGGAG
```

pHLE2. 11a

```
5´ end      TATAATAATAATAAATCATGAAACTTGGCACTTTAGAATAAACTGGCTAGCCATATGTAGAAAGCTGAAACTGGACCCCTTCCTTACACC
            ********************** *****************
hs_chr12 +  TATAATAATAATAAATCATGAACCTTGGCACTTTAGAATAGCCTAAGACTTGAAGCTTATTTAGTAATGAGTTTGTATGTTAGTCAAGTG
                                              ************************
3´ end      AAAAAAAAAAAAAAAAAAAAAAAAAAAAAAAAAAAGAATAGCCTAAGACTTGAAGCTTGGCTTTGGCGTCAGGTAGTCTCAGCTCAAATT
```

pHLE3. 5a

```
5´ end      TTTGAGCTTATTTTAGTTACCCTTTTATCTTTTGAAAAGACATTATCATGCTGCTATAAAGACACATGCACACGTATGTTTATTGCGGCACTATT
            **********************************************
hs_chr5 +   TTTGAGCTTATTTTAGTTACCCTTTTATCTTTTGAAAAGACATTATTATTTTTATGTACATGTATATCTAATTCATTGCACAAATCCTTACCTCT
                                                          *************************************************
3´ end      AAAAAAAAAAAAAAAAAAAAAAAAAAAAAAAAAAAAAAAAAAAAAATATTTTTATGTACATGTATATCTAATTCATTGCACAAATCCTTACCTCT
```

pHLE3. 9a

```
5´ end      CGTGTTTTCCTTAAATTAGTCAATCTATAACCCGCAAAGGAAAATCTAAAAGAATCTACAATGAACTTAAACAAATTTACAAGAAAAAAACA
            *****************************************************
hs_chr7 -   CGTGTTTTCCTTAAATTAGTCAATCTATAACCCGCAAAGGAAAATCTAAAAGATAATGCTTGTGGACTTTAATGAAAGTCTAGTTCCGGCTG
                                                   *****************************************************
3´ end      AAAAAAAAAAAAAAAAAAAAAAAAAAAAAAAAAAAAAAAGAAAATCTAAAAGATAATGCTTGTGGACTTTAATGAAAGTCTAGTTCCGGCTG
```

pHLE4. 4a

```
5´ end      CCTGGCACTTAGAAGGGAAGAATCTTTGCAACACAAATGACGATTAAAGAGGGGGGAGGGATAGCATTGGGAGATATACCTAATGCTAGATGACACATT
            **************************************************
hs_chr15 +  CCTGGCACTTAGAAGGGAAGAATCTTTGCAACACAAATGACGATTAAAGAATAACAGTGAAAAGTCAGCAGACATTCCACAAGAGGGAAGTCATCTTCA
                                                         ******************************************************
3´ end      AAAAAAAAAAAAAAAAAAAAAAAAAAAAAAAAAAAAAAAAAAAAAAAAGAATAACAGTGAAAAGTCAGCAGACATTCCACAAGAGGGAAGTCATCTTCA
```

pHLE4. 6a

```
5´ end          CACTCCAGCCTGGGTGACAGAGCTAGACTCCATTTCAAAAAAAAAAAACGCCATTGCCCAGGCTTGCTTAGGTAAACAAAGCAGCCGGGAAGC
                ************************************************
hs_UCSC_chr5 +  CACTCCAGCCTGGGTGACAGAGCTAGACTCCATTTCAAAAAAAAAAAAAAAAAGGGAATTGTCCCAATGACCAATGGTTGGGATTTTTTACTT
                                                   *****************-****************************************
3´ end          AAAAAAAAAAAAAAAAAAAAAAAAAAAAAAAAAAACAAAAAAAAAAAAAAAA-GGGAATTGTCCCAATGACCAATGGTTGGGATTTTTTACTT
```

pHLE4. 8a

```
5´ end          GATACCTTGAGTTGAAACTCAGGCTGTATCAGGCACTAGTAATGTGACATAAATCATGCTGCTATAAAGACACATGCACACGTATGTTTATTGC
                **************************************************
hs_chrX +       GATACCTTGAGTTGAAACTCAGGCTGTATCAGGCACTAGTAATGTGACATGAACAATATTCTTACTATGTCTTGGCCTCAATTTCTACATCTTT

hs_chr2 +       GAAAGAAGAAAGAGAGAGAGAGAGAGAAAGAAAGAAAGAAAGAAAAAAAAAAGAAAAGAAAGAAAGAAAGAAAAAGAAAGAGAAAAAGAAATGA
                                                      ********************************************************
3´ end          AAAAAAAAAAAAAAAAAAAAAAAAAAAAAAAAAAAAAAAAAGAAAAAAAAAAGAAAAGAAAGAAAGAAAGAAAAAGAAAGAGAAAAAGAAATGA
```

pHLE4. 11a

```
5´ end          CTATGAGAAAAACAAGAGATAGGAAAAAGAGAGCTCTCCAAGCAATTAGTAAACTATCGCAAGAACAAAAAACCAAACACCGCATATTCTCACTCATAGGTGG
                ************************************************
hs_chr20 -      CTATGAGAAAAACAAGAGATAGGAAAAAGAGAGCTCTCCAAGCAATTAAAAACTATTAAAATTCCAGATTTAATTTAGCAATAAAATGGACATTGCTAAACAC
                                                               ********************************************************
3´ end          AAAAAAAAAAAAAAAAAAAAAAAAAAAAAAAAAAAAAAAAAAAAAAAAAAAACTATTAAAATTCCAGATTTAATTTAGCAATAAAATGGACATTGCTAAACAC
```

pHLE4. 12a

```
5´ end          GAGACCTTGTGCCAGGGGAACTCCTGTTTATAAAACTGTCAGATCGGCTTCAGACGATCAAATTACTCTGAGCTACGGGAG
                *********************************************
hs_chr21 -      GAGACCTTGTGCCAGGGGAACTCCTGTTTATAAAACTGTCAGATCTCATGAGACATATTCACTACCACGAGAACAGTATGG
                                             ****************************************************
3´ end          AAAAAAAAAAAAAAAAAAAAAAAAAAAAAATAAAACTGTCAGATCTCATGAGACATATTCACTACCACGAGAACAGTATGG
```

pHLE5. 8a

```
5´ end        TCTAATGTTGACAGTGGGGTGTTAAAGTCTCCCATTATTATGGTGTGCTATTTAATAAATGGTGCTGGGAAAACTGGCTAGCCATATGTAGAAAGCTG
              ***********************************************
hs_chr6 -     TCTAATGTTGACAGTGGGGTGTTAAAGTCTCCCATTATTATGGTGTGGGAGTCTAAGTCTCTTTGTAGGTCACTCAGGACTTGCTTAATGAATCTGGG
                                                     87633411
                                                                86521790
hs_chr6 -     AAGTTCCCACGAGTCCCCAAGAGAACAAACAGCCAAAATAATAATAATAATAATAATAATAATAATAATAATAATAATAATGAAGAAGCACAGGCTGA
                                                              **************************************************
3´ end        AAAAAAAAAAAAAAAAAAAAAAAAAAAAAAAAAAAAAAAAAAAAAAAAAATAATAATAATAATAATAATAATAATAATAATGAAGAAGCACAGGCTGA
```

pHLE5. 9a

```
5´ end        ACCCTTTTCCAATCTCCAATCTTCATACTGTCACCTCTGTTAACAAATGCACCATGATCAAGTGGGCTTCATCCCTGGGATGCAA
              *************************************************
hs_chr7 -     ACCCTTTTCCAATCTCCAATCTTCATACTGTCACCTCTGTTAACAAATGTGCATATGCTTCCTATTTATATAAAGTCCAAATTCC
                                                       ********************************************
3´ end        AAAAAAAAAAAAAAAAAAAAAAAAAAAAAAAAAAAAAAAAAAACAAATGTGCATATGCTTCCTATTTATATAAAGTCCAAATTCC
```

pHLE5. 10a

```
5´ end        GGTGAATTCTCAAGATGGCATATTTCAATATACTAAATCAGAAGAACTCGTCAAGAAGGCGATAGAAGGCGATGCGCTGCGAATCGGGAG
              **************************************
hs_chr1-      GGTGAATTCTCAAGATGGCATATTTCAATATACTAAATATAATAAATAATTGAAGAGGAAATATTTAATAATTAAATACAAAAGCTATAT
                                                    ***********************************************
3´ end        AAAAAAAAAAAAAAAAAAAAAAAAAAAAAAAAAAAAAAAAAAAAAATAATTGAAGAGGAAATATTTAATAATTAAATACAAAAGCTATAT
```

pHLE5. 14a

```
5´ end        GCCAGTGTCCAGGAACACAGAGACAACTGCTGTTTTAAACATTTTGACATGGATGAAATTGGAAACCATCATTCTCAGTAAACTATCGCAAGAACA
              ***********************************************
hs_chr13 -    GCCAGTGTCCAGGAACACAGAGACAACTGCTGTTTTAAACATTTTGAGACACAACCTTTCCAGATATCATTCACAAATACCACTATTCATAAAAAT
                                                  ************************************************************
3´ end        AAAAAAAAAAAAAAAAAAAAAAAAAAAAAAAAAAAAAAACATTTTGAGACACAACCTTTCCAGATATCATTCACAAATACCACTATTCATAAAAAT
```

pHLE5. 15a

```
5´ end      ATAACAGTCTCTCAGACAGCACAATCAAATTAGAATTCAAGATTAAGGTCGGGGGAGGGGGGAGGGATAGCATTGGGAGATATACCTAATGCTAGAT
            ***********************************************
hs_chr6 -   ATAACAGTCTCTCAGACAGCACAATCAAATTAGAATTCAAGATTAAGAAACACTCAACACTACACAGCTACATGGAAATTAACAACCTGCTCCTGAA
                                                        *****************************************************
3´ end      AAAAAAAAAAAAAAAAAAAAAAAAAAAAAAAAAAAAAAAAAAAAAAGAAACACTCAACACTACACAGCTACATGGAAATTAACAACCTGCTCCTGAA
```

pHLE5. 20b

```
5´ end      GCAAGATGGCAAACAACCTAAATGCCCTGAGCATACTGTTTACGCCATGGAATACTATGCAGCCATAAAAAATGATGAGTTCATA
            ******************************************
hs_chr5 +   GCAAGATGGCAAACAACCTAAATGCCCTGAGCATACTGTTTATTTAAAATGGATGCAGCAGATGGCTCATTCTTTGAAATTAGAA
                                                         ****************************************
3´ end      AAAAAAAAAAAAAAAAAAAAAAAAAAAAAAAAAAAAAAAAAAAAAAAAATGGATGCAGCAGATGGCTCATTCTTTGAAATTAGAA
```

pHLE5. 21a

```
5´ end      CCCCTTTCCTTGTTTTCTTATGGAATATTCTGTTTTTAAAACCTAATCATGATGGATGAAATTGGAAACCATCATTCTCAGTAAACTAT
            *****************************************************
hs_chr6 +   CCCCTTTCCTTGTTTTCTTATGGAATATTCTGTTTTTAAAACCTAATCATGATAATCAGTTTGTTGGATTATTAATATACTTCCTTTTT
                                                 ****************************************************
3´ end      AAAAAAAAAAAAAAAAAAAAAAAAAAAAAAAAAAAAAAAAACCTAATCATGATAATCAGTTTGTTGGATTATTAATATACTTCCTTTTT
```

pHLE6. 2a

```
5´ end      GTATAATGGTATGGCCACTATGGAAAACAATTAGGATGCGGAGAAATAGGAACACTTTTACACTGTTGGTGGGACT
            *********************************
hs_chr8 -   GTATAATGGTATGGCCACTATGGAAAACAATTATGGTATTTCCTTAAAAAAATAAAAAATAGAATCACCATGTGTA
                                                         *******************************
3´ end      AAAAAAAAAAAAAAAAAAAAAAAAAAAAAAAAAAAAAAAAAAAAAAAAAAAATAAAAAATAGAATCACCATGTGTA
```

pHLE6. 7a

```
5´ end        CAGAATGATATGACTTGAAATATAACTTATTATGAACATACTTGTAAAAGAGAAATGCAAATCAAAACCACTATGAGATATCATCTCACACCA
              ***************************************************
hs_chr18 +    CAGAATGATATGACTTGAAATATAACTTATTATGAACATACTTGTAAAAGAAAAAAAAATCCACCAGCAAAAAGTAATGTTAAAAATTATCAC
                                                           ************************************************
3´ end        AAAAAAAAAAAAAAAAAAAAAAAAAAAAAAAAAAAAAAAAAAAAAAAAAGAAAAAAAAATCCACCAGCAAAAAGTAATGTTAAAAATTATCAC
```

pHLE6. 11a

```
5´ end        TCCTGAATTACCACTGGGTCAAGGAAGCAATTAAGAAGGAAATTAAAAACCTTCTGGGGACTGTGGTGGGGTCGGGGGAGGGGGGAGGGATAG
              *******************************************************
hs_chr7 +     TCCTGAATTACCACTGGGTCAAGGAAGCAATTAAGAAGGAAATTAAAAACCTTCTTGGAACAAATAATAATGAAAACACAACATACCAAAACC
                                                          *************************************************
3´ end        AAAAAAAAAAAAAAAAAAAAAAAAAAAAAAAAAAAAAAAAAAAAAAAAACCTTCTTGGAACAAATAATAATGAAAACACAACATACCAAAACC
```

pHLE6. 18a

```
5´ end        TACCAAAAATATTTGAATTATTATGATCCCTTAATGCCAACACTTAAAATTAGATATACCTAATGCTAGATGACACATTAGTGGGTGCAGCGC
              ***************************************************
hs_chr5 -     TACCAAAAATATTTGAATTATTATGATCCCTTAATGCCAACACTTAAAATTCCTTTTTGATGAAAAGTCTGCAACATCTACAAAGTAAGTATC
                                                           ************************************************
3´ end        AAAAAAAAAAAAAAAAAAAAAAAAAAAAAAAAAAAAAAAAAAAAAAAAATTCCTTTTTGATGAAAAGTCTGCAACATCTACAAAGTAAGTATC
```

pHLE7. 3a

```
5´ end        CAAAGTTCAATGGTTTTCATATCCAGAATACAGTAAGAAATTGTGGTAGTAGAAAGCTGAAACTGGACCCCTTCCTT
              ************************************************
hs_chr9 -     CAAAGTTCAATGGTTTTCATATCCAGAATACAGTAAGAAATTGTGGTAAAAACTAAAATAAAAAAATAAAATAAATA
                                                *******************************************
3´ end        AAAAAAAAAAAAAAAAAAAAAAAAAAAAAAAAAAAAGAAATTGTGGTAAAAACTAAAATAAAAAAATAAAATAAATA
```

pHLE7.10a

```
5´ end              ACAATTCCCCGACTTTCTTGTGTTTTTTAAATTTAGTTTTTAAATTTAGCATATTCTCACTCATAGGTGGGAATTGAACAATGAGAT
                    ********************************************
hs_chrX -           ACAATTCCCCGACTTTCTTGTGTTTTTTAAATTTAGTTTTTAAAATCAAATGCATACTTGCAGTTTTAAAAATCAAATAGTTCTCCA
                                                            ***********************************************
3´ end              AAAAAAAAAAAAAAAAAAAAAAAAAAAAAAAAAAAAAAAATAAAATCAAATGCATACTTGCAGTTTTAAAAATCAAATAGTTCTCCA
```

pHLE7.28a

```
5´ end              TGCTCTGATGGAGCTGGGCAAAGTGAATTGAAAATCTTGAGATCTCTAGAAGCTGGGTACCAGCTGCTAGCAAGCTAGCTT
                    **************************************
hs_chr6 -           TGCTCTGATGGAGCTGGGCAAAGTGAATTGAAAATCTTCTGAAAAGGATTCCCCATTTTAGATGATGCCATTAATAATGTT
                                                 ****************************************************
3´ end              AAAAAAAAAAAAAAAAAAAAAAAAAAAAAGAAAATCTTCTGAAAAGGATTCCCCATTTTAGATGATGCCATTAATAATGTT
```

pHLE7.30a — U6 snRNA insertion at 5-end

```
5´ end              AGCATGGCCCCTGCGCAAGGATGACACGCAAATTCGTGAAGCGTTCCATATTTTTGCACACGTATGTTTATTGCGGCACTATTCACAATAGCAAAGACT

HLE7.30aL1.3Ct-R1   CATAATAAAATACTAAAACATCCATAATCGTCAATTGCTTCTGGTTTCTAAAAATTAGTGCTCGCTTCGGCAGCACATATACTAAAATTGGAACGATAC
                    *********************************************************
hs_chr6 -           CATAATAAAATACTAAAACATCCATAATCGTCAATTGCTTCTGGTTTCTAAAAATTATAGGCTATTTTCCTAATAGGTGCATTCATAACCCAGTCATCA
                                                                    ***************************************************
3´ end              AAAAAAAAAAAAAAAAAAAAAAAAAAAAAAAAAAAAAAAAAAAAAAAATAAAAATTATAGGCTATTTTCCTAATAGGTGCATTCATAACCCAGTCATCA
```

pHLE8.2c

```
5´ end              TAGAGGGCAGCATAAGAATGGGAATTGAGGTTAAAAGTAAGAAAATGTGGCACATATACACCATGGAATACTATGCAGCC
                    **************************************
hs_chr2 +           TAGAGGGCAGCATAAGAATGGGAATTGAGGTTAAAAGTGAGTATAAAAGTAAAGAATAGGATTTCATCAGGGTGAAAGTA
                                                                ************************************
3´ end              AAAAAAAAAAAAAAAAAAAAAAAAAAAAAAAAAAAAAAAAAAAAAAAAGTAAAGAATAGGATTTCATCAGGGTGAAAGTA
```

pHLE8. 3a

```
5´ end          AGATCAGAGCTGCTGCTGACTGCTAGTCAAAATGTGACATGATCAAAAAAAACCCTTCAAAAAATCAATGAATCCAGGAGCTGGTTTTTTGAA
                **********************************************
hs_chr13 -      AGATCAGAGCTGCTGCTGACTGCTAGTCAAAATGTGACATGATCAAGAAATAAACCTTTGTTGATGzTCAGATGTCAAAAGCCATTGAGTTTTT
                                                            *************************************************
3´ end          AAAAAAAAAAAAAAAAAAAAAAAAAAAAAAAAAAAAAAAAAAAAAAGAAATAAACCTTTGTTGATGTCAGATGTCAAAAGCCATTGAGTTTTT
```

pHLE8. 15a

```
5´ end          AAGGAATAATTGGATAATGGTATTGTGGTCATACTTTTTAAGTATGGCACATGTATACATATGTAACTAACCCGATCCGAACAAACGACCCAA
                ******************************************
hs_chr5 -       AAGGAATAATTGGATAATGGTATTGTGGTCATACTTTTTAAGAATATCTGTTAGAAAGATATAGGATGCAGAACATCTAGGATTTGCTGAAAG
                                                       ******************************************************
3´ end          AAAAAAAAAAAAAAAAAAAAAAAAAAAAAAAAAAAAAAAAAGAATATCTGTTAGAAAGATATAGGATGCAGAACATCTAGGATTTGCTGAAAG
```

pHLE8. 18a

```
5´ end          GAAAACCCCAACAAGGCCATTATAGTTAAATCACTGGAAACCAAAATTGGGAGATATACCTAATGCTAGATGACACATTAGTGGGTGCAGCG
                **********************************************
hs_chr4 +       GAAAACCCCAACAAGGCCATTATAGTTAAATCACTGGAAACCAAAAGAACATGTTAATGGCATCCAGGAGAAAAAATACATAGTATGAAGAG
                                                          **************************************************
3´ end          AAAAAAAAAAAAAAAAAAAAAAAAAAAAAAAAAAAAAAAAAAAAAAGAACATGTTAATGGCATCCAGGAGAAAAAATACATAGTATGAAGAG
```

pHLE8. 22a

```
5´ end          GGTACTGATTTTAAAAAGTGTGACTCTCCATCTACTTTGTCATGTGATAAACTAGAAAATCTAGAAGAAATGGATACATTCCTCGA
                *********************** **************************
hs_chr7 -       GGTACTGATTTTAAAAAGTGTGAATCTCCATCTACTTTGTCATGTGATAAGAATTTGTTTTTCAATCTCTTGATGAATCATTTGAT
                                                                **************************************
3´ end          AAAAAAAAAAAAAAAAAAAAAAAAAAAAAAAAAAAAAAAAAAAAAAAAAAGAATTTGTTTTTCAATCTCTTGATGAATCATTTGAT
```

pHLE8. 24b

```
5´ end        TTGTAGGCAAGTTGCAAAGTGCAATTAACAGATTAATGAACAGACGCACATATACACCATGGAATACTATGCAGCCATAAAAAATGATGAG
              **********************************************
hs_chr11 -    TTGTAGGCAAGTTGCAAAGTGCAATTAACAGATTAATGAACAGACGAAAGATGTGATACTTTTATTTAACTGGTCTTAATCACCCTCCATT
                                                           **********************************************
3´ end        AAAAAAAAAAAAAAAAAAAAAAAAAAAAAAAAAAAAAAAAAAAAAGAAAGATGTGATACTTTTATTTAACTGGTCTTAATCACCCTCCATT
```

pHLE8. 30a

```
hs_chr1 +     AAAGGATTATAATTCATGCTACTATAGAGACACATGCACACAAATGTTTATTGTGGCACTATTCACAATAGCAAAGACTTGGAACCAACC
                                                         ********** ************************************
5´ end        TAGGAGTTGCCACACTC-TTTTTTTTTTTTTTTTTGAAATGAAATGTTTATTGTGGCACTATTCACAATAGCAAAGACTTGGAACCAACC
              ***************** ***************************
hs_chr12 -    TAGGAGTTGCCACACTCTTTTTTTTTTTTTTTTTTGAAATGAAATCTCTCTCTTGTCCCCCAGTCTGGAGTGCAATGGGGCAATCTCTGC
                                                 *******************************************************
3´ end        AAAAAAAAAAAAAAAAAAAAAAAAAAAAAAAAAAAGAAATGAAATCTCTCTCTTGTCCCCCAGTCTGGAGTGCAATGGGGCAATCTCTGC
```

ZfL2-1 / DT40 WT cells

pWC1.1a

```
5´ end        AGAGACAGAGATAGGCAGCATACTGCCTTCTACAAAGGAACAAAGGTTGCATCAATCTTTAGTGATTTC
              ********************************************
gg_chr4 +     AGAGACAGAGATAGGCAGCATACTGCCTTCTACAAAGGAACAAAACAAAAAACATAAACACAAATCCCA
                                                   ********************************
3´ end        CTGAATTGAATTGAATTGAATTGAATTGAATTGAATTGAACAAAACAAAAAACATAAACACAAATCCCA
```

pWC1.2a

```
5´ end        ACTCAATTTTAAAAGGAAGTATAGTGAATGGAGAGACAGATTGACCTGGGGATACTCATCCCGAGGTCCTCAGATTAGGCGGAGTCACT
              ********************************************
gg_chr 3 -    ACTCAATTTTAAAAGGAAGTATAGTGAATGGAGAGACAGATTGAAAGCTTCAGAGAATGAAATACTCCTTTTATCTACTACAGGGGGAG
                                                     **************************************************
3´ end        GCTATACAAATAAAGCTGAATTGAATTGAATTGAATTGAATTGAAAGCTTCAGAGAATGAAATACTCCTTTTATCTACTACAGGGGGAG
```

pWC4.1a

```
5´ end        AGCCAGGCACCCTATGCCACCCAGACGACAAGGCTCCAGCTTACACGTATTGCAGACGAACAATATTTTTGAAGTGTTTCAG
              ************************************************
gg_chr19 -    AGCCAGGCACCCTATGCCACCCAGACGACAAGGCTCCAGCTTACACGTGTGCCCACCTAGAGGCGCTCCAAGACCGCAGCTG
                                                        ********************* ******************
3´ end        AAGCGCTATACAAATAAAGCTGAATTGAATTGAATTGAAAAAACACGTGTGCCCACCTAGAGGTGCTCCAAGACCGCAGCTG
```

pWC7.2a

```
5´ end        CAGAACAAAAATACTTCACAACCAGACGACCAAAAGGGATCATACAACAATAAATAACTGGAATCTTTTCTGGGAGCGTCCTAGGCTCTTCCGTC
              ******************************************************
gg_chr8 -     CAGAACAAAAATACTTCACAACCAGACGACCAAAAGGGATCATACAACAATAAAGAAAATGACAACAATGAATTAACACATTAGTAGATTAGAAG
                                                              ***********************************************
3´ end        TTGTAAAAGCGCTATACAAATAAAGCTGAATTGAATTGAATTGAATTGAATAAAGAAAATGACAACAATGAATTAACACATTAGTAGATTAGAAG
```

pWC9.1a

```
5´ end        GGGGGGGACCTTATTTGGATCCTTTTTTTATGTTTTACAGACATGGGATCAGCTTTCACTGCTATGCAGATGATACTCAA
              *****************************************
gg_chr1 -     GGGGGGGACCTTATTTGGATCCTTTTTTTATGTTTTACAGATTGTATTTCATTGTGCAAATGAAAATCTATTTCTTTTTT
                                                      ****************************************
3´ end         AAAAGCGCTATACAAATAAAGCTGAATTGAATTGAATTGAATTGTATTTCATTGTGCAAATGAAAATCTATTTCTTTTTT
```

pWC9.2a

```
5´ end        TATGAATATTTTCATACTTCACAGATTGTTTCTGTATCAAGTAATCAAGTAAAGGAGGTCGAGCCTT
              *******************************
gg_chr1 -     TATGAATATTTTCATACTTCACAGATTGTTTGAAAATATGCGTGACTTGTGGTAAGCTATGTCTCAG
                                      *******************************************
3´ end        AGCTGAATTGAAAAATTGAATTGAATTGTTTGAAAATATGCGTGACTTGTGGTAAGCTATGTCTCAG
```

pWC10.1a

```
5´ end        CCATTGCTTTCAACATGGTTTTTAACAACCTGTTGTATCGTTCAATTTTACCAGACTACTTCGCACCATCTGACTAGCAGGTAAAAATTCA
              *******************************************************
UCSC_gg_chr2 + CCATTGCTTTCAACATGGTTTTTAACAACCTGTTGTATCGTTCAATTTTACCAGAAGCTGGTGCATGATAGGGGATATGATAAATCCACTC

UCSC_gg_chr2 + CCATTGCTTTCAACATGGTTTTTAACAACCTGTTGTATCGTTCAATTTTACCAGAAGCTGGTGCGTGATAGGGGATATGATAAATCCACTC
                                                              **************** **************************
3´ end        GTAAAAGCGCTATACAAATAAAGCTGAATTGAATTGAATTGAATTGAATACCAGAAGCTGGTGCATGATAGGGGATATGATAAATCCACTC
```

pWC10.2a

```
5´ end        GTTGACACTGGTTGTCCCTGAATCAGAGAACATCAGCTGAGTTTGGATAGATGACGCTAATGATACATTAGGGCTCGCGTTTATGGATTTAATACACT
              ************************************************
gg_chr4 -     GTTGACACTGGTTGTCCCTGAATCAGAGAACATCAGCTGAGTTTGGATGTTGTGGAGAATTGAGGCATTCAAAATGTCTGTGGTTGTGGTCTGAGATC
                                                            ****************************************************
3´ end        ATCTACATTGTAAAAGCGCTATACAAATAAAGCTGAATTGAATTGAATGTTGTGGAGAATTGAGGCATTCAAAATGTCTGTGGTTGTGGTCTGAGATC
```

pWC10. 4a

```
5´ end          ATGTTACAAGGCAACAGCACACCCATACCACACATTATTCAAATCTTTTCTGGGAGCGTCCTAGGCTCTTCCGTCCTGACGGCCTGCACCCC
                *************************************
gg_chr4 –       ATGTTACAAGGCAACAGCACACCCATACCACACATTACACAGAATAAATCTATTCTTATCATCGGCTACAATTAAATCACGTTACAAAACTC
                                                    23245002
                                                        23242359
gg_chr4 –       GAAAACCAATCTGATCTTTATTTTTCACAGTTACTCTAAGGACACCACTTCATACGCAGCATATTTGAAACTGAATCTATTTACATTTATGA
                                                        ****************************************************
3´ end          AGCGCTATACAAATAAAGCTGAATTGAATTGAATTGAATTGACACCACTTCATACGCAGCATATTTGAAACTGAATCTATTTACATTTATGA
```

pWC10. 6a

```
5´ end          TGTGTCGTATCCTCCCAAGGGATGAAGCTTAGCTGTCATTTGCTGGGAGAAGCATCGATGGAGGCGTTGGAGCTGGAGCTGGAAGAAGTGGAGTCCCAGAT
                                        *****************
gg_chr1 –       TTACTTACCAGTCAATGACAAGAAAAGCTTAGCTGTCATTTGTTCAAGCTCATTTAACTTTTTTTCTTTTTTGATCTAGGATTTCACTCCGAAATCTGCAA
                                                                   **************************************************
3´ end          CATTGTAAAAGCGCTATACAAATAAAGCTGAATTGAATTGAATTGAATTGAATTTAACTTTTTTTCTTTTTTGATCTAGGATTTCACTCCGAAATCTGCAA
```

pWC11. 2a

```
5´ end          CAAAAGTAAGTGGGAAATGAGTTTCTGGCTTGTACTGTTTAATATTACAATAACTGGAATCTTTTCTGGGAGCGTCCTAGGCTCTTCCG
                **********************************************
gg_chr3 –       CAAAAGTAAGTGGGAAATGAGTTTCTGGCTTGTACTGTTTAATATTTTTCCTATTCTCATATTTGAAGATCTGTTTCAAACCCTCGCTG
                                                             ********************************************
3´ end          AAAGCGCTATACAAATAAAGCTGAATTGAATTGAATTGAATTGAATTTTCCTATTCTCATATTTGAAGATCTGTTTCAAACCCTCGCTG
```

pWC11. 6a

```
5´ end          AGCCACTGAGTGGCTGAGATGGTGCAGAGGGTGCAAGAAGGCGAGCGAATCTTTTCTGGGAGCGTCCTAGGCTCTTCCGTC
                ***********************************************
gg_chr17 +      AGCCACTGAGTGGCTGAGATGGTGCAGAGGGTGCAAGAAGGCGAGCGCTGTGCTGCTCCCCCTTCCCATGGGGCAGCCGTA
                                                          ***************************************
3´ end          AAAGCGCTATACAAATAAAGCTGAATTGAATTGAATTGAATTGAGCGCTGTGCTGCTCCCCCTTCCCATGGGGCAGCCGTA
```

pWC12.1a

```
5´ end      GAGCAGCTGAACCCATCCCAGACCGCCTGTGTTAAAGCTGCCAAGGTGCTTCCCAGATCCCGGGGCAGAACGTCTCCGCCTGTG
            **********************************************
gg_chr17-   GAGCAGCTGAACCCATCCCAGACCGCCTGTGTTAAAGCTGCCAAGGGAGAGCTAGCTTTATGTCCCTCCAGAGCCCTCCAACAC
                                                  **********************************************
3´ end      GCGCTATACAAATAAAGCTGAATTGAATTGAATTGAATTGCCAAGGGAGAGCTAGCTTTATGTCCCTCCAGAGCCCTCCAACAC
```

pWC12.4a

```
5´ end      TCCCACAGTGCAAAAAATTTCCAAAGTTTAAATTCTGATGAAATTGCTCTTTGCTAATAACTGGAATCTTTTCTGGGAGCGTCCTAGG
            ****************************************
gg_chr4 +   TCCCACAGTGCAAAAAATTTCCAAAGTTTAAATTCTGATGTTGTTTTTAAAGAGAAAGGCTAGTTTAAAATCAAGAATATAATTCATA
                                                 ***************************************************
3´ end      AGCGCTATACAAATAAAGCTGAATTGAATTGAATTGAATGTTGTTTTTAAAGAGAAAGGCTAGTTTAAAATCAAGAATATAATTCATA
```

pWC12.5a

```
5´ end      ATGTAGTTTTGGATTGTGGCCTGATGAAGCAAAGTAAATAAGAGTTATTGTATAAGAGTTATTCTCTATTTTCACCTGGGGATA
            **************************************************
gg_chr3 +   ATGTAGTTTTGGATTGTGGCCTGATGAAGCAAAGTAAATAAGAGTTATTGACAAGATGACGAACCTGAAAGCTTGGTTCTGCTT
                                                      ********** *********************
3´ end      AAGCGCTATACAAATAAAGCTGAATTGAATTGAAAAAAAAAAAGTTATTGACCAGATGACGAACCTGAAAGCTTCATATAGCAC
```

pWC13.1a

```
5´ end      TTTGCTTTGTGTTTGGAATTGATGTCTTCTCCACAGCTGCCTGTAGTGTACCATCTGACTAGCAGGTAAAAATTCACAAAATTCACACTATAG
            ***************************** ******************
gg_chr4 +   TTTGCTTTGTGTTTGGAATTGATGTCTTCCCCACAGCTGCCTGTAGTGGCACTGGGGATAGAGGGATGTTTGTACTTATTTCTTCTTTTAACT
                                                     ****************************************************
3´ end      AAAGCGCTATACAAATAAAGCTGAATTGAATTGAATTGAATTGTAGTGGCACTGGGGATAGAGGGATGTTTGTACTTATTTCTTCTTTTAACT
```

pWC13.2a

```
5´ end          AATCTACCTTGACTAGCAGCAGTGCCATCCTCTCTGCTTTTCTGGGAGCGTCCTAGGCTCTTCCGTCCTGACGGCCTGCA
                **************************************
gg_chr3 +       AATCTACCTTGACTAGCAGCAGTGCCATCCTCTCTGCTAGGAGTGAACTCAGTGCCATGCTGGGGCTTCAGGAAAGCAGA
                                                     *******************************************
3´ end          GCGCTATACAAATAAAGCTGAATTGAATTGAATTGAATAGGAGTGAACTCAGTGCCATGCTGGGGCTTCAGGAAAGCAGA
```

pWC13.4a

```
5´ end          AGGTTTTTTTCTTCCCACCCACTCCATTTTTTACTGCGTTGTTCACTTGTTATTAAGCCTATTATTAAGAAACCGCAACTAGACACCAACAA
                *************************************************
gg_chr14 +      AGGTTTTTTTCTTCCCACCCACTCCATTTTTTACTGCGTTGTTCACTTGGTCCTTTATAGGACAGGCTGCAAATTTACGAGGTTGCAGTGGA
                                                            ************************************************
3´ end          AAAGCGCTATACAAATAAAGCTGAATTGAATTGAATTGAATTGAACTTGGTCCTTTATAGGACAGGCTGCAAATTTACGAGGTTGCAGTGGA
```

pWC13.5a

```
5´ end          GGCACAGCACCCGAAGGGAATCACTACTTCTACCTGAGAAGGTCCTTTGTTCAGTAGACTTTTAGCTTTGAATGAATGGCTAATAACATGGTGTAAA
                ************************************* **************
gg_chr4 +       GGCACAGCACCCGAAGGGAATCACTACTTCTACCTGAAAAGGTCCTTTGTTCTCCATGAGCATCACATGCGGTCTGAGTAATGCTTCTTCTGCTTCT
                                                                 ********************* **************************
3´ end          TACATTGTAAAAGCGCTATACAAATAAAGCTGAATTGAATTGAATTGAATTCTCCATGAGCATCACATGCAGTCTGAGTAATGCTTCTTCTGCTTCT
```

pWC13.6a

```
5´ end          GAGAAGGCAAGCTTTGTTCTAGTTCTGCAACGCTCTGAAACTGTTTAGTAAAGCATCTGTCCAACACCCGTGCGTTTTATTCTGTCTTTTTA
                        *********************************
gg_chr2 -       ATGCTCTAAAGCTTTGTTCTAGTTCTGCAACGCTCTGAAACCATGAATTGTAACACATCGGCTACAAAACCCAACTGCTCCACCTGATACAG
                                                   *********************************************************
3´ end          CTATACAAATAAAGCTGAATTGAATTGAATTGAATTGAAACCATGAATTGTAACACATCGGCTACAAAACCCAACTGCTCCACCTGATACAG
```

pWC14.1a

```
5´ end      TGCAATCCACACATGCCTCACTGAGGCATGTTCACTTTCACTTCACAAAACAGAATTATTACTTATTGGGCCTAA
            ***************************
gg_chr7 -   TGCAATCCACACATGCCTCACTGAGGCTCCCAAGTGGAGTTTGCACTTGCTATTTAAATTAGTGTTTTTGAGCTT
                                 ******************************************************
3´ end      CTGAATTGAATTGAATTGAATTGAGGCTCCCAAGTGGAGTTTGCACTTGCTATTTAAATTAGTGTTTTTGAGCTT
```

pWC14.3a

```
5´ end      TGCATTTCCAACAAAAGCATTTCCCCCACAAGGAAATTTCTGGGAGCGTCCTAGGCTCTTCCGTCCTGACGGCC
            ********************************
gg_chr1 +   TGCATTTCCAACAAAAGCATTTCCCCCACAAGAACCAACTGAAGTGACACCTACGGAAGCACCAACTCAAATGT
                                       ***********************************************
3´ end      AAATAAAGCTGAATTGAATTGAATTGAACAAGAACCAACTGAAGTGACACCTACGGAAGCACCAACTCAAATGT
```

pWC14.5a

```
5´ end      ATTCTTGTCACATTATTCTTGCTCAGTGTCTCCTTCTTTAAAGCTCTATGCCACCTAGACTCTTGTTCACCCCACTTAAACATCAGTAACGC
            ***************************************************
gg_chr5 -   ATTCTTGTCACATTATTCTTGCTCAGTGTCTCCTTCTTTAAAGCTCTATGCAGGTACCTGTGGCATGCAGCACAACCTACCCGTAATGGAGC
                                                        ************************************************
3´ end      TGTAAAAGCGCTATACAAATAAAGCTGAATTGAATTGAATTGAATCTATGCAGGTACCTGTGGCATGCAGCACAACCTACCCGTAATGGAGC
```

pWC14.6a

```
5´ end                       TGTTGTGTTTTTGATTAGATCTAATTAGATCTAATTAGATCTTAATTAGATCTAATTAGATCTTAATTAGATCTACATTAGATCTAATTCTGTCTTATGGAAT
                             **** ********
gg_chr2 +   AGTTTATTGTTTGGTTTTGTTATGTTTTTGTTGTTTTTAAATTTATCTTCTTATCATGTTGAAAGCTTATAGTTTTTTGTAT
                                    **************************************** *****************
3´ end      AAAGCTGAATTGAATTGAATTGAATTTTTGTTGTTTTTAAATTTATCTTCTTATCATGTTGAAAACTTATAGTTTTTTGTAT
```

pWC15.1a

```
5´ end          TAATTAATTTATTAATATTAATTATTAATTTAGTAATTAGGAGTAATATTTGCTTTATAACCCTATAAAGCTCTAAATAATCTAGCTCCTGTTTATCT

5´ end          TTTTAAAGTTTCTAACTACAGTGACGAACAGGAGTAATATTTGCTTTAGTAATATGGAGTAATATTAATTATTAATTTATTAATATTAATTTATTAAT
                ************************ ************************
gg_chr1 +       TTTTAAAGTTTCTAACTACAGTGATGAACAGGAGTAATATTTGCTTTAGATAAACTGCACACTGA---------GTTTCTTGTTTTGTTTTTGTTGTT
                                                           **********************         ***** ******************
3´ end          GTAAAAGCGCTATACAAATAAAGCTGAATTGAATTGAATTGAACTTTAGATAAACTGCACACTGAGTTTTTTTGGTTTCGTGTTTTGTTTTTGTTGTT
```

(158 exrea nucleotides)
TAATATGGAGTAATATTAATTATTAATTTATTAATATTAATTTATTAATATTAATTTATTAATATTAATTAATTTATTTATTAATTTTAATTAAAATTAATTAATTTATTAATATTAATTATTAATTTAGTAATTAGGAGTAATATTTGCTTTATAAC

pWC15.2a

```
5´ end          GTTGCCTGTAATATCAGAAGATGTTAAAACAGGATATGTTTTAACATAATAACGAAATATGCT
                ***********************************
gg_chr2 +       GTTGCCTGTAATATCAGAAGATGTTAAAACAGGATGTTGGGGTGTTAGGAAAAGGTTCTTCAT
                                         **************************************
3´ end          AGCTGAATTGAATTGAATTGAATTGAAAACAGGATGTTGGGGTGTTAGGAAAAGGTTCTTCAT
```

pWC15.3a

```
5´ end          ACAAAAGGGTCATTTGAACAAGAGTTCTCTAGAAGCTGGGTACTAGCGGCCGCCACCATGGACTACAAAGACCATGACGGAGA
                **************************
gg_chr2 –       ACAAAAGGGTCATTTGAACAAGAGTTACTCCCTAAACTGGACCAAAAAGTCTTTTGATAACATTTATGTACATTTTGTAAGTG
                                     *****************************************************
3´ end          AAAGCTGAATTGAATTGAATTGAGTTACTCCCTAAACTGGACCAAAAAGTCTTTTGATAACATTTATGTACATT

'DELETION'
TK109-17        GACGGTGATTATAAAGATCATGACATCGACTACAAAGACGATGACGACAAGTCGCTTCCGcTCTCTGTCCTTGTGTGCAGGAGAAGCATCGATGGAGGCGTTGGAGCTGGA
                *****                                                                         ********************************
5´ end          ACAAAAGGGTCATTTGAACAAGAGTTCTCTAGAAGCTGGGTACTAGCGGCCGCCACCATGGACTACAAAGACCATGACGGAGAAGCATCGATGGAGGCGTTGGAGCTGGA
                **************************
gg_chr2 –       ACAAAAGGGTCATTTGAACAAGAGTTACTCCCTAAACTGGACCAAAAAGTCTTTTGATAACATTTATGTACATTTTGTAAGTG
                                     *****************************************************
3´ end          AAAGCTGAATTGAATTGAATTGAGTTACTCCCTAAACTGGACCAAAAAGTCTTTTGATAACATTTATGTACATT
                ***********************
TK109-17        AAAGCTGAATTGAATTGAATTGAATGGATCCAGACATGATAAGATACATTGATGAGTTTGGACAAACCACAACT
```

pWC16.1a

```
5´ end          TATGTATTTTAAGAGACACAGCGGGCTATAGTCTAATCAACTCTTATTCTCTTTATTCTCTATTTTCACCTGGGGATACTCATCCCGAG
                ******************************************
gg_chrZ +       TATGTATTTTAAGAGACACAGCGGGCTATAGTCTAATCAACTGCACAGAAACTATGATTAGTAAAAGTACTTCTGCGATGCAGTAAAGA
                                                      ***************************************************
3´ end          AAAGCGCTATACAAATAAAGCTGAATTGAATTGAATTGAACTGCACAGAAACTATGATTAGTAAAAGTACTTCTGCGATGCAGTAAAGA
```

pWC16.2a

```
5´ end          TTGTTGACTGATAAGATCAGTACAAACTCCAGTTACAGCATTCTTACACTCTGGAATAGCCTTCCTGGTAATGTCCGAGGCTCAGACACACTCTCCCAG
                **********************************************
gg_chr3 +       TTGTTGACTGATAAGATCAGTACAAACTCCAGTTACAGCATTCTTAATGAGAGAATATTTCTGTTTTGACACCAAGGCTTGTTTCTAAGTTATTTAAGG
                                                                             **************************************
3´ end          CTGCTTTGACACAATCTACATTGTAAAAGCGCTATACAAATAAAGCTGAATTGAATTGAATTGTTTTGACACCAAGGCTTGTTTCTAAGTTATTTAAGG
```

pWC16.3a

```
5´ end          GAATTGCTTCATATAGTAAGTATACTTACCACTAGTGAAAATCGCTTAAAGCATACATTAGATCTAATTCTGTCTTATGGAATCGAG
                *****************************************
gg_chr5 +       GAATTGCTTCATATAGTAAGTATACTTACCACTAGTGAAAACTGTTCCCCAAATAGGTAAAGAATGTTTTCAAGCATGTTATAGTGT
                                                   ****************************************************
3´ end          CTATACAAATAAAGCTGAATTGAATTGAATTGAATTGAAAACTGTTCCCCAAATAGGTAAAGAATGTTTTCAAGCATGTTATAGTGT
```

pWC21.1a

```
5´ end          CTCTACAGGAATCCACACGCATAACTTTCTTTAACCTGTGAGATCTCTAGAAGCTGGGAGAAGCATCGATGGA
                ****************************
gg_chr10 -      CTCTACAGGAATCCACACGCATAACTTTAACCTTCCAGATAGCATCTGCAGTCCTGACCATTGTAACCACACA
                                          ************* *********************************
3´ end          TACAAATAAAGCTGAATTGAATTGAATTAACCTTCCAGACAGCATCTGCAGTCCTGACCATTGTAACCACACA

DELETION'

TK109-17        ACATCGACTACAAAGACGATGACGACAAGTCGCTTCCGTCTCTGTCCTTGTGTGCAGGAGAAGCATCGATGGA
                                                                        *****************
5´ end          CTCTACAGGAATCCACACGCATAACTTTCTTTAACCTGTGAGATCTCTAGAAGCTGGGAGAAGCATCGATGGA
                ****************************
gg_chr10 -      CTCTACAGGAATCCACACGCATAACTTTAACCTTCCAGATAGCATCTGCAGTCCTGACCATTGTAACCACACA
```

pWC21.3a

```
5´ end          AGTTGGGTTGCAGAAAGGGTTTATGGATGGCTTACTGTCTTCCCACTGCCTTGTGTGCAGGAGAAGCATCGATGGAGGCGT
                *************************************************
gg_chr15 -      AGTTGGGTTGCAGAAAGGGTTTATGGATGGCTTACTGTCTTCCCACTGCTCAGATTCAGCAGCAGCAATACGTCTTAAATT
                                                              ***********************************
3´ end          GTAAAAGCGCTATACAAATAAAGCTGAATTGAAAATTGAATTGAATTGCTCAGATTCAGCAGCAGCAATACGTCTTAAATT
```

pWC22.1a

```
5´ end          AGCTTGTCCATGTGAACTATAAGGCATCACCCAGATTAGAGGGATTCAGCTGTGATCTCTAGAAGCTGGGTACTA
                *******************************************************
gg_chr 24 -     AGCTTGTCCATGTGAACTATAAGGCATCACCCAGATTAGAGGGATTCAGCTGTGAGTTGCTAACCCAATGCATCT
                                                                    ***********************
3´ end          TCTACATTGTAAAAGCGCTATACAAATAAAGCTGAATTGAATTGAATTGAATTGAGTTGCTAACCCAATGCATCT
```

ZfL2-1 / DT40Ku70-/- cells

p7C2. 1a

```
5´ end        AAATTATTTGCTTTCCTTTATTTTACTGAATGCAGCTTTGGGCAGTACAAAGGGTCAGCTTTCACTGCTATGCAGATGATACTCAATTATATATTTCCACTA
              ****************************************************
gg_chr14 -    AAATTATTTGCTTTCCTTTATTTTACTGAATGCAGCTTTGGGCAGTACAAAGAAAATCCTCACAAGGGCACACAAGCACTTTAATGAGGCCAAGAAGTGTTG
                                                        1098029
                                                                      1093646
gg_chr14 -    AGTCATTTCCAACTGAAAGTTTGTGCGCATCAAACACAGGTAACACCCCAGGAACAGCTAGTGATGCTTCCTGACCACTTCCATCCTGTGCTCCCGGCCCTT
                                                                        ********************************************
3´ end        AATCTACATTGTAAAAGCGCTATACAAATAAAGCTGAATTGAAAAAAATTGAATTGAATAGTGATGCTTCCTGACCACTTCCATCCTGTGCTCCCGGCCCTT
```

p7C3. 1a

```
5´ end        GTAACATCTTTTTTACACCTGCTCAAACTTGAACCATTGGATTGGTTTTCTGAGGATCCCCTCAGAAGAACTCGTCAAGAAGGCGATAGAAGGCGATG
              ***************************** ********
gg_chrZ -     GTAACATCTTTTTTACACCTGCTCAAACTCGAACCATTCTGGGAAGTTCTACTCTTACATTTATCTAGCTTTTACTACAGCATAGTCAGGTCTAGATT
                                                 13282880
                                                                    13282674
gg_chrZ -     AGGCTGTAAACCTCTGAAATGTACAAGCCAGATAGCTAAGTGAGGGTGGGAAGTGCAAGGGAAAGCAGAAGACTGCCTTTAGTCTGCAGAGAAAAACA
                                                                ************************************************
3´ end        CTACATTGTAAAAGCGCTATACAAATAAAGCTGAATTGAATTGAATTGAAAAGTGCAAGGGAAAGCAGAAGACTGCCTTTAGTCTGCAGAGAAAAACA
```

p7C7. 2a

```
5´ end        CTACAGTCACAGAGCACTGGGCATCATATGGCTGCAACTCGCTCCTTAAGATCTCAAAATTCAGGGCTTCTGGTAGTACCTAGAATA
              ***********************************
gg_chr3 -     CTACAGTCACAGAGCACTGGGCATCATATGGCTGCTGGGGATTCCTGCAATCTGTTACGGTTGTGGTAGCCAAGTTCTCCTGAAGTG
                                       **************************************************************
3´ end        ATAAAGCTGAATTGAATTGAATTGAATATGGCTGCTGGGGATTCCTGCAATCTGTTACGGTTGTGGTAGCCAAGTTCTCCTGAAGTG
```

p7C24. 1a

```
5´ end        AAGCTTAAGCTATAGGAGATAATGAGAAGTTCATTCTCTATTTTCACCTGGGGATACTCATCCCGA
              ******************************
gg_chr1 -     AAGCTTAAGCTATAGGAGATAATGAGAAGTGCGTTAGAAATCACAAAAAGTGAAAGACGTGTTCAG
                                  **********************************************
3´ end        AGCTGAATTGAATTGAATTGAATGAGAAGTGCGTTAGAAATCACAAAAAGTGAAAGACGTGTTCAG
```

p7C34. 2a

```
5´ end        AAAACTTCGCCTCTCTGAAGTCATGTACTTAGAGAATATTAATATTAATATTAAACAGAGAAACGGACTTATGTTTAGCTCCTTTGAAG
              **************************************
gg_chr1 -     AAAACTTCGCCTCTCTGAAGTCATGTACTTAGAGAATAATCTCAACACTTATTTAAAATTAAATGTTGCTTCGGAAATACTTGAATATG
                                              160093519
                                                        160092367
gg_chr1 -     GGGATGGTGGCAACTCACAGCCAGAGGAACTATCCTGTCTGCGTACAGCCGACTGGTAACAGATAAACATAATATTTTGGATAAATGGA
                                                            *******************************************
3´ end        GCGCTATACAAATAAAGCTGAATTGAATTGAATTGAATTGAATTGAAGCCGACTGGTAACAGATAAACATAATATTTTGGATAAATGGA
```

p7C38. 1a

```
5´ end        AGGGAGGGTTATTCCTTGGGGAAAAAAATAAGTTATCAGCAATTTAATAATAATCATAACAATCCTAGATTTTTATTTAACACCATCTCTAAATTAGCA
              *****************************************************
gg_chr2 -     AAAGGGAGGGTTATTCCTTGGGGAAAAAAATAAGTTATCAGCAATTTAATAATGCTTTAATTTGTCTCTGTTATACTGACTTGTATCTTCTATTCTCTT
                                                        145680423
                                                                   145679331
gg_chr2 -     AAAACAGCATAGAAGGAGAAAAATGTGCAGTGGTTGGGAGAACCAATCCTACTTACACACAGGACAAAGCAGAGGGCACCAGAAACATTTGGCCTTGAA
                                                                  ******************* ***************************
3´ end        ATCTACATTGTAAAAGCGCTATACAAATAAAGCTGAATTGAATTGAATTGAATTACACACAGGACAAAGCAAAGGGCACCAGAAACATTTGGCCTTGAA
```

p7C39. 3a

```
5´ end        AAAAAATCTTGTATTAATTCAGATCTCTAGAAGCTGGGTACTAGCGGCCGCCACCATGGACTACAAAGACCATGACGGAGAAGCATC
              *********************
gg_chr5 -     AAAAAATCTTGTATTAATTCACAGCACTCAGTAAACTTAAATGATATTTTTTCTTCTGAAATGAATTCCATTACAGAGAGTCCTATG
                         *******************************************************
3´ end        CTGAATTGAATTATTAATTCACAGCACTCAGTAAACTTAAATGATATTTTTTCTTCTGAAATGAAT
```

p7C42. 1a

```
5´ end        ATTGATCTTACTGTCCATAATTAAATCTAAAATTATGAACTATTATTAAAAATTAGAACTATTG
              ****************************
gg_chr4 -     ATTGATCTTACTGTCCATAATTAAATCTGAATGATTCCAAGATGCTACTAAAGTGACCTGGATT
                                **********************************************
3´ end        CTGAATTGAATTGAATTGAATTAAATCTGAATGATTCCAAGATGCTACTAAAGTGACCTGGATT

TK109-17      TATAAGCTATGTTTACCTGAAATCAGCAAACCCGCTCCAATACTCCGCCCTAGTAGAACTATTG
                                                                   ***********
5´ end        ATTGATCTTACTGTCCATAATTAAATCTAAAATTATGAACTATTATTAAAAATTAGAACTATTG
              ****************************
gg_chr4 -     ATTGATCTTACTGTCCATAATTAAATCTGAATGATTCCAAGATGCTACTAAAGTGACCTGGATT
                                **********************************************
3´ end        CTGAATTGAATTGAATTGAATTAAATCTGAATGATTCCAAGATGCTACTAAAGTGACCTGGATT
              *********************
TK109-17      CTGAATTGAATTGAATTGAATGGATCCAGACATGATAAGATACATTGATGAGTTTGGACAAACC
```

p7C43. 1a

```
5´ end        GAAATTTAGTGTGCAGGTAATCCTGTACAGAACAAATACTGTGTAACATCATTCCCTCTACCTCAACCCCGCGTCCTTCTCTGTCCAGG
              ************************************************
gg_chr 15 –   GAAATTTAGTGTGCAGGTAATCCTGTACAGAACAAATACTGTGTAACAGAGCCTGCTCCGAGGGTCTGAAGCTCCTCTACCCACCTCAA
                                                  8446315
                                              8446157
gg_chr 15 –   GTGCATGAAAATACCAGGTTTAGACTCCAACAGAAGGCACATGTACTGAAATAAAATGGAAATAAAAAGAGTTCTGAAAGGTTTCAGGT
                                                      *************************************************
3´ end        AAAAGCGCTATACAAATAAAGCTGAATTGAATTGAATTGAATGTACTGAAATAAAATGGAAATAAAAAGAGTTCTGAAAGGTTTCAGGT
```

p7C44. 1a

```
5´ end        GAAATTNTGATGTAATTCAAAAATTACAATTCAAAAAAAAAAAAAAACCCGCGCAAAC
              ******.************
gg_chr6 +     GAAATTCTGATGTAATTCATGTACAACATCTAAATAATAGTGACTGACAGTAAGGTT
                          *********************************************
3´ end        AATTGAATTGAATAATTCATGTACAACATCTAAATAATAGTGACTGACAGTAAGGTT
```

p7C45. 1a

```
5´ end        ATGGACAAAATCCTTGCTTGTAGCATCTTCAGTGTCTGACAGAATAGCAAGGTCGGTGAAAATCAGCAAACCCGCTCC
              ***************************** ********************** ******
gg_chr3 +     ATGGACAAAATCCTTGCTTGTAGCATCTTTAGTGTCTGACAGAATAGCAAGGCCGGTGAGTCACCCTCAAGCCATGCG
                                                               66184076
                                                               66185500
gg_chr3 +     GTGGATGTGGGTAACTCTACACTGTGCAGCAGTCCACGGTATGGACAAGCAGCAGGATTTAAGTGCTTGGAGTTAACT
                                                                     ***********************
3´ end        TATACAAATAAAGCTGAATTGAATTGAATTGAATTGAATTGAATTGAATTGATTTAAGTGCTTGGAGTTAACT
```

p7C47. 1a

```
5´ end        CTACATTTTGGAAGCATATGTATATTTTTCACTGCAAGTATGCATTTAATAATAAGTTCTGACCAGGTCTAGAAAATATGATCATATAACCCC
              **********************************************
gg_chr1 –     CTACATTTTGGAAGCATATGTATATTTTTCACTGCAAGTATGCATTGCTAGAATAATATGCAGATACAGGAGGTTATAATGGGATCTATTCAA
                                                         **************************************************
3´ end        TGTAAAAGCGCTATACAAATAAAGCTGAATTGAATTGAATTGAATTGCTAGAATAATATGCAGATACAGGAGGTTATAATGGGATCTATTCAA
```

p7C48.1a

```
5´ end      ATTTGAACAGTTTCTTCTCCTTTTTAACAAACCTAGAATAAAGACAGTCTACAGGTGTCCAGGGACAGGTCTACAATGGTTTAAGTCATACT
            ***********************************************
gg_chr3 -   ATTTGAACAGTTTCTTCTCCTTTTTAACAAACCTAGAATAAAGACAGAGGTGCTGCCTACCCGTTTTGTAACATACGGAGGCTCTCAAGTGG
                                                           *********************************************
3´ end      CATTGTAAAAGCGCTATACAAATAAAGCTGAATTGAATTGAATTGAAAGGTGCTGCCTACCCGTTTTGTAACATACGGAGGCTCTCAAGTGG
```

p7C49.1a

```
5´ end      CCAGGTGCTCAATCAGGATTCAATCAGGAATCAATCAGGATCAAATCATGGAAAAAAACTAATTTAGA
            ****************
gg_chr8 -   CCAGGTGCTCAATCAGTGGTTCAGGCCGTGACTCAGCAGTTACCATACAGATGCTAACACCTCTGGCT
                            ******************* ********************************
3´ end      TTGAATTGAATTGAATTGGTTCAGGCCGTGACTCAACAGTTACCATACAGATGCTAACACCTCTGGCT
```

p50.1a

```
5´ end      ACTCTTTTAGCTGGTGGGACAACAAGTTTTACTACCATGATCTCTAGAAGCTGGGTACTAGCGGCCGCCACCA
            ****************************************
gg_chr4 +   ACTCTTTTAGCTGGTGGGACAACAAGTTTTACTACCATGAAATGGCATTAAAAGCCACAGACTTCCCAGCTAA
                                                   **********************************
3´ end      AAAGCGCTATACAAATAAAGCTGAATTGAATTGAATTGAAAATGGCATTAAAAGCCACAGACTTCCCAGCTAA
```

p7C51.1a

```
5´ end      CTAGGTTGTTCATAGTACTAATCAAACAGACTCATGCTAACCATAGAGATTACCTCAGAAGAACTCGTCAAGAAGGCGATAGAAGGCGATGC
            *************************************************
gg_chr3 -   CTAGGTTGTTCATAGTACTAATCAAACAGACTCATGCTAACCATAGAGAAAATTAAAACATATCATGGACTTCTGATTAACCAGATCTCTTA
                                                           *********************************************
3´ end      TTGTAAAAGCGCTATACAAATAAAGCTGAATTGAATTGAATTGAATTGAAAATTAAAACATATCATGGACTTCTGATTAACCAGATCTCTTA
```

p7C52. 1a

```
5´ end       CCAAAAAACAATCCAGCTAGTTTCTAACCATAATGAGAAATTCACCAAGGACCAAAACTGGAATCTTTTCTGGGAGCGTCCTAGGCTC
             **********************************************************
gg_chr2 +    CCAAAAAACAATCCAGCTAGTTTCTAACCATAATGAGAAATTCACCAAGGACCAAAACATGTTTGTGTAGTCTATACATACTCAGAAT
                                                                     47867647
                                                       47868106
gg_chr2 +    AGAGCACCGTCTTTTGAAAGTGTATTTTAACTAAATAGTTTAATAACATCATGTGCAATTTCTTAGTTCCCACAAGGTGGGCAACATT
                                                     ************************************************
3´ end       AAAGCGCTATACAAATAAAGCTGAATTGAATTGAATTGAATAATAACATCATGTGCAATTTCTTAGTTCCCACAAGGTGGGCAACATT
```

p7C55. 1a

```
5´ end       TACAGCATAAAAGACTAAAAAAAAAAGACTAAAAAAAAGACATAAAAGACACTTAAACACTTAAACAT
             *****************
gg_chr2 +    TACAGCATAAAAGACTACTAAGTGTAATCTAAAAGTCTGTGTCACATCCCAGCAAAAGTGATTTCAC
                            ****************************************************
3´ end       TTGAATTGAATTGAATACTAAGTGTAATCTAAAAGTCTGTGTCACATCCCAGCAAAAGTGATTTCAC
```

p7C61. 1a

```
5´ end       TACATTTACCTCTGCAGTTTTATTGTAGCCAGATATAATGCAGATATAATGTAGCCACCTAGACTCTTGTTCACCCCACTTAAACATCAGTAAC
             *** ******************* **************** ****
gg_chr2 +    TACGTTTACCTCTGCAGTTTTATCGTAGCCAGATATAATGTAGATCAGCTTAATACCACCAAAGACAGCATTTAAAAAGCAGCAGTCTTTTAAG
                                                ******** ***************************** ************
3´ end       AAGCGCTATACAAATAAAGCTGAATTGAATTGAATTGAATTGAATCAGCTTTATACCACCAAAGACAGCATTTAAAAAGCAACAGTCTTTTAAG
```

p7C62. 1a

```
5´ end       CGCTGTGGCCCGCAGACTTGCCCGAGTGAAGCTGTAGCTTTGTGATACCCTTGAGATCTCTAGAAGCTGGGTACTAGCGGCCGCCA
             ***************************************************
gg_chr13 -   CGCTGTGGCCCGCAGACTTGCCCGAGTGAAGCTGTAGCTTTGTGATACCCTCCAAATGTTGCTGAATTTATCTTTTCAGCACACCT
                                                         ******************************************
3´ end       AAGCGCTATACAAATAAAGCTGAATTGAATTGAATTGAAATTGAATACCCTCCAAATGTTGCTGAATTTATCTTTTCAGCACACCT
```

p7C63. 1a

```
5´ end        CTTGTAGCACTCTGTGTACTCATACTCTTCATTTACATCACAGTAGATCTCGCAACTCAATTTACAATTAGAGGGATACAAAGTTAGCTTTAG
              ********************************************
gg_chr12 -    CTTGTAGCACTCTGTGTACTCATACTCTTCATTTACATCACAGTCAAAAGGCAGTGACACAAGAAAATCGGTGTATATGCTCCTGTTTATCTG
                                                       4045457
                                                       4041036
gg_chr12 -    ATTCATTTGCTCAAAAACTAAACCTAGAGTGTATTTTCAGAAACACATCTGAATCTCAACGTCAAATGTTGCAAATAGAGATTAACTACCTAT
                                                          *************************************************
3´ end        AAAGCGCTATACAAATAAAGCTGAATTGAATTGAATTGAATTGAACATCTGAATCTCAACGTCAAATGTTGCAAATAGAGATTAACTACCTAT
```

ZfL2-1 / HeLa-RC cells

Ra102

```
5´ end           TGTGTTCAGGAAGAACTGACTCCTTTATCATTATTTTAGAAGCTGGGTACTAGCGGCCGCCACCATGGACTACAAAGACCATGAC
                 *************************************
hs_chr13 +       TGTGTTCAGGAAGAACTGACTCCTTTATCATTATTTTGACCTTCTTTATGTCTGATAACATTCCTTGCTTTGAAATTACTCTGTC
                                                              ****************************************
3´ end           AAAGCGCTATACAAATAAAGCTGAATTGAATTGAATTGAATTGAATTATGTCTGATAACATTCCTTGCTTTGAAATTACTCTGTC
```

Ra103

```
5´ end           TTGCATGTCTCTAATGATCAGTGAAGTTGAGCTTTTTTTCATACAGGTGTCCAGGGACAGGCTCTACAATGGTTTAAGTCA
                 *********************************************
hs_chr11 -       TTGCATGTCTCTAATGATCAGTGAAGTTGAGCTTTTTTTCATACATTTGTTGGCCACATAAATATCTTCTTTAGAGAAGTG
                                                              ************************************
3´ end           ATAAAGCTGAATTGAATTGAATTGAATTGAATTGAATTGAATACGTTTGTTGGCCACATAAATATCTTCTTTAGAGAAGTG
```

Ra105

```
5´ end           AGCCCAGCTAACACATGCCATAAAAGGACAGAGTAGAGTGGAGAAGAGTAGAATAATCTAGCTCCTGTTTATCTAACCAACCTTCTGTCTCGC
                 *****************************************************
hs_chr9 -        AGCCCAGCTAACACATGCCATAAAAGGACAGAGTAGAGTGGAGAAGAGTAGAAAAGATATCTTTGGTACAATGAAGAATATTTACCTGAGTTC
                                                                   *******************************************
3´ end           AATCTACATTGTAAAAGCGCTATACAAATAAAGCTGAATTGAATTGAATTGAAAAGATATCTTTGGTACAATGAAGAATATTTACCTGAGTTC
```

Ra112

```
5´ end           CAAGAGATTGAATGGAGTCAACTTGGAGAGTAGAGGCAGCCTGTAACTCGCTCCTTAAGATCTCAAAATTCAGGGCTTCTGGTAGTACC
                 ********************************************
hs_chr13 -       CAAGAGATTGAATGGAGTCAACTTGGAGAGTAGAGGCAGCCTGTTTAGAGGAAAATGGGAAAAATGGGAATGGAACCTGGATAAATGCA
                                                            **********************************************
3´ end           ACATTGTAAAAGCGCTATACAAATAAAGCTGAATTGAATTGAATTTAGAGGAAAATGGGAAAAATGGGAATGGAACCTGGATAAATGCA
```

Ra115

```
5´ end        TTCTTTCTAATCCTTCTAATAACCTAGAGAAGTGAGGAAGTAGCAAAATCAAGTAAAGGAGGTCGAGCCTTCTCTTTCATGGCTCCTACA
              ******************************************
hs_chr1 +     TTCTTTCTAATCCTTCTAATAACCTAGAGAAGTGAGGAAGTATCCTTATCCGCAGCCTACAGATGAGAAAACTGAGGCTCAGCGAAGAGC
                                                           *********************************************
3´ end        CTACATTGTAAAAGCGCTATACAAATAAAGCTGAATTGAATTGAATTATCCGCAGCCTACAGATGAGAAAACTGAGGCTCAGCGAAGAGC
```

Ra121

```
5´ end        AAAAAAAAAAAAACACACACAAAGAAAATAAATCTTACCATTATTTAAGGAGATTATTAAGAAACCGCAACTAGACACCAACAACTTAGCTAACT
              *******************************************************
hs_chr10 -    AAAAAAAAAAAAACACACACAAAGAAAATAAATCTTACCATTATTTAAGGAGATTTTTAAAAAAATAGTTACTGTGTAAGGGAAGGTCTTCAAAA
                                                               **********************************************
3´ end        ATCTACATTGTAAAAGCGCTATACAAATAAAGCTGAATTGAATTGAATTGAGATTTTTAAAAAAATAGTTACTGTGTAAGGGAAGGTCTTCAAAA
```

Ra123

```
5´ end        CTTTTGGGCAAAGATATGGTGGAAATAATGCTGTAGATACACTCTCCCAGTTCAAAACTAGATTAAAGACCTATCTGTTTAGTAAAGCA
              *************************************
hs_chr6 +     CTTTTGGGCAAAGATATGGTGGAAATAATGCTGTAGAGCCTTATAATGATGATGACTTTTTATCACTTGGCTAAGATAGTGCCTGCCAC
                                                      *************************************************
3´ end        AAAGCGCTATACAAATAAAGCTGAATTGAATTGAATTGAATTATAATGATGATGACTTTTTATCACTTGGCTAAGATAGTGCCTGCCAC
```

Ra124

```
5´ end        GGACAGGGGAGGAAGAGAGCCCCAGCCCCCTTTTCCACTCTGCATCTTTATTCTCTATTTTCACCTGGGGATACTCATCCCGAGGTCC
              **********************************************
hs_chr7 +     GGACAGGGGAGGAAGAGAGCCCCAGCCCCCTTTTCCACTCTGCATCTCCTCAAGGTCCAGGTGACTATGGGACCCGAGGAGGTGCTGA
                                                         *********************************************
3´ end        TGTAAAAGCGCTATACAAATAAAGCTGAATTGAATTGAATTGAATCTCCTCAAGGTCCAGGTGACTATGGGACCCGAGGAGGTGCTGA
```

Ra125

```
5´ end                CTCAGGCCGCCCTCAGGGCTGATTTAGACTACTTCGCACCATCTGACTAGCAGGTAAAAATTC
                                *************************
hs_chr1 +     GGTGATGATCTGGGGAAACTCAGGCCGCCCTCAGGGCTGATTTTGATCTTTGCTATTGGACTCTGTGACACTGTCAGTCTG
                                                     ******************************************
3´ end         GCTATACAAATAAAGCTGAATTGAATTGAATTGAATTGAATTTTGATCTTTGCTATTGGACTCTGTGACACTGTCAGTCTG
```

Ra126

```
5´ end        GGTGTGTAGTAGGCTATAATATCTAGGTTTATGTATGTACACGCTAAGATGTTTGCACTCTTTATTCTCTATTTTCACCTGGGGATACTCATCCCGAGG
              **********************************************************
hs_chr12 -    GGTGTGTAGTAGGCTATAATATCTAGGTTTATGTATGTACACGCTAAGATGTTTGCACAATGACAAAATTATCTAACAATGCATTTCTCATAATGCATT
                                                                      *******************************************
3´ end        ACACAATCTACATTGTAAAAGCGCTATACAAATAAAGCTGAATTGAATTGAATTGAACAATGACAAAATTATCTAACAATGCATTTCTCATAATGCATT
```

Ra201

```
5´ end        TTTGAAAAAAAGGTGGGACATAATGAACTCATGAGTAGATACCTATCATAGGCTCTTCCGTCCTGACGGCCTGCACCCCAGTCGAGCCGGAGCTGAAC
              **************************************************
hs_chr8 +     TTTGAAAAAAAGGTGGGACATAATGAACTCATGAGTAGATACCTATCATAATTACACATGCATCCTGTCTTTCTGTGCTAGTATTGTTGAAGATAGCT
                                                                     *******************************************
3´ end        ACAATCTACATTGTAAAAGCGCTATACAAATAAAGCTGAATTGAATTGAATTGAACATGCATCCTGTCTTTCTGTGCTAGTATTGTTGAAGATAGCT
```

Ra203

```
5´ end        TTGAGTTCCTTGGTACATGCCTAAGATGTGCAGATTTGTTACATAGGTAAATAGGAGATGCCAAACTTTCTGCACCGGCTTATACTCCAAATCC
              *****************************************************
hs_chr1 -     TTGAGTTCCTTGGTACATGCCTAAGATGTGCAGATTTGTTACATAGGTAAATATTTGCCATGGTGGTTTGCTGCACCTATCAACCTATCACCTA
                                                                  ******************************************
3´ end        ACATTGTAAAAGCGCTATACAAATAAAGCTGAATTGAATTGAATTGAATTGAATTTGCCATGGTGGTTTGCTGCACCTATCAACCTATCACCTA
```

Ra205

```
5´ end         AGACGGGCGGAGAGAAACCCGGGAGGCTAGGGACGGCCTGAAGGCCTTCTTTCATCTGAGAAATATCGCTAAATTACGAAATATGCTA
               *********************************************
hs_chr12 +     AGACGGGCGGAGAGAAACCCGGGAGGCTAGGGACGGCCTGAAGGCGGCAGGGGCGGGCGCAGGCCGGATGTGTTCGCGCCGCTGCGGG
                                                       ************************************************
3´ end          AAGCGCTATACAAATAAAGCTGAATTGAATTGAATTGAAAAAGGCGGCAGGGGCGGGCGCAGGCCGGATGTGTTCGCGCCGCTGCGGG
```

Ra207

```
5´ end         TTAAACTCAGTTTGTGGATATCCTCACGATAACTTGCTGAGACCTAGACTCTTGTTCACCCCACTTAAACATCAGTAACGCAT
               ******************************************
hs_chr2 +      TTAAACTCAGTTTGTGGATATCCTCACGATAACTTGCTGAGATTTTGATTGAGATTGCATTGAATCTGTAGATGAAGTTGGAA
                                                       *******************************************
3´ end          GTAAAAGCGCTATACAAATAAAGCTGAATTGAATTGAATTGATTTTGATTGAGATTGCATTGAATCTGTAGATGAAGTTGGAA
```

Ra227

```
5´ end         CATGTACAACAATACCTTCACAGAGCTAAGAAACCAGGTAAGAGATTACACTGATTGGATCTACAAACGACCCAACACCCGTGCGTTTTA
               **************************************************
hs_chr18 −     CATGTACAACAATACCTTCACAGAGCTAAGAAACCAGGTAAGAGATTACAGCACCTGGGTATAGCACAGAAATAAGAAAAGGCACACTGA
                                                             ********************************************
3´ end          AAAAGCGCTATACAAATAAAGCTGAATTGAATTGAATTGAATTGAATACAGCACCTGGGTATAGCACAGAAATAAGAAAAGGCACACTGA
```

Ra230

```
5´ end         GGTATAGAGAGAGTTCAAAACTAGATTAAAGACCTATCTGTTTAGTAAAGCATACACTCAG
               **************
hs_chr7 +      GGTATAGAGAGAGTGATAATGGAGGTAGGTTTTACATAGGGTTAAAAATAAAATGTTTAATTATGACAAGGCTGTTAAGAAGGATCAAAAGCAGAGAGGAGAGA
                                                                       ************************************************
3´ end          CACAATCTACATTGTAAAAGCGCTATACAAATAAAGCTGAATTGAATTGAATTGAATTAATTATGACAAGGCTGTTAAGAAGGATCAAAAGCAGAGAGGAGAGA
```

Ra232

```
5´ end          CAGTAGCATGATCTCAGCTCACTGCAACCTCTGGTCCATGCTTTTATGACTTCGAGACTGGATTACTGTAATGCTCTATTT
                *********************************
hs_chr15 +      CAGTAGCATGATCTCAGCTCACTGCAACCTCTGCCTCCAGGGTTCAAGTGATTCTTTTGCCCTAGCCTCCTGAGTAGCTGT
                                                          ***************************************
3´ end          AAGCGCTATACAAATAAAGCTGAATTGAAAAAAAAAATTGAATTCAAGTGATTCTTTTGCCCTAGCCTCCTGAGTAGCTGT
```

Ra501

```
5´ end          GGTGCATGCCACCAAGCCCAGCTAATTTTTGTATTTTTAGTAGATTTTTATTTAACACCATCTCTAAATTAGCAAATAATCGGTCATCCTTG
                ********************************************
ucsc_chr8 +     GGTGCATGCCACCAAGCCCAGCTAATTTTTGTATTTTTAGTAGAGACGGGGTTTTGCCATGTTGGTCAGGCTGAACTCCTGACCTCAGGTGA
                                                        ******* ********************************************
3´ end          AAAGCGCTATACAAATAAAGCTGAATTGAATTGAATTGAATAGAGACCGGGTTTTGCCATGTTGGTCAGGCTGAACTCCTGACCTCAGGTGA
```

Ra506

```
5´ end          GCAATCTACTCGTCTGACAAAGGACTAATATCCAGAATCTACAATGAACTCAAATCTCCGGCAGTCGGAGATCCTGAAGAAGGACTTCAGGAGCCTGA
                *******************************************************
UCSC_hs_chr6 +  GCAATCTACTCGTCTGACAAAGGACTAATATCCAGAATCTACAATGAACTCAAATTTACAAGGAAAAAATAAACAACCCCATCAAAAAGTGGGCAAAG
                                                                   ***********************************************
3´ end          ATTGTAAAAGCGCTATACAAATAAAGCTGAATTGAATTGAATTGAATTGAAAAATTTACAAGGAAAAAATAAACAACCCCATCAAAAAGTGGGCAAAG
```

Ra507

```
5´ end          GGGAAAGCTTTCATTCAAGTTTCTCTCTTAGGTACTCTGCTAGCTTTATTCTCTATTTTCACCTGGGGATACTCAT
                    ***************************************
hs_chr5 −       GAAGAAGCTTTCATTCAAGTTTCTCTCTTAGGTACTCTGCTAGATGTACAGCAAGAAAAGAACTCTGCCTTCAGGT
                                                        ************************************
3´ end          GCTATACAAATAAAGCTGAATTGAATTGAATTGAATTGAATAGATGTACAGCAAGAAAAGAACTCTGCCTTCAGGT
```

Ra510

```
5´ end          CCAGCTAATTTATTTTATTTTTTTTGTAGAGATCGGGTCTCATTATGTTAAATCGCTTAAAGTCTACAGGTGTCCAGGGACAGGCTCTA
                *************************************************
UCSC_chr5 +     CCAGCTAATTTATTTTATTTTTTTTGTAGAGATCGGGTCTCATTATGTTGCCCAGGCTAGTCTGGAACTCCTGGCCTTAAGCCAACCTC
                                                            *********************************************
3´ end          AAAGCGCTATACAAATAAAGCTGAATTGAATTGAATTGAATTGAATGTTGCCCAGGCTAGTCTGGAACTCCTGGCCTTAAGCCAACCTC
```

Ra511

```
5´ end          ATCTCATTGTATTGCCCAAGCTGGTTTCGAACTCCTGGCCTCAAGCGATCCTCTTTAGCTCTACTATAAAAGATTTGGGTGTCATATTAGACAGCAA
                *******************************************************
UCSC_chr19 −    ATCTCATTGTATTGCCCAAGCTGGTTTCGAACTCCTGGCCTCAAGCGATCCTCTTGCCCTGGCCTTCCAAAGTGCTGGGACTACCGGTAGGAGCCAC
                                                                   **********************************************
3´ end          TCTACATTGTAAAAGCGCTATACAAATAAAGCTGAATTGAATTGAATTGAATCTTGCCCTGGCCTTCCAAAGTGCTGGGACTACCGGTAGGAGCCAC
```

Ra512

```
5´ end          CGTGCCTGTCTTCAGATCTTCACAGCACAGTTCCTGGGAAGGAGCTGGGTACTAGCGGCCGCCACCATG
                ******************************************
hs_chr2 −       CGTGCCTGTCTTCAGATCTTCACAGCACAGTTCCTGGGAAGGTGGAGCCACCAGCCTCTCCCTGGTGAG
                                                     ********************************
3´ end          AAAGCGCTATACAAATAAAGCTGAATTGAATTGAATTGAAGGTGGAGCCACCAGCCTCTCCCTGGTGAG
```

Ra513

```
5´ end          TTCAAAAAAAAGTTTCTTTCCCTTGGAGTTTATGGTTACATTAAAAATCATGCTATCCATCTCAGATGCAGAAAAGCTAGTCCATGCT
                ***************************************************
hs_chr1 +       TTCAAAAAAAAGTTTCTTTCCCTTGGAGTTTATGGTTACATTAAAAATCATATACCCTCTTGGATTCACACAAAAACCTGATTTCAAG
                                                          **********************************************
3´ end          CATTGTAAAAGCGCTATACAAATAAAGCTGAATTGAATTGAAAAAAATCATATACCCTCTTGGATTCACACAAAAACCTGATTTCAAG
```

Ra514

```
5´ end        CTCCCAAAACATTTTTTTTTAATTAACCAGACATAATGACACGTACGCTAATTATTCTCTTTATTCTCTATTTTCACCTGGGGA
              **********************************************
hs_chr21 +    CTCCCAAAACATTTTTTTTTAATTAACCAGACATAATGACACGTACCTGTGGTCCCAGCTGCTTGGAAAGCGGAGGTAAGAGGA
                                                          ****************************************
3´ end         TTGTAAAAGCGCTATACAAATAAAGCTGAATTGAATTGAATTGAACCTGTGGTCCCAGCTGCTTGGAAAGCGGAGGTAAGAGGA
```

Ra515

```
5´ end        AGCAGCTCTGCTGGACTCTCAGCTTGCTGAATAGTTCGCACCATCTGACTAGCAGGTAAAAATTCACAAAATTCACACTATAGCC
              ************************************
hs_chr3 +     AGCAGCTCTGCTGGACTCTCAGCTTGCTGAATAGTTAGAAAAAGGGAAAAAACCCACACTATTCTAAGCTAGTATGTACAACCCA
                                                           ****************************************
3´ end        AGCGCTATACAAATAAAGCTGAATTGAATTGAATTGAATTGAATTGAAAAAACCCACACTATTCTAAGCTAGTATGTACAACCCA
```

Ra701

```
5´ end        AACATCATGTTATACACCATAAATATATATAATTTTGCCCATTTACATCTTGCTTATATACACTATGAACAGCAGNTACGC
              *********************************************
UCSC_chr4 –   AACATCATGTTATACACCATAAATATATATAATTTTGCCCATTTAAAAAAATAAATAAATTTTTTAAAAGAAACCGTAAGT
                                                      *****************************************
3´ end        CGCTATACAAATAAAGCTGAATTGAATTGAATTGAATTGAATTTAAAAAAATAAATAAATTTTTTAAAAGAAACCGTAAGT
```

Ra705

```
5´ end        TGAATTAATCCCTATATTCAATTCAAAATAAAAAATAAATTAAAATAATAAGTAATAAAAACAGAATTATTACTTAT
              *****************
hs_chr3 –     TGAATTAATCCCTATATCAATTGTATGCATTCATATATTCATTTTTACAACAGATAGATACTAAGCATCTACCATGT
                              3262766
                                                         1539076
hs_chr3 –     TTTTATGATCTAAGCACACCATAAAACTTGTCAGGAAATGCAGATCAAGGATGGTGCCTCTAATTCAACTGCAATCC
                                                         ************************************
3´ end        AAAGCGCTATACAAATAAAGCTGAATTGAATTGAATTGAAAAGATCAAGGATGGTGCCTCTAATTCAACTGCAATCC
```

Ra707

```
5´ end          TGTCCTGACCACACCAGCCTCTTTGCTGATCCCTTGACACAGATCTCGCAACTCAATTTACAATTAGAGGGATACAAAGTTAG
                *****************************************
hs_chr20 −      TGTCCTGACCACACCAGCCTCTTTGCTGATCCCTTGACACACTCAAGCTCCTGCCTCCCAGCCTCTGATCTGCTCATCCCTCT
                                                        *******************************************
3´ end          CTATACAAATAAAGCTGAATTGAAAAAAAAAAAAAAAAAAACTCAAGCTCCTGCCTCCCAGCCTCTGATCTGCTCATCCCTCT
```

Ra708

```
5´ end          TGGTTTGAAACTTTTTTGATATACTGGGTTCATCCTCTGGAGTCCTAAAACCATATATCCCATGTCACAAAAACTGCCTTCTTTC
                ************************************************
hs_chr1 +       TGGTTTGAAACTTTTTTGATATACTGGGTTCATCCTCTGGAGTCCTAACAATGTTTTAGCTAATTTACAAAAAACAAAACAAAAC
                                                          *******************************************
3´ end          AAAAGCGCTATACAAATAAAGCTGAACTGAATTGAAAAAAAATCCTAACAATGTTTTAGCTAATTTACAAAAAACAAAACAAAAC
```

Nimb2 / DT40 WT cells

pWG2.3a

```
5´ end          TAAAGAAATGGTTCCTCCTGCTTTCATAAACAGTCATGTGCAGAGTTCGGCAGGGCCGAGATCTCTAGAAGCTGGGTACCAGCTGCTAGCGGAGGA
                *********************************************************
gg_chr1 -       TAAAGAAATGGTTCCTCCTGCTTTCATAAACAGTCATGTGCAGAGTTCGGCAGGGCCTGATGCCCCCGGAGCTCGTTCCTTCCCAGCCCGATTGAA
                                                           *****************************************************
3´ end          GTTGCCAACCGCCAGTAAAACCCAAAGAAGAAGAAGAAGAAGAAGTTCGGCAGGGCCTGATGCCCCCGGAGCTCGTTCCTTCCCAGCCCGATTGAA
```

pWG2.4a

```
5´ end          GACCACCACCTGCACACCACATCCGGGCCTACAAGAACGGAGTCACGGGTTAGAACCAGTACACTGGGATAACATTGATACAGGTAGACATTTATATAATATAC
                **********************************************************
gg_chr1 +       GACCACCACCTGCACACCACATCCGGGCCTACAAGAACGGAGTCACGGGTTAGAACCACAGAACCATTCAGGCTGGAAAAGACCTCAGATATCACCCGGTCCAA
                                                                   *****************************************************
3´ end          TAAGTCTGGTTGCCAACCGCCAGTAAAACCCAAAGAAGAAGAAGAAGAAGAAGAACCACAGAACCATTCAGGCTGGAAAAGACCTCAGATATCACCCGGTCCAA
```

pWG3.1a

```
5´ end          TGGCAAGTTCAGTGAGACTTTGTGGAGAGGAGTATGATTATCGTAGGGAATCGTATGAAATCGTAGGGAAGGAAGTATTATAACTCG
                *****************************************
UCSC_gg_chr12 + TGGCAAGTTCAGTGAGACTTTGTGGAGAGGAGTATGATTATGGCAGGGACAGGAGGGATTAGCATTGCACTCCTGCATCTAAATAAT
                                              *********************************************************
3´ end          ACCGCCAGTAAAACCCAAAGAAGAAGAAGAAGTATGATTATGGCAGGGACAGGAGGGATTAGCATTGCACTCCTGCATCTAAATAAT
```

pWG3.2a

```
5´ end          AAGTTTGCATATGTGCTCTAAGAAGAGGGTGCAAAGTTGAAATGTTGCAACTGTGGAGGGGATCATAGCTCAGCGT
                *************************
gg_chr5 +       AAGTTTGCATATGTGCTCTAAGAAGTTTCTTAACTGTGTATTGAATGTTTCACTCCTGGAGATCGGAGCAGCTTCC
                                                              ******************************
3´ end          GTCTGGTTGCCAACCGCCAGTAAAACCCAAAGAAGAAGAAGAAGAAGTTTCACTCCTGGAGATCGGAGCAGCTTCC
```

pWG3.3a

```
5´ end          TCTTAGAAGAAGAAAAGTGAGAAAACACAGAATTAGTCATAGAGTCATAGAATCATACCCAGTTAGAGGAAAGAATATAGAGAGCGGCCGCG
                *********************************************************
UCSC_gg_chr2 -  TCTTAGAAGAAGAAAAGTGAGAAAACACAGAATTAGTCATAGAGTCATAGAATCATAGAATGGCCTGGGTTGAAAAGGACCACAATGATCAT
                                                          **************************************************
3´ end          TGGTTGCCAACCGCCAGTAAAACCCAAAGAAGAAGAAGAAGAAGTCATAGAATCATAGAATGGCCTGGGTTGAAAAGGACCACAATGATCAT
```

pWG4.5a

```
5´ end          TTCATCAAGTCAGAGGTATGCTTCAGCACTTCAGAGAGAGAAACCTCAGCAGCTGCAGGGATAGATCTCTAGAAGCTGGGTACCAGCTGCTAGCGGAGG
                **************************** *********************************
gg_chr20 -      TTCATCAAGTCAGAGGTATGCTTCAGCATTTCAGAGAGAGAAACCTCAGCAGCTGCAGGGATGGGGAGGGACAACAGATTCTTAAGCAGAAAAGAGGAA
                                                                  *************************************************
3´ end          TCTGGTTGCCAACCGCCAGTAAAACCCAAAGAAGAAGAAGAAGAAGAAGAAGCTGCAGGGATGGGGAGGGACAACAGATTCTTAAGCAGAAAAGAGGAA
```

pWG5.1b

```
5´ end          GTTCTGTGCTTTTAGCATGTTTTGTTTTTCCTTTCAGGTAACTTTGAAAAGAGATCTCTAGAAGCTGGGTACCAGCTGCTAGCGGAGGATGGCA
                ***************************************************
gg_chr4 +       GTTCTGTGCTTTTAGCATGTTTTGTTTTTCCTTTCAGGTAACTTTGAAAAGCAGTATGATGATGTGACAATCAAAATGATCTTTGCAATTGTTC
                                                       *******************************************************
3´ end          CGCCAGTAAAACCCAAAGAAGAAGAAGAAGAAGAAGAAGAACTTTGAAAAGCAGTATGATGATGTGACAATCAAAATGATCTTTGCAATTGTTC
```

pWG5.2a

```
5´ end          TGAAAAAAATGTAAATCAGCTTATGTCCCTTTGAGATCTCTAGAAGCTGGGTACCAGCTGCTAGCGGAGGATGGCAAGGCGGA
                *********************************
gg_chr2 -       TGAAAAAAATGTAAATCAGCTTATGTCCCTTTGTCCTATAAAAGGGAACTGAGACTATGAGCATACCTGTGTTTGACTTCAGA
                                                                          *************************
3´ end          CACCTATAAGTCTGGTTGCCAACCGCCAGTAAAACCCAAAGAAGAAGAAGAAGAAGAAGAGCATACCTGTGTTTGACTTCAGA
```

pWG5.3a

```
5´ end        AGCAAGACAGACATGTTGGGAGAAATTTCTGATTCTAGATAGATAACATCTTTCATTTGAAGGGCAAATCAGTATATTTCATTTGGGTTCCTG
              ****************************** **********************
gg_chr1 +     AGCAAGACAGACATGTTGGGAGAAATTTCTTATTCTAGATAGATAACATCTTTGTGTTCATGAATATATGAGCAATATGGTGACATTCCTGCA
                                                      *****************************************************
3´ end        GCCAACCGCCAGTAAAACCCAAAGAAGAAGAAGAAGAAGAAGATAACATCTTTGTGTTCATGAATATATGAGCAATATGGTGACATTCCTGCA
```

pWG5.4a

```
5´ end        AGGTCAAGTCAAAACCTCACAGCACAAAAAATTTCACTTCAGCCCAGTAATCCACCAGTTTCATCTATGCAGGTTGAAATGGGAGAAATGCCCTTAAG
              **********************************************************
gg_chr5 -     AGGTCAAGTCAAAACCTCACAGCACAAAAAATTTCACTTCAGCCCAGTAATCCACCAGGAAACTACAAAGCGCTTTGTCTTTAGTAGAATGTTTACTC
                                                           *****************************************************
3´ end        GTCTGGTTGCCAACCGCCAGTAAAACCCAAAGAAGAAGAAGAAGAAGTAATCCACCAGGAAACTACAAAGCGCTTTGTCTTTAGTAGAATGTTTACTC
```

pWG5.5a

```
5´ end        CTACACCAAAGGCTGGATCAGTACTGCTAATTTGGATCCCAGTAACATACCACAAGAGATTAAGGGATATAATATAGTTAGAAATGATAGGAATC
              *******************************************************
gg_chr2 -     CTACACCAAAGGCTGGATCAGTACTGCTAATTTGGATCCCAGTAACATACCACAAAAGAGAAAGAGGTGCGAAATCCAGTTCACCTTATTGACCC
                                                         ****************************************************
3´ end        CTGGTTGCCAACCGCCAGTAAAACCCAAAGAAGAAGAAGAAGAAACATACCACAAAAGAGAAAGAGGTGCGAAATCCAGTTCACCTTATTGACCC
```

pWG6.3a

```
5´ end        ACCGGCAGCGCGCCTGCGCCGGGCCGAGCGGGGCCGCGAGAGGGCTCGGGCTGCGGGCTGCAGATCTCTAGAAGCTGGGTACCAGCTGCTAGCGGAGGATGGCAAGG
              *************************************************************
gg_chr9 +     ACCGGCAGCGCGCCTGCGCCGGGCCGAGCGGGGCCGCGAGAGGGCTCGGGCTGCGGGCTGCGGCCTGCGGGGCGGCCGGCCCACACATCCTGCACGCCCTCTGCTGA
                                                              ***********************************************************
3´ end        TCTGGTTGCCAACCGCCAGTAAAACCCAAAGAAGAAGAAGAAGAAGAAGGCTGCGGGCTGCGGCCTGCGGGGCGGCCGGCCCACACATCCTGCACGCCCTCTGCTGA
```

```
pWG7.2a
5´ end      AGCACTCAACAATGCCATACAGGATCTTGTAAATGTAAATGACAAAAATGTAAATCATGTTAAACGTTCTACAATGGAATGCAAGGAGCTTAATTGCGAAT
            **********************************************************
gg_chr3 +   AGCACTCAACAATGCCATACAGGATCTTGTAAATGTAAATGACAAAAATGTAAATCATTTTTGTCTAGGAAGACAGCCTATCTACGATTGATAATTTCTTC
                                                        *********************************************************
3´ end      TTGCCAACCGCCAGTAAAACCCAAAGAAGAAGAAGAAGAAGAAGAAAATGTAAATCATTTTTGTCTAGGAAGACAGCCTATCTACGATTGATAATTTCTTC

pWG7.5a
5´ end      GCAATAATGGTAGAAAGTGCTTGTACTTCTGCATTTTTTCTCAGGCCAGTTTCTTCCAGGAGATCTCTAGAAGCTGGGTACCAGCTGCTAGCGGAGGATGGCAA
            ************************************************************
gg_chr2 -   GCAATAATGGTAGAAAGTGCTTGTACTTCTGCATTTTTTCTCAGGCCAGTTTCTTCCAGGGCTCTTCTTTATGCCAAGCATCCTTTAAACTTGAAGGCTATTGT
                                                           *********************************************************
3´ end      AAGTCTGGTTGCCAACCGCCAGTAAAACCCAAAGAAGAAGAAGAAGAAGTTTCTTCCAGGGCTCTTCTTTATGCCAAGCATCCTTTAAACTTGAAGGCTATTGT

pWG7.6a
5´ end      GCGGGGCTGCCCCGGGGCGGGGGTGGCGGGGACTATAAAGAGCCGCGTCCCGATTACACCCAACTGGGCAATGTATACATTGTAATCAACAAGAAACAATAGAACATATT
            ****************************************************
gg_chr7 -   GCGGGGCTGCCCCGGGGCGGGGGTGGCGGGGACTATAAAGAGCCGCGTCCCGGGCGGGAGCGGGGCTGTGCGCTCGGTGCAGCTGCGCTCGTGGCGCTCGGACGGCCCGG
                                                                   *******************************************************
3´ end      CTATAAGTCTGGTTGCCAACCGCCAGTAAAACCCAAAGAAGAAGAAGAAGAAGAAGGGAGCGGGGCTGTGCGCTCGGTGCAGCTGCGCTCGTGGCGCTCGGACGGCCCGG

pWG8.1a
5´ end      GCAATATTGGACAGAAAATACGTGACATAGCAATTTGCAGTGAGTTGAGAACTGTATAGAGATCTCTAGAAGCTGGGTACCAGCTGCTAGCGGAGGATGG
            **********************************************************
gg_chr2 -   GCAATATTGGACAGAAAATACGTGACATAGCAATTTGCAGTGAGTTGAGAACTGTATATCAAATAGACTGTTACAGACTGAAAGGTGCGAACAGCTTTGT
                                                          ******************************************************
3´ end      TTGCCAACCGCCAGTAAAACCCAAAGAAGAAGAAGAAGAAGAAGAAGAGAACTGTATATCAAATAGACTGTTACAGACTGAAAGGTGCGAACAGCTTTGT
```

pWG8.2a

```
5´ end      TTCTCCAGCTTATTGAAATCCATGCAATTATTGACCTTCTTCTCCATTAATCCATCACTACAAAATATATACTGGGCCCTCATGAGATCTGTTTTTGATTATGGAT
            ******************************************************
gg_chr2 -   TTCTCCAGCTTATTGAAATCCATGCAATTATTGACCTTCTTCTCCATTAATCCAAAAAAACCTGAATGTCTACTTTAGGAGACATTCCTGACATTTATCTTCTTAA
                                                                      ************************************************
3´ end      TGCACCTATAAGTCTGGTTGCCAACCGCCAGTAAAACCCAAAGAAGAAGAAGAAGAAGAACCTGAATGTCTACTTTAGGAGACATTCCTGACATTTATCTTCTTAA
```

pWG8.3a

```
5´ end      AGGGCTTCTGAACTCTGCCACAAACAAAACTGGGAAGGTCTATTGTGGTCACACTGGACACTGGACTCAATCATACATTACATAAAATAGG
            ***********************************************
gg_chr4 +   AGGGCTTCTGAACTCTGCCACAAACAAAACTGGGAAGGTCTATTGTGACTTCTGATAAGAACATTTATTTTACCTCAATTAAATTTCAGGC
                                             **********************************************************
3´ end      CAACCGCCAGTAAAACCCAAAGAAGAAGAAGAAGAAGGTCTATTGTGACTTCTGATAAGAACATTTATTTTACCTCAATTAAATTTCAGGC
```

pWG9.2a

```
5´ end      TATCAAGCATTGCACAGGTAGTCATTTATTCCCAAGTGGATTTATCTTTCTTTGCAAGAATGGGCAAATAAATGGGGATTCAAACTATCTGTTGCAAAAACTCAGGTCATCT
            *************************************************************
gg_chr3 -   TATCAAGCATTGCACAGGTAGTCATTTATTCCCAAGTGGATTTATCTTTCTTTGCAAGAATTCCTCTGGATAGAAGCATAGAAGTTTTGAGTTTTCTTTCCCCTAAAAACAA
                                                               *********************************************************
3´ end      ACCTATAAGTCTGGTTGCCAACCGCCAGTAAAACCCAAAGAAGAAGAAGAAGAAGAAGAATTCCTCTGGATAGAAGCATAGAAGTTTTGAGTTTTCTTTCCCCTAAAAACAA
```

pWG9.3a

```
5´ end      CTGGTGGATGAAAAGCTGGACATGAGCCAGCAGTATGTGCCTATAGCCTGGAAGGTCAACAGGAAAGAATATAGAGAGCGGCCGCGCACAAACGACCCAACACCCGTGC
            **************************************************************
gg_cbr3 +   CTGGTGGATGAAAAGCTGGACATGAGCCAGCAGTATGTGCCTATAGCCTGGAAGGTCAACAGTATTTTGGGCAGCATCAAAAGAGGGATGGCGGGAGGGTGAGGGAGGT
                                                              ***********************************************************
3´ end      ATAAGTCTGGTTGCCAACCGCCAGTAAAACCCAAAGAAGAAGAAGAAGAAGGTCAACAGTATTTTGGGCAGCATCAAAAGAGGGATGGCGGGAGGGTGAGGGAGGT
```

pWG10. 1a

```
5´ end          GGATGATCCCGCGGGTCTTTTCCAACCTTAGCGATTCTATGATTCTATGATAAGCCATTAGATCTCTAGAAGCTGGGTACCAGCTGCTAGCGGAGGATGGCAAGGC
                ******************************************** ***************
gg_chr4 -       GGATGATCCCGCGGGTCTTTTCCAACCTTAGCGATTCTATGATTTTATGATAAGCCATTAGGTAGCTATGAAAAAGTTCATGTTACAAGCCATGCTGATTTTGACT
                                                                ************************************************************************************************************
3´ end          ACCTATAAGTCTGGTTGCCAACCGCCAGTAAAACCCAAAGAAGAAGAAGATAAGCCATTAGGTAGCTATGAAAAAGTTCATGTTACAAGCCATGCTGATTTTGACT
```

pWG10. 3a

```
5´ end          AATAGGAGCGCGGGAGGGTATTTAAAAGCAACCGCGCGGCGCGAGGCAGCAGTTGAGATCTCTAGAAGCTGGGTACCAGCTGCTAGCGGAGGATGGCAAG
                ******************************************************
gg_chr10 -      AATAGGAGCGCGGGAGGGTATTTAAAAGCAACCGCGCGGCGCGAGGCAGCAGTTGGTGGTCTTTCTCGCGGCCGAGGCGTGTTTGTGTAGAGCGGCGCAA
                                                          **********************************************************
3´ end          GGTTGCCAACCGCCAGTAAAACCCAAAGAAGAAGAAGAAGAAGAGGCAGCAGTTGGTGGTCTTTCTCGCGGCCGAGGCGTGTTTGTGTAGAGCGGCGCAA
```

pWG11. 2a

```
5´ end          TGCTAAATGGCCAAAGAGGTAAAACACGTTGCGTTTGCTATAGATGAAGTCAATCAGTATATCAGAGTGGGTTCCGCAGAGGCAGAAATACTATGGACTCAG
                ***********************************************************
gg_chr1 -       TGCTAAATGGCCAAAGAGGTAAAACACGTTGCGTTTGCTATAGATGAAGTCAATCAGTACATAGTTTGTTGTCTTACGGGTTTATAAGCTAGGACAGAAGCA
                                                             *********************************************************
3´ end          TCTGGTTGCCAACCGCCAGTAAAACCCAAAGAAGAAGAAGAAGAAGAAGTCAATCAGTACATAGTTTGTTGTCTTACGGGTTTATAAGCTAGGACAGAAGCA
```

pWG12. 2a

```
5´ end          CTATTATTGCTAGCCTTTATGTTATTTCAGTCACGATGGAGAAAAGCACTGATGGAGATCAGCATTTTACATTGGCAGGTTTATTAGGAAACA
                ****************************************************
gg_chr12 +      CTATTATTGCTAGCCTTTATGTTATTTCAGTCACGATGGAGAAAAGCACTGACAGACTCTGCTGGCTTTGCAGGGGCTGTGATGCCCCATCCC
                                                      ********************* *********************************
3´ end          GCCAACCGCCAGTAAAACCCAAAGAAGAAGAAGAAGAAGAGAAAAGCACTGACAGACTCCGCTGGCTTTGCAGGGGCTGTGATGCCCCATCCC
```

pWG13. 1a

```
5´ end          AGACTTTGAAAAAATCCTTGTAATTGCTGTGACACTGACGAAGTAATCTAATAAAAAAGGTTCCAGAAATTATGACTGTGTCTAAACAAAATGAAAACAGGAA
                ************* ****************************************
gg_chr13 +      AGACTTTGAAAAACTCCTTGTAATTGCTGTGACACTGACGAAGTAATCTAATAATTGGTTGCTGAAGTGTTGTTACCCACAGGAGTGTTAGTCACGGGTTGAG
                                                                    ***************************************************
3´ end          ACCTATAAGTCTGGTTGCCAACCGCCAGTAAAACCCAAAGAAGAAGAAGAAGAATTGGTTGCTGAAGTGTTGTTACCCACAGGAGTGTTAGTCACGGGTTGAG
```

pWG13. 2a

```
5´ end          GCCCGGCGCGCTCCGTCGTTTTAAAGCCTCCCGCCGTCGCTGCTCCCCGGCTGCAGATCTCTAGAAGCTGGGTACCAGCTGCTAGCGGAGGATGGCAA
                ************************************************** **
gg_chr18 –      GCCCGGCGCGCTCCGTCGTTTTAAAGCCTCCCGCCGTCGCTGCTCCCCGGTTGTGCTCGGCCGCGTCCCGCCCGGATCGGGGTTCTTTGGCTCAGGCC
                                                        ********** ************************ **********************
3´ end          GCCAACCGCCAGTAAAACCCAAAGAAGAAGAAGAAGAAGATGCTCCCCGGCTGTGCTCGGCCGCGTCCCGCCCGGCTCGGGGTTCTTTGGCTCAGGCC
```

pWG14. 1a

```
5´ end          GCGCGGGGCCCGCCCGCTATATGAGCTCCGCCCGGCGCTGACTCGGCCCTTGAGATCTCTAGAAGCTGGGTACCAGCTGCTAGCGGAGGATGGCAAGG
                ***************************************************
UCSC_gg_chr1 –  GCGCGGGGCCCGCCCGCTATATGAGCTCCGCCCGGCGCTGACTCGGCCCTTTCCGCCTCGCTCCCGCCCGCTCTCGGGTCGCTGCGCAGAGAGCCGCG
                                                       ***********************************************************
3´ end          CAACCGCCAGTAAAACCCAAAGAAGAAGAAGAAGAAGAAGACTCGGCCCTTTCCGCCTCGCTCCCGCCCGCTCTCGGGTCGCTGCGCAGAGAGCCGCG
```

pWG14. 2a

```
5´ end          ATGCTACCATGCCAAAAGCTTCTACCAGAAACCTTGGATGTTTTACAAGAAGGAAACATCTATGCAGGTTGAAATGGGAGAAATGCCCTTAAGCATTAGGAG
                              *********************************************
gg_chr2 –       GCAACTTTTGTAATAAAGCTTCTACCAGAAACCTTGGATGTTTTACAAGAAGGAAACATTGGCTAAGGCTGCAACATAAAAATGTGGTATATGATTCCCATA
                                                              ********************************************************
3´ end          AAGTCTGGTTGCCAACCGCCAGTAAAACCCAAAGAAGAAGAAGAAGAAGAAGGAAACATTGGCTAAGGCTGCAACATAAAAATGTGGTATATGATTCCCATA
```

pWG15.1a

```
5´ end          CTGAAGGGTGGGCAGGGAGGGATGGATATGAAAGCTGAGGGTTTTTTTGTTGGCAGACAAAAGAGCAATCTTCAGCATTTTACATTGGCAGGTTTATTAGGAAACAAATCTA
                *************************************************************
gg_chr15 +      CTGAAGGGTGGGCAGGGAGGGATGGATATGAAAGCTGAGGGTTTTTTTGTTGGCAGACAAATAGGTATTGTTACAAAATTCGGAAAGCTTCCCACTGCTGATAGGCACTGGC
                                                                          *********************************
3´ end          TGCACCTATAAGTCTGGTTGCCAACCGCCAGTAAAACCCAAAGAAGAAGAAGAAAAAAAAATAGGTATTGTTACAAAATTCGGAAAGCTTCAAAGTAATTTCAGTGTTTTAT
```

pWG16.1a

```
5´ end          ACCTGCACTGCTCCAGTTTTGAGAGGTCGTGAGG-TTTTTTTAATCCAGAATAAGTTTGGCAAGTACACTGGGATAACATTGATACAGGTAGACATTTATAT
                ********************************** **********************
gg_chr8 +       ACCTGCACTGCTCCAGTTTTGAGAGGTCGTGAGGTTTTTTTTAATCCAGAATAAGTTCCTTTTAAAATAAATATGGGATGTAGGTAATGGGGTTGTACGGGA
                                                                **************************************************
3´ end          TATAAGTCTGGTTGCCAACCGCCAGTAAAACCCAAAGAAGAAGAAGAAGAAGAAGTTCCTTTTAAAATAAATATGGGATGTAGGTAATGGGGTTGTACGGGA
```

L1.3 inversion/ HeLa-RC cells

pHLE2.1a

```
5´ end          GCATTTATCTGGTACTATTTGTTTTGTAGGTTTTAGTTTAGAAAGTCAGACTGCATAAATGTCTTCTTTTGAGAAGTGTCTGTTCATGTCCT
                ****************************************************
hs_chr5 -       GCATTTATCTGGTACTATTTGTTTTGTAGGTTTTAGTTTAGAAAGTCAGACTGAAGTACTAAAAGTTGTCTCATGCGTCCGTGTGAAGAGAC
                                                       *****************************************************
3´ end          AAAAAAAAAAAAAAAAAAAAAAAAAAAAAAAAAAAAAAAAGAAAGTCAGACTGAAGTACTAAAAGTTGTCTCATGCGTCCGTGTGAAGAGAC
```

pHLE3.8a

```
5´ end          CAACATAAGCCATAAATGCAAGCCGCAGAAGTACATGTTAAAATAACCAAAAGAGAAGTGTCTGTTCATGTCCTTCGCCCACTTTTTGATGGGGTTGT
                ******************************************************
hs_chr9 -       CAACATAAGCCATAAATGCAAGCCGCAGAAGTACATGTTAAAATAACCAAAAGAAACAGGTGACCTTCCTTTAACTAATATATTTCATTTAACCCAGT
                                                      ************************************************************
3´ end          AAAAAAAAAAAAAAAAAAAAAAAAAAAAAAAAAAAAAATAAAATAACCAAAAGAAACAGGTGACCTTCCTTTAACTAATATATTTCATTTAACCCAGT
```

pHLE3.11a

```
5´ end          TTAGCAACACATCTGGAATTCCAGAAAATTAGAATGTGACAGAAGAGGCATGTAGGTTTCCTGAATACAGCACACTGATGGGTCTTGACTCTTT
                *********************************************************
hs_chr7 +       TTAGCAACACATCTGGAATTCCAGAAAATTAGAATGTGACAGAAGAGGCATGTAGGTACTCAGGAGTAGTCACAAGGAAAAACTCATAGCTAGA
                                                        ******************************************************
3´ end          AAAAAAAAAAAAAAAAAAAAAAAAAAAAAAAAAAAAAAAAAGAAGAGGCATGTAGGTACTCAGGAGTAGTCACAAGGAAAAACTCATAGCTAGA
```

pHLE4.2a

```
5´ end          CAGAATTTGGGAGACTCAAAAAATCAGCATAAATTGTAAGGGATTGTACAGATGGGTTTTCGGTGTAGATGTCCTTTCTGGTTGTTAGTTT
                *********************************************
hs_chr8 +       CAGAATTTGGGAGACTCAAAAAATCAGCATAAATTGTAAGGGATTTTCAAGAAGACTGACTTAGGCCTAAGCTCATCATTGCACAATGCTT
                              *************************************************************
3´ end          AAAAAAAAAAAAAAAAAAAAAAAAAAAAAAAAATTGTAAGGGATTTTCAAGAAGACTGACTTAGGCCTAAGCTCATCATTGCACAATGCTT
```

pHLE4. 5a

```
5´ end      CTTTGAGATTCAGTGACAATAGAGTCCAATTTAAAAACTACTTGGAAACAAAAAAACCACCGCTACCAGCGGTGGTTTGTTTGCCGGATCAA
            *********************************************
hs_chr5 –   CTTTGAGATTCAGTGACAATAGAGTCCAATTTAAAAACTACTTGGGAAAAAAATATTTGAAATTTCTCACTTGAAAACATAACTCTTCTTTA
                                           *************************************************************
3´ end      AAAAAAAAAAAAAAAAAAAAAAAAAAAAAAATAAAAACTACTTGGGAAAAAAATATTTGAAATTTCTCACTTGAAAACATAACTCTTCTTTA
```

pHLE2. 6a

```
5´ end      GCCACTACGGAAAACAGTGTGGAGATTCCTTAAAGAACTAAAAGTAGAGGAATGTATCCATTTCTTCTAGATTTTCTAGTTTATTTG
            ************************************************
hs_chr16 +  GCCACTACGGAAAACAGTGTGGAGATTCCTTAAAGAACTAAAAGTAGAACTACCATTTGATCCAGCAATCCCACTACTGGGTATCCA
                                           ********************************************************
3´ end      AAAAAAAAAAAAAAAAAAAAAAAAAAAAAAAAAAGAACTAAAAGTAGAACTACCATTTGATCCAGCAATCCCACTACTGGGTATCCA
```

pHLE4. 9a

```
5´ end      ACTTTACAAGAGGTCTTTAAGGGAGTGCTAAACATGAAAATGAAAGACCATATGTATACATGTGCCATGCTGGTGCGCTGCACCCACTAATGT
            ***************************************************
hs_chr2 –   ACTTTACAAGAGGTCTTTAAGGGAGTGCTAAACATGAAAATGAAAGACCATTACTGGCCACCAAAAAAACAAACAAACAAACAAACTTAAATA
                                                     ****************************************************
3´ end      AAAAAAAAAAAAAAAAAAAAAAAAAAAAAAAAAAAAAAAAAGAAAGACCATTACTGGCCACCAAAAAAACAAACAAACAAACAAACTTAAATA
```

pHLE5. 16a

```
5´ end      GGTTTGTCTAAAAATTTGGAGTAAGCAGGAAGGAATGTTTTAAGTTAAGATAAGGATAACCGTATTACCGCCATGCATCTGGAATTCGGCTTACGG
            ********************************************************
hs_chrX +   GGTTTGTCTAAAAATTTGGAGTAAGCAGGAAGGAATGTTTTAAGTTAAGATAAGGAAGTCTGTTAATCAATACACTGGGTCAGAGTGACCTGTAGG
                                                     *******************************************************
3´ end      AAAAAAAAAAAAAAAAAAAAAAAAAAAAAAAAAAAAAAAAAAAGTTAAGATAAGGAAGTCTGTTAATCAATACACTGGGTCAGAGTGACCTGTAGG
```

pHLE6. 19a

```
5´ end          TCTCACACTGTTAAACCTTCCTTTTGATTGAGAAGTTTTGAAAATCTCTTTTTTGGAGGCCTAGGCTTTTGCAACGCGTCTGCAGCACG
                *****************************************************
hs_chr11 -      TCTCACACTGTTAAACCTTCCTTTTGATTGAGAAGTTTTGAAAATCTCTTTTTATAGAATCTCCCAATGGATATTTTTGAGGGCTTTGA
                                                       **************************************************
3´ end          AAAAAAAAAAAAAAAAAAAAAAAAAAAAAAAAAAAAAAAGAAAATCTCTTTTTATAGAATCTCCCAATGGATATTTTTGAGGGCTTTGA
```

pHLE7. 8a

```
5´ end          ATTTCGCAGCATACAAACTGGTCTAAGTGAACATATAAAAATAGAAATTGCTCACATGTTCTTTCCTGCGTTATCCCCTGATTC
                ****************************************************
hs_chr5 -       ATTTCGCAGCATACAAACTGGTCTAAGTGAACATATAAAAATAGAAATTGCTTCAAGGCTGAATATAATCCTGATATAGCAGTC
                                                  **************************************************
3´ end          AAAAAAAAAAAAAAAAAAAAAAAAAAAAAAAAAAATAAAAATAGAAATTGCTTCAAGGCTGAATATAATCCTGATATAGCAGTC
```

pHLE7. 20b

```
5´ end          CATTCAGCCATACCTAGAATGTCAAATGGTCTCTCTTTAACTGGAATGAAAATAGAGGTTTTACTTGCTTTAAAAAACCTCCCACACCTCCCCCTG
                ****************************************************
hs_chr5 +       CATTCAGCCATACCTAGAATGTCAAATGGTCTCTCTTTAACTGGAATGAAAAGAGATGAAAGAACTGTGTGACCAGGAGGGCACTGTAACCTATGA
                                                               *************************************************
3´ end          AAAAAAAAAAAAAAAAAAAAAAAAAAAAAAAAAAAAAAAAAAAAAAAGAAAAGAGATGAAAGAACTGTGTGACCAGGAGGGCACTGTAACCTATGA
```

pHLE8. 12a

```
5´ end          TCAATCGAGGTAAAAATAAAAAGAATAAATTAACAAATAAACAAATATATTTGAAGTCAGGTAGTGTGATGCCTCCAGC
                ***************************************************
hs_chr11 +      TCAATCGAGGTAAAAATAAAAAGAATAAATTAACAAATAAACAAATATATTGATAGATTAATAATAGAAAGATATACCT
                                                      *****************************************
3´ end          AAAAAAAAAAAAAAAAAAAAAAAAAAAAAAAAAAAAAAAAACAAATATATTGATAGATTAATAATAGAAAGATATACCT
```

pHLE8. 25a

```
5´ end          ATACAGGACCCCATTCTTGTGGGCACTGGATGAAAAGACATGGGTTTAAAACTTCATTTTTTAATTTAAAAGGATCTAGG
                ************************************************
hs_chr5 -       ATACAGGACCCCATTCTTGTGGGCACTGGATGAAAAGACATGGGTTTATCTTGTGCCAAGGCCAAGGCTATTTTAATATT
                                               *************************************************
3´ end          AAAAAAAAAAAAAAAAAAAAAAAAAAAAAAAGAAAAGACATGGGTTTATCTTGTGCCAAGGCCAAGGCTATTTTAATATT
```
