# Supplementary material for: Integrated mechanism for the generation of the 5′ junctions of LINE inserts
Source: Nucleic Acids Res. 2014 Nov 6;42(21):13269–79. doi: 10.1093/nar/gku1067 (PMC4245944; doi:10.1093/nar/gku1067)
Supplement: SUPPLEMENTARY DATA [file supp_gku1067_nar-02442-v-2014-File004.docx]

Supplemental Table S1. The Sequences of pLEmH, TK109.17, and Nb2A3-2

pLEmH

|  | Position |
| --- | --- |
| Multi cloning site of pCEP4 | 1-649 |
| Transcriptional strat | 641 |
| L1.3 5´ UTR | 658-1566 |
| L1.3 ORF1 | 1567-2646 |
| L1.3 ORF2 | 2647-6474 |
| L1.3 3´ UTR | 6475-6640 |
| *mneoI_400_/ColE1* | 6641-9742 |
| SV40 poly A signal | 9743-9969 |
| L1.2 3´ UTR | 9979-10031 |
| Human genomic DNA flanked by L1.2 3´ UTR | 10038-10068 |
| SV40 poly A signal of pCEP4 | 10076-10317 |
| *HindIII* mutations | 641, 1769, 2427 |

GTTGACATTGATTATTGACTAGTTATTAATAGTAATCAATTACGGGGTCATTAGTTCATAGCCCATATATGGAGTTCCGCGTTACATAACTTACGGTAAATGGCCCGCCTGGCTGACCGCCCAACGACCCCCGCCCATTGACGTCAATAATGACGTATGTTCCCATAGTAACGCCAATAGGGACTTTCCATTGACGTCAATGGGTGGAGTATTTACGGTAAACTGCCCACTTGGCAGTACATCAAGTGTATCATATGCCAAGTCCGCCCCCTATTGACGTCAATGACGGTAAATGGCCCGCCTGGCATTATGCCCAGTACATGACCTTACGGGACTTTCCTACTTGGCAGTACATCTACGTATTAGTCATCGCTATTACCATGGTGATGCGGTTTTGGCAGTACACCAATGGGCGTGGATAGCGGTTTGACTCACGGGGATTTCCAAGTCTCCACCCCATTGACGTCAATGGGAGTTTGTTTTGGCACCAAAATCAACGGGACTTTCCAAAATGTCGTAATAACCCCGCCCCGTTGACGCAAATGGGCGGTAGGCGTGTACGGTGGGAGGTCTATATAAGCAGAGCTCGTTTAGTGAACCGT**C**AGATCTCTAGAAGCTGGGTACCAGCTGCTAGCAAGCTAGCTTGCTAGCGGCCGCGGGGGAGGAGCCAAGATGGCCGAATAGGAACAGCTCCGGTCTACAGCTCCCAGCGTGAGCGACGCAGAAGACGGTGATTTCTGCATTTCCATCTGAGGTACCGGGTTCATCTCACTAGGGAGTGCCAGACAGTGGGCGCAGGCCAGTGTGTGTGCGCACCGTGCGCGAGCCGAAGCAGGGCGAGGCATTGCCTCACCTGGGAAGCGCAAGGGGTCAGGGAGTTCCCTTTCTGAGTCAAAGAAAGGGGTGACGGTCGCACCTGGAAAATCGGGTCACTCCCACCCGAATATTGCGCTTTTCAGACCGGCTTAAGAAACGGCGCACCACGAGACTATATCCCACACCTGGCTCGGAGGGTCCTACGCCCACGGAATCTCGCTGATTGCTAGCACAGCAGTCTGAGATCAAACTGCAAGGCGGCAACGAGGCTGGGGGAGGGGCGCCCGCCATTGCCCAGGCTTGCTTAGGTAAACAAAGCAGCCGGGAAGCTCGAACTGGGTGGAGCCCACCACAGCTCAAGGAGGCCTGCCTGCCTCTGTAGGCTCCACCTCTGGGGGCAGGGCACAGACAAACAAAAAGACAGCAGTAACCTCTGCAGACTTAAGTGTCCCTGTCTGACAGCTTTGAAGAGAGCAGTGGTTCTCCCAGCACGCAGCTGGAGATCTGAGAACGGGCAGACAGACTGCCTCCTCAAGTGGGTCCCTGACTCCTGACCCCCGAGCAGCCTAACTGGGAGGCACCCCCCAGCAGGGGCACACTGACACCTCACACGGCAGGGTATTCCAACAGACCTGCAGCTGAGGGTCCTGTCTGTTAGAAGGAAAACTAACAACCAGAAAGGACATCTACACCGAAAACCCATCTGTACATCACCATCATCAAAGACCAAAAGTAGATAAAACCACAAAGATGGGGAAAAAACAGAACAGAAAAACTGGAAACTCTAAAACGCAGAGCGCCTCTCCTCCTCCAAAGGAACGCAGTTCCTCACCAGCAACGGAACAAAGCTGGATGGAGAATGATTTTGACGAGCTGAGAGAAGAAGGCTTCAGACGATCAAATTACTCTGAGCTACGGGAGGACATTCAAACCAAAGGCAAAGAAGTTGAAAACTTTGAAAAAAATTTAGAAGAATGTATAACTAGAATAACCAATACAGAGAAGTGCTTAAAGGAGCTGATGGAGCTGAAAACCAAGGCTCGAGAACTACGTGAAGAATGCAGAAGCCTCAGGAGCCGATGCGATCAACTGGAAGAAAGGGTATCAGCAATGGAAGATGAAATGAATGAAATGAAGCGAGAAGGGAAGTTTAGAGAAAAAAGAATAAAAAGAAATGAGCAAAGCCTCCAAGAAATATGGGACTATGTGAAAAGACCAAATCTACGTCTGATTGGTGTACCTGAAAGTGATGTGGAGAATGGAACCAAGTTGGAAAACACTCTGCAGGATATTATCCAGGAGAACTTCCCCAATCTAGCAAGGCAGGCCAACGTTCAGATTCAGGAAATACAGAGAACGCCACAAAGATACTCCTCGAGAAGAGCAACTCCAAGACACATAATTGTCAGATTCACCAAAGTTGAAATGAAGGAAAAAATGTTAAGGGCAGCCAGAGAGAAAGGTCGGGTTACCCTCAAAGGAAAGCCCATCAGACTAACAGTGGATCTCTCGGCAGAAACCCTACAAGCCAGAAGAGAGTGGGGGCCAATATTCAACATTCTTAAAGAAAAGAATTTTCAACCCAGAATTTCATATCCAGCCAAACTAAGTTTCATAAGTGAAGGAGAAATAAAATACTTTATAGACAAGCAAATGTTGAGAGATTTTGTCACCACCAGGCCTGCCCTAAAAGAGCTCCTGAAGGAAGCGCTAAACATGGAAAGGAACAACCGGTACCAGCCGCTGCAAAATCATGCCAAAATGTAAAGACCATCGAGACTAGGAAGAAACTGCATCAACTAATGAGCAAAATCACCAGCTAACATCATAATGACAGGATCAAATTCACACATAACAATATTAACTTTAAATATAAATGGACTAAATTCTGCAATTAAAAGACACAGACTGGCAAGTTGGATAAAGAGTCAAGACCCATCAGTGTGCTGTATTCAGGAAACCCATCTCACGTGCAGAGACACACATAGGCTCAAAATAAAAGGATGGAGGAAGATCTACCAAGCCAATGGAAAACAAAAAAAGGCAGGGGTTGCAATCCTAGTCTCTGATAAAACAGACTTTAAACCAACAAAGATCAAAAGAGACAAAGAAGGCCATTACATAATGGTAAAGGGATCAATTCAACAAGAGGAGCTAACTATCCTAAATATTTATGCACCCAATACAGGAGCACCCAGATTCATAAAGCAAGTCCTCAGTGACCTACAAAGAGACTTAGACTCCCACACATTAATAATGGGAGACTTTAACACCCCACTGTCAACATTAGACAGATCAACGAGACAGAAAGTCAACAAGGATACCCAGGAATTGAACTCAGCTCTGCACCAAGCAGACCTAATAGACATCTACAGAACTCTCCACCCCAAATCAACAGAATATACCTTTTTTTCAGCACCACACCACACCTATTCCAAAATTGACCACATAGTTGGAAGTAAAGCTCTCCTCAGCAAATGTAAAAGAACAGAAATTATAACAAACTATCTCTCAGACCACAGTGCAATCAAACTAGAACTCAGGATTAAGAATCTCACTCAAAGCCGCTCAACTACATGGAAACTGAACAACCTGCTCCTGAATGACTACTGGGTACATAACGAAATGAAGGCAGAAATAAAGATGTTCTTTGAAACCAACGAGAACAAAGACACCACATACCAGAATCTCTGGGACGCATTCAAAGCAGTGTGTAGAGGGAAATTTATAGCACTAAATGCCTACAAGAGAAAGCAGGAAAGATCCAAAATTGACACCCTAACATCACAATTAAAAGAACTAGAAAAGCAAGAGCAAACACATTCAAAAGCTAGCAGAAGGCAAGAAATAACTAAAATCAGAGCAGAACTGAAGGAAATAGAGACACAAAAAACCCTTCAAAAAATCAATGAATCCAGGAGCTGGTTTTTTGAAAGGATCAACAAAATTGATAGACCGCTAGCAAGACTAATAAAGAAAAAAAGAGAGAAGAATCAAATAGACACAATAAAAAATGATAAAGGGGATATCACCACCGATCCCACAGAAATACAAACTACCATCAGAGAATACTACAAACACCTCTACGCAAATAAACTAGAAAATCTAGAAGAAATGGATACATTCCTCGACACATACACTCTCCCAAGACTAAACCAGGAAGAAGTTGAATCTCTGAATAGACCAATAACAGGCTCTGAAATTGTGGCAATAATCAATAGTTTACCAACCAAAAAGAGTCCAGGACCAGATGGATTCACAGCCGAATTCTACCAGAGGTACATGGAGGAACTGGTACCATTCCTTCTGAAACTATTCCAATCAATAGAAAAAGAGGGAATCCTCCCTAACTCATTTTATGAGGCCAGCATCATTCTGATACCAAAGCCGGGCAGAGACACAACCAAAAAAGAGAATTTTAGACCAATATCCTTGATGAACATTGATGCAAAAATCCTCAATAAAATACTGGCAAACCGAATCCAGCAGCACATCAAAAAACTTATCCACCATGATCAAGTGGGCTTCATCCCTGGGATGCAAGGCTGGTTCAATATACGCAAATCAATAAATGTAATCCAGCATATAAACAGAGCCAAAGACAAAAACCACATGATTATCTCAATAGATGCAGAAAAAGCCTTTGACAAAATTCAACAACCCTTCATGCTAAAAACTCTCAATAAATTAGGTATTGATGGGACGTATTTCAAAATAATAAGAGCTATCTATGACAAACCCACAGCCAATATCATACTGAATGGGCAAAAACTGGAAGCATTCCCTTTGAAAACCGGCACAAGACAGGGATGCCCTCTCTCACCGCTCCTATTCAACATAGTGTTGGAAGTTCTGGCCAGGGCAATCAGGCAGGAGAAGGAAATAAAGGGTATTCAATTAGGAAAAGAGGAAGTCAAATTGTCCCTGTTTGCAGACGACATGATTGTATATCTAGAAAACCCCATCGTCTCAGCCCAAAATCTCCTTAAGCTGATAAGCAACTTCAGCAAAGTCTCAGGATACAAAATCAATGTACAAAAATCACAAGCATTCTTATACACCAACAACAGACAAACAGAGAGCCAAATCATGGGTGAACTCCCATTCGTAATTGCTTCAAAGAGAATAAAATACCTAGGAATCCAACTTACAAGGGATGTGAAGGACCTCTTCAAGGAGAACTACAAACCACTGCTCAAGGAAATAAAAGAGGACACAAACAAATGGAAGAACATTCCATGCTCATGGGTAGGAAGAATCAATATCGTGAAAATGGCCATACTGCCCAAGGTAATTTACAGATTCAATGCCATCCCCATCAAGCTACCAATGACTTTCTTCACAGAATTGGAAAAAACTACTTTAAAGTTCATATGGAACCAAAAAAGAGCCCGCATTGCCAAGTCAATCCTAAGCCAAAAGAACAAAGCTGGAGGCATCACACTACCTGACTTCAAACTATACTACAAGGCTACAGTAACCAAAACAGCATGGTACTGGTACCAAAACAGAGATATAGATCAATGGAACAGAACAGAGCCCTCAGAAATAATGCCGCATATCTACAACTATCTGATCTTTGACAAACCTGAGAAAAACAAGCAATGGGGAAAGGATTCCCTATTTAATAAATGGTGCTGGGAAAACTGGCTAGCCATATGTAGAAAGCTGAAACTGGACCCCTTCCTTACACCTTATACAAAAATCAATTCAAGATGGATTAAAGATTTAAACGTTAAACCTAAAACCATAAAAACCCTAGAAGAAAACCTAGGCATTACCATTCAGGACATAGGCGTGGGCAAGGACTTCATGTCCAAAACACCAAAAGCAATGGCAACAAAAGACAAAATTGACAAATGGGATCTAATTAAACTAAAGAGCTTCTGCACAGCAAAAGAAACTACCATCAGAGTGAACAGGCAACCTACAACATGGGAGAAAATTTTCGCAACCTACTCATCTGACAAAGGGCTAATATCCAGAATCTACAATGAACTTAAACAAATTTACAAGAAAAAAACAAACAACCCCATCAAAAAGTGGGCGAAGGACATGAACAGACACTTCTCAAAAGAAGACATTTATGCAGCCAAAAAACACATGAAGAAATGCTCATCATCACTGGCCATCAGAGAAATGCAAATCAAAACCACTATGAGATATCATCTCACACCAGTTAGAATGGCAATCATTAAAAAGTCAGGAAACAACAGGTGCTGGAGAGGATGCGGAGAAATAGGAACACTTTTACACTGTTGGTGGGACTGTAAACTAGTTCAACCATTGTGGAAGTCAGTGTGGCGATTCCTCAGGGATCTAGAACTAGAAATACCATTTGACCCAGCCATCCCATTACTGGGTATATACCCAAATGAGTATAAATCATGCTGCTATAAAGACACATGCACACGTATGTTTATTGCGGCACTATTCACAATAGCAAAGACTTGGAACCAACCCAAATGTCCAACAATGATAGACTGGATTAAGAAAATGTGGCACATATACACCATGGAATACTATGCAGCCATAAAAAATGATGAGTTCATATCCTTTGTAGGGACATGGATGAAATTGGAAACCATCATTCTCAGTAAACTATCGCAAGAACAAAAAACCAAACACCGCATATTCTCACTCATAGGTGGGAATTGAACAATGAGATCACATGGACACAGGAAGGGGAATATCACACTCTGGGGACTGTGGTGGGGTCGGGGGAGGGGGGAGGGATAGCATTGGGAGATATACCTAATGCTAGATGACACATTAGTGGGTGCAGCGCACCAGCATGGCACATGTATACATATGTAACTAACCCGATCCGAACAAACGACCCAACACCCGTGCGTTTTATTCTGTCTTTTTATTGCCGATCCCCTCAGAAGAACTCGTCAAGAAGGCGATAGAAGGCGATGCGCTGCGAATCGGGAGCGGCGATACCGTAAAGCACGAGGAAGCGGTCAGCCCATTCGCCGCCAAGCTCTTCAGCAATATCACGGGTAGCCAACGCTATGTCCTGATAGCGGTCCGCCACACCCAGCCGGCCACAGTCGATGAATCCAGAAAAGCGGCCATTTTCCACCATGATATTCGGCAAGCAGGCATCGCCATGGGTCACGACGAGATCATCGCCGTCGGGCATGCGCGCCTTGAGCCTGGCGAACAGTTCGGCTGGCGCGAGCCCCTGATGCTCTTCGTCCAGATCATCCTGATCGACAAGACCGGCTTCCATTCTTGTCCTTGCTCGCTCGATGCGATGTTTCGCTTGGTGGTCGAATGGGCAGGTAGCCGGATCAAGCGTATGCAGCCGCCGCATTGCATCAGCCATGATGGATACTTTCTCGGCAGGAGCAAGGTGAGATGACAGGAGATCCTGCCCCGGCACTTCGCCCAATAGCAGCCAGTCCCTTCCCGCTTCAGTGACAACGTCGAGCACAGCTGCGCAAGGAACGCCCGTCGTGGCCAGCCACGATAGCCGCGCTGCCTCGTCCTGAAGGTGAGTCCAGGAGATGTTTCAGCACTGTTGCCTTTAGTCTCGAGGCAACTTAGACAACTGAGTATTGATCTGAGCACAGCAGGGTGTGAGCTGTTTGAAGATACTGGGGTTGGGGGTGAAGAAACTGCAGAGGACTAACTGGGCTGAGACCCAGTGGCAATGTTTTAGGGCCTAAGGAATGCCTCTGAAAATCTAGATGGACAACTTTGACTTTGAGAAAAGAGAGGTGGAAATGAGGAAAATGACTTTTCTTTATTAGATTTCGGTAGAAAGAACTTTCATCTTTCCCCTATTTTTGTTATTCGTTTTAAAACATCTATCTGGAGGCAGGACAAGTATGGTCATTAAAAAGATGCAGGCAGAAGGCATATATTGGCTCAGTCAAAGTGGGGAACTTTGGTGGCCAAACATACATTGCTAAGGCTATTCCTATATCAGCTGGACACATATAAAATGCTGCTAATGCTTCATTACAAACTTATATCCTTTAATTCCAGATGGGGGCAAAGTATGTCCAGGGGTGAGGAACAATTGAAACATTTGGGCTGGAGTAGATTTTGAAAGTCAGCTCTGTGTGTGTGTGTGTGTGTGTGTGTGTGTGTGTGTGCGCGCACGTGTGTTTGTGTGTGTGTGAGAGCGTGTGTTTCTTTTAACGTTTTCAGCCTACAGCATACAGGGTTCATGGTGGCAAGAAGATAACAAGATTTAAATTATGGCCAGTGACTAGTGCTGCAAGAAGAACAACTACCTGCATTTAATGGGAAAGCAAAATCTCAGGCTTTGAGGGAAGTTAACATAGGCTTGATTCTGGGTGGAAGCTGGGTGTGTAGTTATCTGGAGGCCAGGCTGGAGCTCTCAGCTCACTATGGGTTCATCTTTATTGTCTCCTTTCATCTCAACAGCTCATTCAGGGCACCGGACAGGTCGGTCTTGACAAAAAGAACCGGGCGCCCCTGCGCTGACAGCCGGAACACGGCGGCATCAGAGCAGCCGATTGTCTGTTGTGCCCAGTCATAGCCGAATAGCCTCTCCACCCAAGCGGCCGGAGAACCTGCGTGCAATCCATCTTGTTCAATGGCCGATCCCATATTGGCTACCTCCTCCAGCTGTACGCGTGTCGTATTATACTATGCCGATATACTATGCCGATGATTAATTGTCAACACGTGCTGCAGACGCGTTGCAAAAGCCTAGGCCTCCAAAAAAGCCTCCTCACTACTTCTGGAATAGCTCAGAGGCCGAGGCGGCCTCGGCCTCTGCATAAATAAAAAAAATTAGTCAGCCATGGGGCGGAGAATGGGCGGAACTGGGCGGAGTTAGGGGCGGGATGGGCGGAGTTAGGGGCGGGACTATGGTTGCTGACTAATTGAGATGCATGCTTTGCATACTTCTGCCTGCTGGGGAGCCTGGGGACTTTCCACACCTGGTTGCTGACTAATTGAGATGCATGCTTTGCATACTTCTGCCTGCTGGGGAGCCTGGGGACTTTCCACACCCTAACTGACACACATTCCACAGCTGATCGATACCGTAAGCCGAATTCCAGATGCATGGCGGTAATACGGTTATCCACAGAATCAGGGGATAACGCAGGAAAGAACATGTGAGCAAAAGGCCAGCAAAAGGCCAGGAACCGTAAAAAGGCCGCGTTGCTGGCGTTTTTCCATAGGCTCCGCCCCCCTGACGAGCATCACAAAAATCGACGCTCAAGTCAGAGGTGGCGAAACCCGACAGGACTATAAAGATACCAGGCGTTTCCCCCTGGAAGCTCCCTCGTGCGCTCTCCTGTTCCGACCCTGCCGCTTACCGGATACCTGTCCGCCTTTCTCCCTTCGGGAAGCGTGGCGCTTTCTCATAGCTCACGCTGTAGGTATCTCAGTTCGGTGTAGGTCGTTCGCTCCAAGCTGGGCTGTGTGCACGAACCCCCCGTTCAGCCCGACCGCTGCGCCTTATCCGGTAACTATCGTCTTGAGTCCAACCCGGTAAGACACGACTTATCGCCACTGGCAGCAGCCACTGGTAACAGGATTAGCAGAGCGAGGTATGTAGGCGGTGCTACAGAGTTCTTGAAGTGGTGGCCTAATTACGGCTACACTAGAAGAACAGTATTTGGTATCTGCGCTCTGCTGAAGCCAGTTACCTTCGGAAAAAGAGTTGGTAGCTCTTGATCCGGCAAACAAACCACCGCTGGTAGCGGTGGTTTTTTTGTTTGCAAGCAGCAGATTACGCGCAGAAAAAAAGGATCTCAAGAAGATCCTTTGATCTTTTCTACGGGGTCTGACGCTCAGTGGAACGAAAACTCACGTTAAGGGATTTTGGTCATGAGATTATCAAAAAGGATCTTCACCTAGATCCTTTTAAATTAAAAATGAAGTTTTAAATCAATCTAAAGTATATATGAGTAACCTGAGGCTATGCTGCAGTAAGATACATTGATGAGTTTGGACAAACCACAACTAGAATGCAGTGAAAAAAATGCTTTATTTGTGAAATTTGTGATGCTATTGCTTTATTTGTAACCATTATAAGCTGCAATAAACAAGTTAACAACAACAATTGCATTCATTTTATGTTTCAGGTTCAGGGGGAGGTGTGGGAGGTTTTTTAAAGCAAGTAAAACCTCTACAAATGTGGTATGGCTGATTATGATCGGCGCGGGGCAATGTGCACATGTACCCTAAAACTTAGAGTTTAATTAAAAAAAAAAAAAAAAAAAAAAGAAAGTGGGCATTCTGTCTTGTTCCGGTTCTTAATTAAGGATCCAGACATGATAAGATACATTGATGAGTTTGGACAAACCACAACTAGAATGCAGTGAAAAAAATGCTTTATTTGTGAAATTTGTGATGCTATTGCTTTATTTGTAACCATTATAAGCTGCAATAAACAAGTTAACAACAACAATTGCATTCATTTTATGTTTCAGGTTCAGGGGGAGGTGTGGGAGGTTTTTTAAAGCAAGTAAAACCTCTACAAATGTGGTATGGCTGATTATGATC

TK109-17

|  | Position |
| --- | --- |
| Multi cloning site of pCEP4 | 1-626 |
| Transcriptional strat | 603 |
| 3*FLAG tag sequence | 642-707 |
| ZfL2-1 ORF1 | 708-1613 |
| ZfL2-1 ORF2 | 1614-4697 |
| ZfL2-1 3´UTR | 4698-4836 |
| *mneoI_400_/ColE1* | 4837-7932 |
| ZfL2-1 3´UTR | 7939-8021 |
| SV40 poly A signal of pCEP4 | 8022-8263 |

gttgacattgattattgactagttattaatagtaatcaattacggggtcattagttcatagcccatatatggagttccgcgttacataacttacggtaaatggcccgcctggctgaccgcccaacgacccccgcccattgacgtcaataatgacgtatgttcccatagtaacgccaatagggactttccattgacgtcaatgggtggagtatttacggtaaactgcccacttggcagtacatcaagtgtatcatatgccaagtccgccccctattgacgtcaatgacggtaaatggcccgcctggcattatgcccagtacatgaccttacgggactttcctacttggcagtacatctacgtattagtcatcgctattaccatggtgatgcggttttggcagtacaccaatgggcgtggatagcggtttgactcacggggatttccaagtctccaccccattgacgtcaatgggagtttgttttggcaccaaaatcaacgggactttccaaaatgtcgtaataaccccgccccgttgacgcaaatgggcggtaggcgtgtacggtgggaggtctatataagcagagctcgtttagtgaaccgt**C**AGATCTCTAGAAGCTGGGTACTAGCGGCCGCCACCATGGACTACAAAGACCATGACGGTGATTATAAAGATCATGACATCGACTACAAAGACGATGACGACAAGTCGCTTCCGTCTCTGTCCTTGTGTGCAGGAGAAGCATCGATGGAGGCGTTGGAGCTGGAGCTGGAAGAAGTGGAGTCCCAGATCCGCGCGCTGGTGGTAAGACGGTCGCGGCTACGGGAACGGCTTCTAGCCGTACCTAATGCTAAGGCCGTCTCATCACCTAAGGTACGTGGAAATTACAACCACATCATTCCCTCTACCTCAACCCCGCGTCCTTCTCTGTCCAGGCCCAGCGCACCCGGGGCGCGGCTCAGCCAGGCGTCGTTCACGCCGACACCCGGCTACCACGGCGCCTGGGTGCAGCCGCGCAAGGTGCTTCCCAGATCCCGGGGCAGAACGTCTCCGCCTGTGTTCGAGATCTCCACGGAGAACCGCTTCTCCCCTCTCCGCGAGTCGGGTCCCGATGTGGCCATCATCGGTGACTCGATCGTTCGTCACGTCCGTGCCGCCTCCTCAAAAGGTAATAAAGTACGTACTTTCTGCTTTCCTGGTGCCCGTGTGAAAAATATTTCTACACAGATTCCAACCATCCTGGGCGCTGCCGAGAGCCCTGGTGCCGTTGTCCTCCACGTGGGGACAAACGACACCGGGCTCCGGCAGTCGGAGATCCTGAAGAAGGACTTCAGGAGCCTGATCGAGACGGTTCGACGCACCTCGCCCGCCACGCAGATCATCGTTTCTGGGCCGCTTCCTACCTACCGCCGAGGAAATGAAAGGTTCAGTAGACTTTTAGCTTTGAATGAATGGCTAATAACATGGTGTAAAGAACAGAAATTGCTCTTTGCTAATAACTGGAATCTTTTCTGGGAGCGTCCTAGGCTCTTCCGTCCTGACGGCCTGCACCCCAGTCGAGCCGGAGCTGAACTCCTGTCGGACAACATCTCCAGACTACTTCGCACCATCTGACTAGCAGGTAAAAATTCACAAAATTCACACTATAGCCACCTAGACTCTTGTTCACCCCACTTAAACATCAGTAACGCATATCTGGCGAATCCTATAGAGACTGTGTCggtaccTCGTATTATTAGATTAAGAAATAAACGTACTGTGTGCTCCAGGAAAAATCTAGTAAGAATCAAACCAGAAAAACCAGTAGAAAGTGAAAATACAAATTTCGTAAAACTTGGTCTCCTAAACATCAGGTCACTTGCACCTAAAGCACTTATCATTAATGAAATAATAACAGAAAACAATCTTAATGCACTCTGTCTCACTGAAACCTGGCTGAAACAAAATGACTATATTAGCTTAAATGAAGCAACTCCTCCAGGATTCTTATATAAACATGAGGCTCGTCAAACTGGTCGTGGTGGTGGAGTTGCATCAATCTTTAGTGATTTCCTTAATATTAAACAGAGAAACGGACTTATGTTTAGCTCCTTTGAAGTATTATCGCTTAATGTTCAGCTTCCAGATACTATACAAAAACCTATGTTATCTCTCGCTTTAATCACCATATATAGACCCCCAGGACCCTATGTCAAATTTCTAAAAGAATTTTCTGATTTTATTTCTGACTTACTAGTCAAAACTGATAAAATGCTAATTGTAGGTGACTTTAACATCCACATAGATGACGCTAATGATACATTAGGGCTCGCGTTTATGGATTTAATACACTCACTTGGGATAAAGCAAAACGTTGTGGGTCCAACCCATCGCTTAAAGCATACATTAGATCTAATTCTGTCTTATGGAATCGAGGTTATTGACGTAGACATTATACCACAAAGTGATGATATTACAGATCACTACCTCTTACTATATAAGCTATGTTTACCTGAAATCAGCAAACCCGCTCCAATACTCCGCCCTAGTAGAACTATTGTTCCGTCAACTAAAGATGAATTTATAAATAACTTACCTGATCTCTCTCTATTTCGTAATGCACCCGCAAACTCAAATGATCTTGATGTAGTAACCGGCAGTATGGATGCCATCTTTACTAGCACACTAAATACTGTGGCACCCATAAAATTAAAAAAGGCTAGAGAGATTAAAACTATACCATGGTATAATAGTCATACTCGTGCGCTCAAAACAGCAACCCGCGCCCTGGAACGTAAATGGAAAAAAACTAATTTAGAGGTCTTTAGAATTGCGTACAAAGACAGTATGTCCAGCTATAGGAGGGCTCTAAAATCTGCCAGGACCGAGCACCTGCGCAAACTGATAGAAAATAATCATAACAATCCTAGATTTTTATTTAACACCATCTCTAAATTAGCAAATAATCGGTCATCCTTGGAACAAACTACTCCACCGCAAATTAGTAGTGATGACTTCATGAATTTTTTCAGTAATAAAATAGAAGGCTTTAGACAGAAAATAGGAGATGCCAAACTTTCTGCACCGGCTTATACTCCAAATCCTGTAAATATTTCATTAAATCATAATAATAACCTACACTGCTTCAAAATCATAGAACATGAAGAGTTAGTGAAAATTATAAATAGCTCTAAACCAGCTACGTGTATGCTGGACTCAATTCCAACAAAATTACTGAAAGAGCTGCTACCTGCTATAGGAGAACCTCTTCTTAACATTATCAACTCTTCTTTATCTATAGGCCATGTTCCAAACTCTTACAAGCTAGCTGTTATTAAGCCTATTATTAAGAAACCGCAACTAGACACCAACAACTTAGCTAACTATAGGCCTATTTCAAATCTTCCATTTATGTCTAAAATACTAGAAAAAGTTGTTTCCACTCAATTATGCTCTTTTCTGCAGACGAACAATATTTTTGAAGTGTTTCAGTCAGGTTTCAGGGCTCACCACAGTACAGAAACCGCCTTAGTGAAAATAACCAACGATTTACTCTTAGCTGCTGACCGAGGGTGCGTCTCGCTATTAGTTTTACTCGATCTTAGTGCGGCATTTGATACCATTGACCACAATATCCTCATAAATCGCTTAAAGTCTACAGGTGTCCAGGGACAGGCTCTACAATGGTTTAAGTCATACTTAACTGACCGCTACCAGTTTGTAAATCTTAATGGACAGCCTTCACAAATCTGCCCAGTAAAGTATGGGGTGCCTCAAGGATCAGTTTTAGGCCCTTTACTGTTTACAATTTACATGCTACCTATGGGAGACATTATTAGAAGACATGGGATCAGCTTTCACTGCTATGCAGATGATACTCAATTATATATTTCCACTAAACCTGACGAGACGTCTGAACTTTCTAAACTAACTGAGTGTATCAAAGACATCAAAGACTGGATGACCAACAATTTTCTTCTCTTAAACTCAGACAAAACAGAATTATTACTTATTGGGCCTAAATCTTGCACACAGCAGATCTCGCAACTCAATTTACAATTAGAGGGATACAAAGTTAGCTTTAGCTCTACTATAAAAGATTTGGGTGTCATATTAGACAGCAATCTAACTTTTAAAAACCATATATCCCATGTCACAAAAACTGCCTTCTTTCATCTGAGAAATATCGCTAAATTACGAAATATGCTATCCATCTCAGATGCAGAAAAGCTAGTCCATGCTTTTATGACTTCGAGACTGGATTACTGTAATGCTCTATTTGCTGGCTGCCCAGCATCCTCTATTAACAAACTTCAATTAGTGCAAAATGCAGCAGCCAGAGTTCTGACCAGGTCTAGAAAATATGATCATATAACCCCAATTTTATCCTCCTTACACTGGCTGCCTGTTAAATTTCGTATTGAATTTAAAATATTACTTCTCACCTATAAAGCTCTAAATAATCTAGCTCCTGTTTATCTAACCAACCTTCTGTCTCGCTACGAACCAACTCGCTCCTTAAGATCTCAAAATTCAGGGCTTCTGGTAGTACCTAGAATAGCAAAATCAAGTAAAGGAGGTCGAGCCTTCTCTTTCATGGCTCCTACACTCTGGAATAGCCTTCCTGGTAATGTCCGAGGCTCAGACACACTCTCCCAGTTCAAAACTAGATTAAAGACCTATCTGTTTAGTAAAGCATACACTCAGTGCATCACCTAGCAGGTTCCACACTGCCTTCTACATCTTGCTTATATACACTATGAACAGCAGCTACGCTAATTATTCTCTTTATTCTCTATTTTCACCTGGGGATACTCATCCCGAGGTCCTCAGATTAGGCGGAGTCACTGATTGGATCTACAAACGACCCAACACCCGTGCGTTTTATTCTGTCTTTTTATTGCCGATCCCCTCAGAAGAACTCGTCAAGAAGGCGATAGAAGGCGATGCGCTGCGAATCGGGAGCGGCGATACCGTAAAGCACGAGGAAGCGGTCAGCCCATTCGCCGCCAAGCTCTTCAGCAATATCACGGGTAGCCAACGCTATGTCCTGATAGCGGTCCGCCACACCCAGCCGGCCACAGTCGATGAATCCAGAAAAGCGGCCATTTTCCACCATGATATTCGGCAAGCAGGCATCGCCATGGGTCACGACGAGATCATCGCCGTCGGGCATGCGCGCCTTGAGCCTGGCGAACAGTTCGGCTGGCGCGAGCCCCTGATGCTCTTCGTCCAGATCATCCTGATCGACAAGACCGGCTTCCATCCGAGTACGTGCTCGCTCGATGCGATGTTTCGCTTGGTGGTCGAATGGGCAGGTAGCCGGATCAAGCGTATGCAGCCGCCGCATTGCATCAGCCATGATGGATACTTTCTCGGCAGGAGCAAGGTGAGATGACAGGAGATCCTGCCCCGGCACTTCGCCCAATAGCAGCCAGTCCCTTCCCGCTTCAGTGACAACGTCGAGCACAGCTGCGCAAGGAACGCCCGTCGTGGCCAGCCACGATAGCCGCGCTGCCTCGTCCTGAAGGTGAGTCCAGGAGATGTTTCAGCACTGTTGCCTTTAGTctcgaggcaacttagacaactgagtattgatctgagcacagcagggtgtgagctgtttgaagatactggggttgggggtgaagaaactgcagaggactaactgggctgagacccagtggcaatgttttagggcctaaggaatgcctctgaaaatctagatggacaactttgactttgagaaaagagaggtggaaatgaggaaaatgacttttctttattagatttcggtagaaagaactttcatctttcccctatttttgttattcgttttaaaacatctatctggaggcaggacaagtatggtcattaaaaagatgcaggcagaaggcatatattggctcagtcaaagtggggaactttggtggccaaacatacattgctaaggctattcctatatcagctggacacatataaaatgctgctaatgcttcattacaaacttatatcctttaattccagatgggggcaaagtatgtccaggggtgaggaacaattgaaacatttgggctggagtagattttgaaagtcagctctgtgtgtgtgtgtgtgtgtgtgtgtgtgtgtgtgtgcgcgcacgtgtgtttgtgtgtgtgtgagagcgtgtgtttcttttaacgttttcagcctacagcatacagggttcatggtggcaagaagataacaagatttaaattatggccagtgactagtgctgcaagaagaacaactacctgcatttaatgggaaagcaaaatctcaggctttgagggaagttaacataggcttgattctgggtggaagctgggtgtgtagttatctggaggccaggctggagctctcagctcactatgggttcatctttattgtctcctttcatctcaacagCTCATTCAGGGCACCGGACAGGTCGGTCTTGACAAAAAGAACCGGGCGCCCCTGCGCTGACAGCCGGAACACGGCGGCATCAGAGCAGCCGATTGTCTGTTGTGCCCAGTCATAGCCGAATAGCCTCTCCACCCAAGCGGCCGGAGAACCTGCGTGCAATCCATCTTGTTCAATGGCCGATCCCATATTGGCTACCTCCTCCAGCTGTACGCGTGTCGTATTATACTATGCCGATATACTATGCCGATGATTAATTGTCAACACGTGCTGCAGACGCGTTGCAAAAGCCTAGGCCTCCAAAAAAGCCTCCTCACTACTTCTGGAATAGCTCAGAGGCCGAGGCGGCCTCGGCCTCTGCATAAATAAAAAAAATTAGTCAGCCATGGGGCGGAGAATGGGCGGAACTGGGCGGAGTTAGGGGCGGGATGGGCGGAGTTAGGGGCGGGACTATGGTTGCTGACTAATTGAGATGCATGCTTTGCATACTTCTGCCTGCTGGGGAGCCTGGGGACTTTCCACACCTGGTTGCTGACTAATTGAGATGCATGCTTTGCATACTTCTGCCTGCTGGGGAGCCTGGGGACTTTCCACACCCTAACTGACACACATTCCACAGCTGATCGATACCGTAAGCCGAATTCCAGATGCATGGCGGTAATACGGTTATCCACAGAATCAGGGGATAACGCAGGAAAGAACATGTGAGCAAAAGGCCAGCAAAAGGCCAGGAACCGTAAAAAGGCCGCGTTGCTGGCGTTTTTCCATAGGCTCCGCCCCCCTGACGAGCATCACAAAAATCGACGCTCAAGTCAGAGGTGGCGAAACCCGACAGGACTATAAAGATACCAGGCGTTTCCCCCTGGAAGCTCCCTCGTGCGCTCTCCTGTTCCGACCCTGCCGCTTACCGGATACCTGTCCGCCTTTCTCCCTTCGGGAAGCGTGGCGCTTTCTCATAGCTCACGCTGTAGGTATCTCAGTTCGGTGTAGGTCGTTCGCTCCAAGCTGGGCTGTGTGCACGAACCCCCCGTTCAGCCCGACCGCTGCGCCTTATCCGGTAACTATCGTCTTGAGTCCAACCCGGTAAGACACGACTTATCGCCACTGGCAGCAGCCACTGGTAACAGGATTAGCAGAGCGAGGTATGTAGGCGGTGCTACAGAGTTCTTGAAGTGGTGGCCTAATTACGGCTACACTAGAAGAACAGTATTTGGTATCTGCGCTCTGCTGAAGCCAGTTACCTTCGGAAAAAGAGTTGGTAGCTCTTGATCCGGCAAACAAACCACCGCTGGTAGCGGTGGTTTTTTTGTTTGCAAGCAGCAGATTACGCGCAGAAAAAAAGGATCTCAAGAAGATCCTTTGATCTTTTCTACGGGGTCTGACGCTCAGTGGAACGAAAACTCACGTTAAGGGATTTTGGTCATGAGATTATCAAAAAGGATCTTCACCTAGATCCTTTTAAATTAAAAATGAAGTTTTAAATCAATCTAAAGTATATATGAGTAACCTGAGGCTATGCTGCAGCGGATCCTGACCATTTATGTGAAGCTGCTTTGACACAATCTACATTGTAAAAGCGCTATACAAATAAAGCTGAATTGAATTGAATTGAATggatccAGACATGATAAGATACATTGATGAGTTTGGACAAACCACAACTAGAATGCAGTGAAAAAAATGCTTTATTTGTGAAATTTGTGATGCTATTGCTTTATTTGTAACCATTATAAGCTGCAATAAACAAGTTAACAACAACAATTGCATTCATTTTATGTTTCAGGTTCAGGGGGAGGTGTGGGAGGTTTTTTAAAGCAAGTAAAACCTCTACAAATGTGGTATGGCTGATTATGATC

Nb2A3-2

|  | Position |
| --- | --- |
| Multi cloning site of pCEP4 | 1-626 |
| Transcriptional strat | 603 |
| Nimb2 ORF1 | 638-1876 |
| Nimb2 ORF2 | 1877-5638 |
| *mneoI_400_/ColE1* | 5639-8749 |
| Nimb2 3´ UTR | 8756-8901 |
| SV40 poly A signal of pCEP4 | 8902-9143 |

GTTGACATTGATTATTGACTAGTTATTAATAGTAATCAATTACGGGGTCATTAGTTCATAGCCCATATATGGAGTTCCGCGTTACATAACTTACGGTAAATGGCCCGCCTGGCTGACCGCCCAACGACCCCCGCCCATTGACGTCAATAATGACGTATGTTCCCATAGTAACGCCAATAGGGACTTTCCATTGACGTCAATGGGTGGAGTATTTACGGTAAACTGCCCACTTGGCAGTACATCAAGTGTATCATATGCCAAGTCCGCCCCCTATTGACGTCAATGACGGTAAATGGCCCGCCTGGCATTATGCCCAGTACATGACCTTACGGGACTTTCCTACTTGGCAGTACATCTACGTATTAGTCATCGCTATTACCATGGTGATGCGGTTTTGGCAGTACACCAATGGGCGTGGATAGCGGTTTGACTCACGGGGATTTCCAAGTCTCCACCCCATTGACGTCAATGGGAGTTTGTTTTGGCACCAAAATCAACGGGACTTTCCAAAATGTCGTAATAACCCCGCCCCGTTGACGCAAATGGGCGGTAGGCGTGTACGGTGGGAGGTCTATATAAGCAGAGCTCGTTTAGTGAACCGT**C**AGATCTCTAGAAGCTGGGTACTAGCTAGCGGAGGATGGCAAGGCGGATAGACTCCAAGGCGGTTGGCAACAACTGGAATGTGAGTGGACAGGAGAATGCAGACGGAAGTGACGAATCAGGAAATGAAGTAGGAGATATGGAAGTAGTTTGCGGGAACTATGAAAAATGGGACATGGTTAAAAACAACAAACGGAAAAAGAAACGAAGAAATAAATCTGATGAAAGTGATTCGGATAGATGCTCAGCCCTAGAGGAAACGGTGACAGTGGAATACAAGGTATTTGCTAAACCTGTGCAAGAAGGGGACACATTTGGAGGTATGAATCCGATACAACTAACCAAAACACTGCATAAGGAAATAGGTATAATAAAGAGTGCAAAAATCTTGAGAAACGGATCGATACTGATTTTCTGTAAAGATGAAAAACAGCAAGCTAAAGCAATTAAGGTGAATAAAATCAATGGAAAAAAAGTAGAATGTTCCAAGACAAACGGGAAAAGATATGTTAAAGGCGTTGTGACAGGAATTCCTGTCAATGTTTCGGCAGATGAAGTGAAAGCAAGCATAACAAATGCAAATGTGGTCGAAGCTAAACGCTTAAGAACAAACAGAAATGGAAGTGCTTGTGATAGTCTTTCTGTTATGGTCACATTTGATGAGGAGACACTCCCGAAAAAGATATTAATCGGGTATATGTGCTATGATGTAAGACTGTACATTCCCCCGCCCCTTCGGTGTTATAAATGTCAGAGATTTGGACATATAGCAGCTTTTTGCAAGGGAAAGCAAAGGTGTGGTAAATGTAGTGGGGAACACGAATATGGAAAATGTGAAGAGGGTGCAAAGTTGAAATGTTGCAACTGTGGAGGGGATCATAGCTCAGCGTATCGAGGGTGTGAGGTTAGCAAAAGGCAGGCAGAAGTACAAAGAGTAAAAGCTGTTCAAGGAATCAGCTATGCGGAAGCATTAAAAAAGGTTCCAGAAATTATGACTGTGTCTAAACAAAATGAAAACAGGAACAAAAATACAGAGACATGTCAAAAATGTGAACAACTGAAAGCGGAAACTCTGATAGTGAGCAAAAATGATTTTGTAATATTCATGGCAGAGATAATAAATTGTTCCGCTCAAACGAAAAGTAGGAATGAAAGAATCAAAATAATAATCAAATCAGCAGAAAAATACCTGGATGTGAAGGATCTGCTCTGGGAAACGGTTAGGGACATCTTAAATGAAGATACACAATCATCCCAGCCTGGGGGAGGAACGTCTTAAATGTTAAACGTTCTACAATGGAATGCAAGGAGCTTAATTGCGAATGGCCAAGAATTTAAAAAATATATTGATAATATTATAGAGAAACCTAATATAATATGCATACAAGAAACATGGTTAAAACCTCAATTAGATTTTAATATTAAGGGATATAATATAGTTAGAAATGATAGGAATCATAGCAGAGGAGGAGGAATTGCAACATTTATAAAAAGCGGAATGAAGTTTAGGATAGAACAAATAAATACAAAGTATGAATCAATTTTAATCAAGGTATGGACGGATAGAGGATGTATAGACATTATTAATTATTATAATCCCTGTGATAAATTAAATCAAAACATATTAGAAGAAGTAATGGGTGTACGACAAGACAGTGTTTTATGGTGTGGGGATTTTAATTCACATAATTCATTATGGGGGAGTAATAGTAATGATGCAAATGGGATTCTTCTTGAAGAATTTATAGATGAAAAATATTTAGTTTGTCTAAATAATGGTGAAGGCACACGGTATAATTGTTTTAAAAATACAGAAAGCGTACTAGACTTAACATTTATTAGTAGTTCATTAGCAGCGGTTAGCACATGGAAAGTACTCAAGCACAATACAATAGGTAGTGACCATTACCCAGTAGTAACTAAAATTGGATTAAAGATAATGTATGAAAAAGAAGATAGAATTCCAAGATGGAAATTGGAGAAAGTAAATTGGAAAGAATTTCAAGAATTATGTGATAAGAGAGTTATGACAATACAAATAATAAACCAGAGAGATGTAAACATTTTAAATAATAAAATTGTTAATGAAATAATTCAAGCAGCCGAAGAAATAATACCTAAAAGTAAAGGAGTTGGTTGTACTAAAAATGTACCTTGGTGGAATAATGATTGTAAAGCAGCTATTAAAGCAAGAAATAAAGCATTTAGACATCTTAAAAAACACCATTCACTGGAAGCTATGATCATGTATAAAAAGGCTCAGACAATATTAAGAAAAACTATTAAAACACAAAAGCGTATATTCTGGAGAGAATATTGCAATAGTCTTGGACGAGAGGTACAATTATCAGAGGTGTGGGGTATGATTAGAAGAATGGCAGGTGTTAGACGAAATTATGAATTACCAGTGTTACAGTATGGCGATATAGTAGCGATCAGCAACCTAGAAAAGGCGGAACTATTAGTCCAACATTTTAGAAATGTACATAGTTCAGATAATCTCTCTGAAGAGGCTAGGAAATGCAGAAATACTACATTAACTAAACATCCTGATCTTTTAAAGAAAGTAAAAACAACAGAAAATCCTTTAGATTTACAGTTTAATATGTTTGAATTAAAACGAGCAATCATTAGCGCAAGACAAACTGCCCCAGGTAAGGATGAAATATGCTATAAAATGTTGTCACACATGTCAGAGGCATCACTAGAAATAGTACTAAATTTATTTAATCAAATTTGGGATATGGGTCAATTACCTATAGCATGGAAACAATCTATAGTAGTACCTATACTTAAACCAGGGAAAAATCCATCAGATCCTTCTAGCTATAGACCCATTTCACTGACATCCCACTTATGCAAAATAATGGAAAGAATGATTACAGAAAGAATTACATATTTTTTAGAAAGCAAAAACCTTTTTTCTCCATATCAGAGTGGGTTCCGCAGAGGCAGAAATACTATGGACTCAGTATTATGTTTGGAATCAGACATCAGGAAAGCACAGACTAACAAAGAAGTTGTGATAGCTATCTTTTTTGATATAGAAAAGGCATACGATATGCTTTGGAAAGAGGGATTGCTGATTAAATTAAAATCTTTAGGAGTCACTGGCAAAACATATAATTGGGTCATGGATTTCTTATTTGGAAGAAAAATACAAGTTAGGGTAGGAAAAGAATATTCACATGAATACACAGTGGAAAATGGAACTCCTCAAGGTAGTGTTTGCAGCCCATTATTGTTCAATATAATGATAAACGACATTTTCTCTCAGATTGAACAAAGTATAGGAAAATCATTGTATGCAGATGATGGGGCACTATGGATTAGAGGTCGGAATGTGTCATTTGTACAAAAGAAAATACAGAATGCAATATTTGAGGTAGAAAAATGGGCAAATAAATGGGGATTCAAACTATCTGTTGCAAAAACTCAGGTCATCTGTTTCTCAAGACGACATAAAACCATATCACTCGCTTTAAAACTATATGGACAACCACTAGAACAAGTAAAAACTGTAAGGTTTCTTGGGGTTTGGTTCGATGAAAAGCTGACATGGAAGGATCATCTGAATAAAATCACAGAAAAAAGTAAAAAGGTTATTAATGTACTTCGTTGCCTGTCAGGGCAAGAGTGGGGAGCGAGTAGAACATCACTACAAAATATATACTGGGCCCTCATGAGATCTGTTTTTGATTATGGATGTATAGCTTACATGTCGGCAGCAGAGTCAAACCTCAAAAAATTAGATGTATTACAAGCACAGGCCCTTAGAATCTGTAGTGGATCATTTAGAACCTCACCAGTTTCATCTATGCAGGTTGAAATGGGAGAAATGCCCTTAAGCATTAGGAGAATGAAATTAATGATGGCATATTGGGTTAACATTCAGGGACAAATTGAATCACATCCAACAAAAAGAACATTACTAGAATGTTGGGAGCATGAGGAAACTAATTTCATAAGTTTCGGATGGATAGCTGAGGCAAAAGCCAGAATAATTGGATTAGATCAACTGCAATACTGTAAGGCAGTCCCAATCCCATATATTCCGCCATGGTTTTTTCCATTACCTAAAGTAGATTTCAATATACAACAGGAGTTAAAAGATAACAATAATATTCTTCCAACAAAATATATAGTGCAGAATTATTTAGAAAAAAATTATAAAGAATCAATATTTTTATTCACAGATGGATCTAAAGACCCACAAACAGGCCATACAGGCGCAGCAGTGTATATCCCAGTTAATCAATACCATATTAAGAAAAGAATAACTAATAATATATCAGTATATACAACAGAGTTAATTGCAATACTAATTGCCCTGCAGTGGATTGAGGAGAATGACATATATAATGTAGTAATTGCATCAGATAGCTTTTCATCACTGGAAAGTATAAGATCTGGAAGGTCTTCATACAGAATGGATATTCTTAATAATATACTCAGTAAGACATATTATATTAAAATGAAGGGCAAATCAGTATATTTCATTTGGGTTCCTGCTCACGTAGGTGTGGAAGGTAATGAGAAGGCAGACTTTCTGGCCAAACAAGCTCTTAGAATTAGTAAAGTAAATCTAGAAGTCCCATTGAGCAAAGCAGAAGCTAAAATCATGATAAGAACATATGCACAATCAATATGGCAAGTACACTGGGATAACATTGATACAGGTAGACATTTATATAATATACAGAAACAAGTTGGAACTGGGAGGAAGGAGAACAGAAATCGTAGGGAAGGAAGTATTATAACTCGGATGAGAATAGGTCACACTGGACTCAATCATACATTACATAAAATAGGGAAACACCCAACTGGGCAATGTATACATTGTAATCAACAAGAAACAATAGAACATATTTTATTTCACTGTAGTAATTATAATAAAGAACGAAACGATCTTATTCAGTCAGTTAAAAAGAGCAATCTTCAGCATTTTACATTGGCAGGTTTATTAGGAAACAAATCTAGTGAGGTATATAATGATATAATAAAATTTATTAAAGAAACCCAGTTAGAGGAAAGAATATAGAGAGCGGCCGCGCACAAACGACCCAACACCCGTGCGTTTTATTCTGTCTTTTTATTGCCGATCCCCTCAGAAGAACTCGTCAAGAAGGCGATAGAAGGCGATGCGCTGCGAATCGGGAGCGGCGATACCGTAAAGCACGAGGAAGCGGTCAGCCCATTCGCCGCCAAGCTCTTCAGCAATATCACGGGTAGCCAACGCTATGTCCTGATAGCGGTCCGCCACACCCAGCCGGCCACAGTCGATGAATCCAGAAAAGCGGCCATTTTCCACCATGATATTCGGCAAGCAGGCATCGCCATGGGTCACGACGAGATCATCGCCGTCGGGCATGCGCGCCTTGAGCCTGGCGAACAGTTCGGCTGGCGCGAGCCCCTGATGCTCTTCGTCCAGATCATCCTGATCGACAAGACCGGCTTCCATTCTTGTCCTTGCTCGCTCGATGCGATGTTTCGCTTGGTGGTCGAATGGGCAGGTAGCCGGATCAAGCGTATGCAGCCGCCGCATTGCATCAGCCATGATGGATACTTTCTCGGCAGGAGCAAGGTGAGATGACAGGAGATCCTGCCCCGGCACTTCGCCCAATAGCAGCCAGTCCCTTCCCGCTTCAGTGACAACGTCGAGCACAGCTGCGCAAGGAACGCCCGTCGTGGCCAGCCACGATAGCCGCGCTGCCTCGTCCTGAAGGTGAGTCCAGGAGATGTTTCAGCACTGTTGCCTTTAGTCTCGAGGCAACTTAGACAACTGAGTATTGATCTGAGCACAGCAGGGTGTGAGCTGTTTGAAGATACTGGGGTTGGGGGTGAAGAAACTGCAGAGGACTAACTGGGCTGAGACCCAGTGGCAATGTTTTAGGGCCTAAGGAATGCCTCTGAAAATCTAGATGGACAACTTTGACTTTGAGAAAAGAGAGGTGGAAATGAGGAAAATGACTTTTCTTTATTAGATTTCGGTAGAAAGAACTTTCATCTTTCCCCTATTTTTGTTATTCGTTTTAAAACATCTATCTGGAGGCAGGACAAGTATGGTCATTAAAAAGATGCAGGCAGAAGGCATATATTGGCTCAGTCAAAGTGGGGAACTTTGGTGGCCAAACATACATTGCTAAGGCTATTCCTATATCAGCTGGACACATATAAAATGCTGCTAATGCTTCATTACAAACTTATATCCTTTAATTCCAGATGGGGGCAAAGTATGTCCAGGGGTGAGGAACAATTGAAACATTTGGGCTGGAGTAGATTTTGAAAGTCAGCTCTGTGTGTGTGTGTGTGTGTGTGTGTGTGTGTGTGTGCGCGCACGTGTGTTTGTGTGTGTGTGAGAGCGTGTGTTTCTTTTAACGTTTTCAGCCTACAGCATACAGGGTTCATGGTGGCAAGAAGATAACAAGATTTAAATTATGGCCAGTGACTAGTGCTGCAAGAAGAACAACTACCTGCATTTAATGGGAAAGCAAAATCTCAGGCTTTGAGGGAAGTTAACATAGGCTTGATTCTGGGTGGAAGCTGGGTGTGTAGTTATCTGGAGGCCAGGCTGGAGCTCTCAGCTCACTATGGGTTCATCTTTATTGTCTCCTTTCATCTCAACAGCTCATTCAGGGCACCGGACAGGTCGGTCTTGACAAAAAGAACCGGGCGCCCCTGCGCTGACAGCCGGAACACGGCGGCATCAGAGCAGCCGATTGTCTGTTGTGCCCAGTCATAGCCGAATAGCCTCTCCACCCAAGCGGCCGGAGAACCTGCGTGCAATCCATCTTGTTCAATGGCCGATCCCATATTGGCTACCTCCTCCAGCTGTACGCGTGTCGTATTATACTATGCCGATATACTATGCCGATGATTAATTGTCAACACGTGCTGCAGACGCGTTGCAAAAGCCTAGGCCTCCAAAAAAGCCTCCTCACTACTTCTGGAATAGCTCAGAGGCCGAGGCGGCCTCGGCCTCTGCATAAATAAAAAAAATTAGTCAGCCATGGGGCGGAGAATGGGCGGAACTGGGCGGAGTTAGGGGCGGGATGGGCGGAGTTAGGGGCGGGACTATGGTTGCTGACTAATTGAGATGCATGCTTTGCATACTTCTGCCTGCTGGGGAGCCTGGGGACTTTCCACACCTGGTTGCTGACTAATTGAGATGCATGCTTTGCATACTTCTGCCTGCTGGGGAGCCTGGGGACTTTCCACACCCTAACTGACACACATTCCACAGCTGATCGATACCGTAAGCCGAATTCCAGATGCATGGCGGTAATACGGTTATCCACAGAATCAGGGGATAACGCAGGAAAGAACATGTGAGCAAAAGGCCAGCAAAAGGCCAGGAACCGTAAAAAGGCCGCGTTGCTGGCGTTTTTCCATAGGCTCCGCCCCCCTGACGAGCATCACAAAAATCGACGCTCAAGTCAGAGGTGGCGAAACCCGACAGGACTATAAAGATACCAGGCGTTTCCCCCTGGAAGCTCCCTCGTGCGCTCTCCTGTTCCGACCCTGCCGCTTACCGGATACCTGTCCGCCTTTCTCCCTTCGGGAAGCGTGGCGCTTTCTCATAGCTCACGCTGTAGGTATCTCAGTTCGGTGTAGGTCGTTCGCTCCAAGCTGGGCTGTGTGCACGAACCCCCCGTTCAGCCCGACCGCTGCGCCTTATCCGGTAACTATCGTCTTGAGTCCAACCCGGTAAGACACGACTTATCGCCACTGGCAGCAGCCACTGGTAACAGGATTAGCAGAGCGAGGTATGTAGGCGGTGCTACAGAGTTCTTGAAGTGGTGGCCTAATTACGGCTACACTAGAAGAACAGTATTTGGTATCTGCGCTCTGCTGAAGCCAGTTACCTTCGGAAAAAGAGTTGGTAGCTCTTGATCCGGCAAACAAACCACCGCTGGTAGCGGTGGTTTTTTTGTTTGCAAGCAGCAGATTACGCGCAGAAAAAAAGGATCTCAAGAAGATCCTTTGATCTTTTCTACGGGGTCTGACGCTCAGTGGAACGAAAACTCACGTTAAGGGATTTTGGTCATGAGATTATCAAAAAGGATCTTCACCTAGATCCTTTTAAATTAAAAATGAAGTTTTAAATCAATCTAAAGTATATATGAGTAACCTGAGGCTATGCTGCAGCGGATCCTCCCCCTTTCTCTCCCTTCATGTAATTTATACACTTCAGAAAGTATTCAACTAGTTCCATACTCCATTCCAGTCGGTGGCGGTAATGCACCTATAAGTCTGGTTGCCAACCGCCAGTAAAACCCAAAGAAGAAGAAGAAGAAGAAGGGATCCAGACATGATAAGATACATTGATGAGTTTGGACAAACCACAACTAGAATGCAGTGAAAAAAATGCTTTATTTGTGAAATTTGTGATGCTATTGCTTTATTTGTAACCATTATAAGCTGCAATAAACAAGTTAACAACAACAATTGCATTCATTTTATGTTTCAGGTTCAGGGGGAGGTGTGGGAGGTTTTTTAAAGCAAGTAAAACCTCTACAAATGTGGTATGGCTGATTATGATC
